# Manganese-catalyzed allylation *via* sequential C–H and C–C/C–Het bond activation†
†Electronic supplementary information (ESI) available. See DOI: 10.1039/c7sc00230k



**DOI:** 10.1039/c7sc00230k

**Published:** 2017-02-24

**Authors:** Qingquan Lu, Felix J. R. Klauck, Frank Glorius

**Affiliations:** a Organisch-Chemisches Institut , Westfälische Wilhelms-Universität Münster , Corrensstraße 40 , 48149 Münster , Germany . Email: glorius@uni-muenster.de

## Abstract

Manganese-catalyzed sequential C–H and C–C/C–Het bond activation to synthesize allylic alcohols, allylated arenes, functionalized cyclopentenes and skipped dienes is reported. This protocol can be readily scaled up and various coupling partners are applied in manganese catalysis for the first time. Moreover, manganese-catalyzed alkenyl C(sp^2^)–H activation is also shown. Complimentary to the standard solution-based protocols, these reactions also proceed efficiently under neat conditions, which is unprecedented for abundant metal catalyzed C–H activation reactions.

## Abstract 2

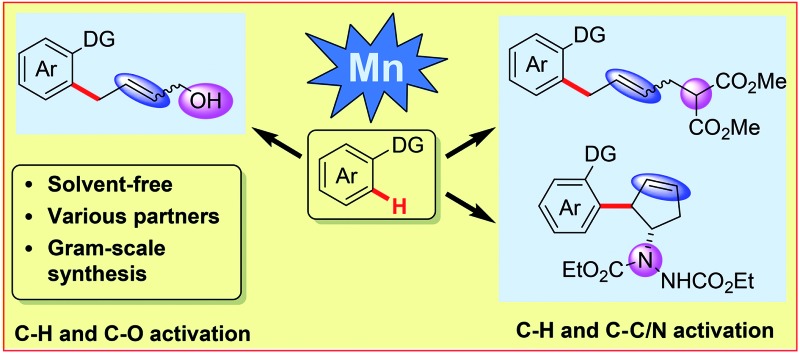
Manganese-catalyzed sequential C–H and C–C/C–Het bond activation to synthesize allylic alcohols, allylated arenes, functionalized cyclopentenes and skipped dienes is reported.

Complimentary to the noble fifth- and sixth-row metals, direct C–H activation^1^ using 3d-transition metal catalysis has fascinated chemists owing to their abundance, low price and low toxicity, as well as to their potential to promote novel reactivity.^2^ Over the past years, the goal of achieving sustainability in organic synthesis has propelled important research in this field and significant progress has been made. Base-metals with flexible redox ability, such as Fe,^3^ Co,^4^ Ni,^5^ and Cu^6^ are extensively used in organometallic C–H activation today. In contrast to being the third most abundant transition metal, manganese is comparatively underutilized.^7^

Manganese-mediated stoichiometric C–H activation has been explored since the 1970s, however, catalytic variants of these reactions have proved challenging.^7^ Recently, the groups of Kuninobu and Takai, Wang, Ackermann and others have significantly advanced this field of research.^8^ Manganese catalysts have been found to be versatile as they can display unique reactivity and enable C–H functionalization with a variety of coupling partners containing polar multiple bonds.^8^ Mechanistically, these reactions mainly involve the formal addition of a metallacycle to an unsaturated reaction partner or a substitution reaction.^8^ In recent years, considerable efforts have been made to develop processes that can merge C–H activation with challenging C–C/C–Het cleavage reactions, which could allow for the efficient introduction of two different functional groups into one molecule in a single step.^9^ However, most of the examples reported to date suffer from the requirement for precious transition metal catalysts and stoichiometric activators. Very recently, a manganese-catalyzed substitutive C–H allylation through highly selective C–H/C–O functionalization was achieved by Ackermann *et al.* ^8*k*^ Owing to our continuous interest in 3d-transition metal catalysis, we questioned whether manganese catalysis can serve as an alternative route to integrating C–H activation with β-carbon/-hetero atom elimination, which is largely unexplored in this field.

To date, cyclometalation has been the most straightforward and common method for the activation of C–H bonds. Such processes rely mainly on solvent-based techniques. From a sustainability perspective, solvent-free C–H activation processes are highly desirable. Recently, rhodium(iii) and iridium(iii) catalyzed C–H functionalizations under solvent-free conditions using a ball mill have been reported by Bolm and co-workers.^10^ However, to the best of our knowledge, first-row transition metal catalyzed C–H activation under neat conditions has not been developed thus far. Herein, our manganese catalyzed coupling offers an environmentally friendly, operationally simple alternative to the more traditional solvent-based protocols, featuring a cheap catalytic system. In this report, various coupling partners, including vinyl-1,3-dioxolan-2-one, 2-vinyloxirane, vinylcyclopropane and diazabicycle, are applied in manganese catalysis for the first time, leading to the convenient synthesis of allylic alcohols, allylated arenes, functionalized cyclopentenes and skipped dienes (Scheme 1).

**Scheme 1:**
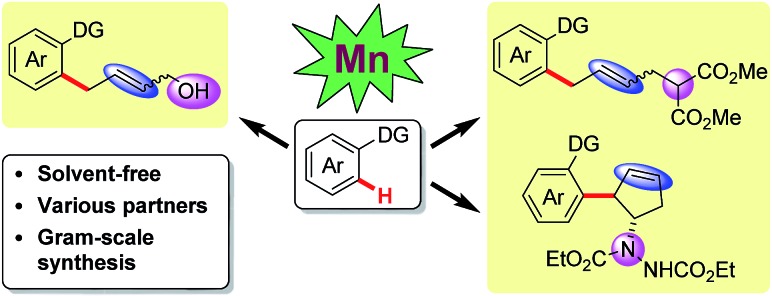
Mn-catalyzed sequential C–H and C–C/C–Het activation.

We started our investigation by reacting *N*-(2-pyridyl)-indole (**1a**) with vinyl-1,3-dioxolan-2-one (**2aa**) under [Mn_2_(CO)_10_] catalysis in diethyl ether at 80 °C. This transformation involves the cleavage of a stable C–O bond to form an easily modifiable C=C bond and an alcohol moiety. To our delight, the desired product **3a** could be isolated in 41% yield (for details, see Table S1 in the ESI†). Subsequently, employing [MnBr(CO)_5_] as the catalyst precursor afforded product **3a** in 84% yield in the presence of sodium acetate. The yield of **3a** could be further improved to 90% by increasing the temperature to 90 °C. Notably, the reaction occurred most efficiently in the absence of solvent and **3a** could be isolated in 92% yield. Further experiments showed that Mn(OAc)_2_·4H_2_O or Cp*Mn(CO)_3_ *in lieu* of [MnBr(CO)_5_] are ineffective. Additionally, manganese is essential for this transformation as in its absence no reaction occurred.

With the optimized reaction conditions in hand, the generality of this protocol was first tested by the reaction of indole heterocycles with **2aa** and the results are given in Scheme 2. Compared to the neat conditions, our studies showed that the product **3a** could be isolated in higher *E*/*Z* ratios when diethyl ether was used. Indoles substituted with reactive electrophilic functional groups which can undergo subsequent functionalization, such as the halides (–F, –Br, –I) and cyano substituents, were well tolerated. Introduction of a methyl or formyl group at the 3-position of the indole ring had no influence on the reaction efficiency (**3f–3h**), indicating that the reaction tolerates steric bulk in proximity of the reaction center of **1**. Moreover, this protocol was not restricted to indole substrates but also amenable to benzene- and thiophene-containing substrates, furnishing the corresponding products **3i–3k** in good yields. Furthermore, an *N*-pyrimidyl moiety could be employed as a directing group and the expected product **3l** was isolated in 79% yield. Notably, this reaction also exhibited high efficiency on a large scale. The desired product **3a** could be isolated in 91% yield (1.44 g) on a 6 mmol scale. In addition, this protocol was successfully applicable to 2-vinyloxiranes, as the coupling partner, and the products **3a** and **3g** were isolated in 54% and 69% yield, respectively.

**Scheme 2:**
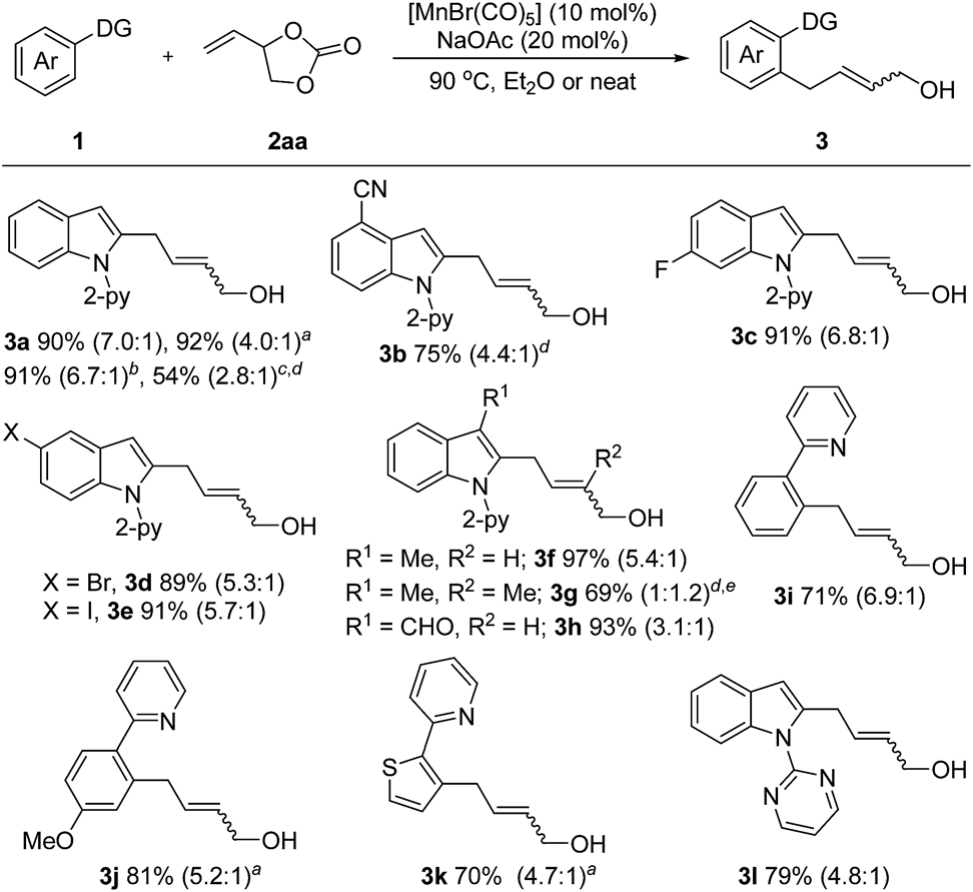
Unless otherwise specified, all reactions were carried out using **1** (0.2 mmol), **2** (0.3 mmol), [MnBr(CO)_5_] (10 mol%), and NaOAc (20 mol%) in Et_2_O (1.0 mL) at 90 °C under argon for 5 h, isolated yield, *E*/*Z* value given in parentheses. ^*a*^ Neat. ^*b*^ Reaction performed on a 6 mmol scale with a 47 h reaction time. ^*c*^ 2-Vinyloxirane (0.6 mmol) was used instead of **2aa**. ^*d*^ 10 h. ^*e*^ 2-Methyl-2-vinyloxirane (0.6 mmol) was used instead of **2aa**.

Encouraged by these inspiring results, we then pursued a more challenging successive C–H/C–C activation. Gratifyingly, fine tuning of the reaction conditions allowed the coupling of **1a** with dimethyl 2-vinylcyclopropane-1,1-dicarboxylate (**2ba**) to proceed smoothly under solvent free or concentrated DMF conditions (Scheme 3). Both electron-rich (–OMe) and electron deficient (–F, –Br, –I) *N*-(2-pyridyl)-indoles are amenable to this method, giving the corresponding products **4b–4e** in 52–90% yields. A 3-methyl substituent did not diminish the reactivity, as demonstrated by the formation of the desired products **4f** and **4g** in excellent yields. Moreover, the scope of the arene substrate could further be extended to phenylpyridine and thiophene derivatives, affording the corresponding products **4h–4j** in moderate yields.

**Scheme 3:**
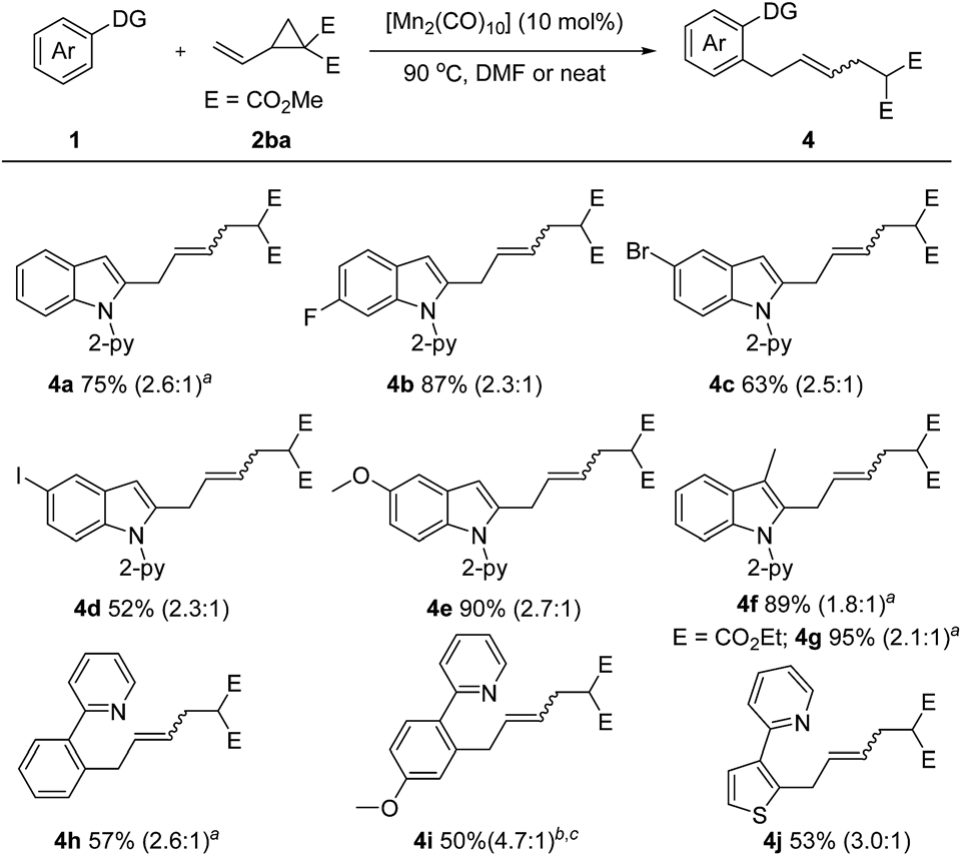
Unless otherwise specified, all reactions were carried out using **1** (0.2 mmol), **2** (0.3 mmol), and [Mn_2_(CO)_10_] (10 mol%) in DMF (0.2 mL) at 90 °C under argon for 24 h, isolated yield, *E*/*Z* value given in parentheses. ^*a*^ [MnBr(CO)_5_] (10 mol%) and NaOAc (20 mol%) were used under neat conditions. ^*b*^ Neat. ^*c*^ [Mn_2_(CO)_10_] (20 mol%).

Considering the importance of nitrogen-containing compounds and the ease of further transformation on this moiety, we next sought to apply our developed protocol to introduce a vicinal 2-arylated cyclopentenylamine unit. These are known to be key structures found within biologically active small molecules and are key intermediates in the synthesis of pharmaceutically important cyclopentane derivatives.^9*e*^ Pleasingly, when diazabicycle **2ca** was utilized, the desired aryl- and amine-substituted cyclopentenes **5a–5g** were obtained in 70–94% yields (Scheme 4). Not only *N*-(2-pyridyl)-indoles, but also phenylpyridines were suitable substrates. It is important to note that this reaction also proved to be efficient under neat conditions. Arguably, this is the first example of a first-row transition metal catalyzed C–H activation/six-membered ring scission sequence.

**Scheme 4:**
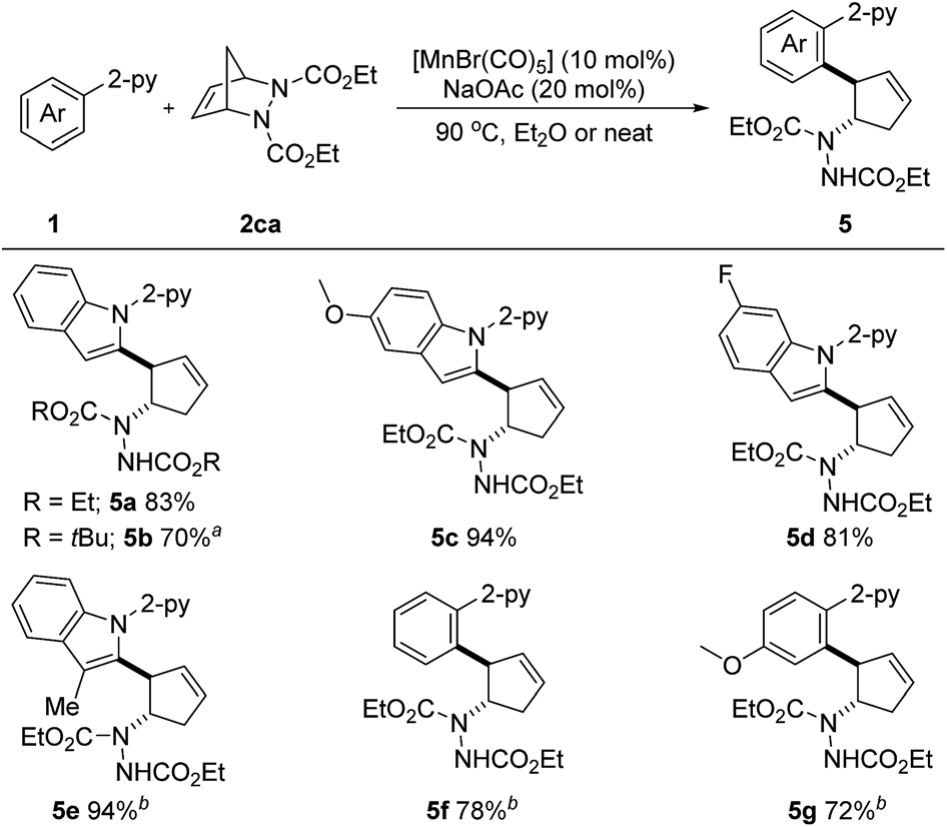
Unless otherwise specified, all reactions were carried out using **1** (0.2 mmol), **2** (0.3 mmol), [MnBr(CO)_5_] (10 mol%) and NaOAc (20 mol%) in Et_2_O (1.0 mL) at 90 °C under argon for 10 h, isolated yield. ^*a*^ 37 h. ^*b*^ [MnBr(CO)_5_] (20 mol%) and NaOAc (40 mol%) were used under neat conditions at 100 °C for 37 h.

Olefinic C–H activation could also be achieved under these conditions. For example, 2-(prop-1-en-2-yl)pyridine (**1m**) performed well in this transformation and the desired products **3m** and **5h**, a skipped diene with a valuable handle for further derivatization, were obtained in 75% and 62% yield respectively (Scheme 5).

**Scheme 5:**
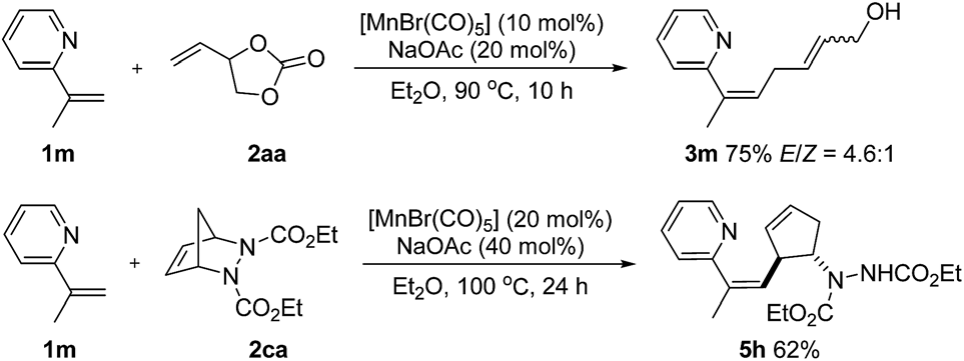
Manganese-catalyzed alkenyl C–H activation.

This transformation is presumed to proceed through an organometallic C–H activation process, as was supported by radical trapping experiments (for details, see Scheme S1 in the ESI†). The reaction of **1a** and **2aa** under standard conditions was found to be compatible with radical scavengers 2,4-di-*tert*-butyl-4-methylphenol (BHT) and 1,1-diphenylethylene.

To gain more insight into the reaction mechanism, H/D scrambling experiments were next conducted (for details, see the ESI†). No H/D exchange at the 2-position of **1a** was observed when **1a** was simply mixed with CD_3_OD and sodium acetate. Approximately 71% deuterium was incorporated into the 2-position of recovered **1a** when sodium acetate was replaced with [MnBr(CO)_5_]. Furthermore, a larger deuterium incorporation (85%) at the 2-position of **1a** was observed when **1a** was treated with CD_3_OD in the presence of both [MnBr(CO)_5_] and sodium acetate. These results suggest that the C–H activation step is reversible and might occur *via* a base-assisted cyclometalation process. In addition, a *k*_H_/*k*_D_ = 1.0 was observed from parallel reactions of **1a** and [D]-**1a** with **2aa**, respectively, which suggests that the cleavage of the C–H bond is not involved in the rate-determining step. Furthermore, when (3-pyridyl)-thiophene was utilized, the reaction occurred exclusively at the more electron rich 2-position (Scheme 6). On the basis of the above-mentioned results, we may draw the conclusion that the olefin coordination and insertion step is the rate-determining step, and that β-elimination is a facile process.^11^

**Scheme 6:**
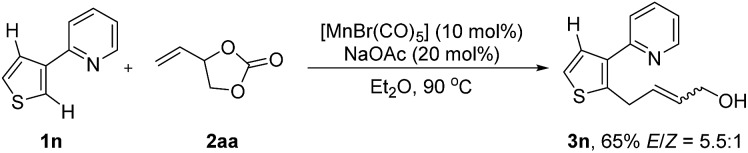
Intramolecular C–H competition experiment.

To acquire a better understanding of the observed reaction selectivity, a series of experiments were performed. As shown in Table 1, no obvious isomerization of the C=C bond was observed regardless of prolonged reaction time or decreased reaction temperature (entries 1–3, Table 1). On the contrary, solvent was found to have a dramatic effect on the *E*/*Z* selectivity (entries 3–5, Table 1). Compared to the neat reaction, DMF had a negative effect on the final *E*/*Z* selectivity, while diethyl ether had a positive effect. From these results, we inferred that the involvement of the π-allylmanganese intermediate in the reaction mechanism might be excluded and that the solvent imparts selectivity during this transformation.

**Table 1:** Exploration of the influencing factor for the reaction selectivity^*a*^

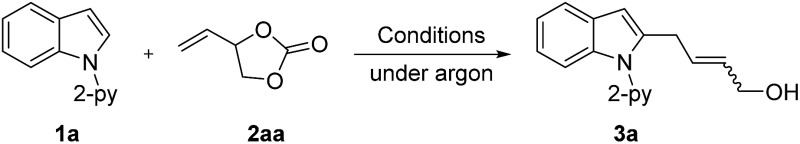					
Entry	Temp./°C	Time/h	Solvent	Yield	Ratio *E*/*Z*
1	90	5	Et_2_O	89	7.0
2	90	10	Et_2_O	89	6.9
3	60	10	Et_2_O	57	6.8
4	90	10	DMF	55	3.4
5	90	5	—	92	4.0

^*a*^All reactions were carried out using **1a** (0.2 mmol), **2aa** (0.3 mmol), [MnBr(CO)_5_] (10 mol%) and NaOAc (20 mol%) under different conditions, isolated yield, *E*/*Z* ratio is determined by ^1^H NMR.

## Conclusions

In conclusion, we have developed a general strategy to synthesize allylic alcohols, allylated arenes, functionalized cyclopentenes and skipped dienes, in which earth abundant manganese was utilized as the catalyst.^12^ This protocol represents a combination of C–H and C–C/C–Het bond activation. Both aromatic and olefinic substrates are functionalized in this reaction. This work broadens the scope of manganese catalysis to include a series of new coupling partners. Additionally, this reaction can occur efficiently under neat conditions yet does not require the use of a large excess of a coupling partner as the solvent, which is unprecedented in abundant metal catalysis. Finally, β-carbon and β-nitrogen elimination were shown to be feasible under low-valent manganese catalysis for the first time.
