# Supplementary material for: Manganese-catalyzed allylation via sequential C–H and C–C/C–Het bond activation
Source: Chem Sci. 2017 Feb 24;8(5):3379–83. doi: 10.1039/c7sc00230k (PMC5416941; doi:10.1039/c7sc00230k)
Supplement: Supplementary file 1 [file SC-008-C7SC00230K-s001.pdf]

Electronic Supplementary Material (ESI) for Chemical Science.
This journal is © The Royal Society of Chemistry 2017

Manganese-Catalyzed Allylation via Sequential C–H and C–C/C–Het Bond Activation

Qingquan Lu, Felix J. R. Klauck and Frank Glorius*

Organisch-Chemisches Institut, Westfälische Wilhelms-Universität Münster, Corrensstraße 40, 48149 Münster, Germany

glorius@uni-muenster.de

Supporting Information

Table of Contents

General Information……………………………………………………………...S3

Experimental Section……………………………………………………………..S4

1) Optimization of the reaction conditions……………………………………….S4

2) Procedure and analytical data of compounds **3-5**…………………………….S4

3) Gram-scale synthesis of **1a**……………………………………………………S20

4) Radical trapping experiments……………………………………………….....S20

5) H/D scrambling experiments…………………………………………………..S21

6) KIE experiments……………………………………………………………….S24

7) Proposed mechanism…………………………………………………………..S24

References…………………………………………………………………………S25

NMR Spectra of Products…………………………………………………………S26

General information

Unless otherwise noted, all reactions were carried out under an atmosphere of argon in oven-dried glassware cooled down under vacuum. Reaction temperatures are reported as the temperature of the heat transfer medium surrounding the vessel unless otherwise stated. Anhydrous solvents were purchased from ROTH and stored over molecular sieves under argon. Commercially available chemicals were obtained from Acros Organics, Aldrich Chemical Co., Alfa Aesar, ABCR, TCI Europe and used as received unless otherwise stated. *N*-2-pyridylindoles,[1] *N*-2-pyrimidylindoles,[2] 2-arylpyridines,[3] 2-(prop-1-en-2-yl)pyridine,[4] dimethyl 2-vinylcyclopropane-1,1-dicarboxylate,[5] and meso-2,3-diazabicyclo[2.2.1]hept-5-ene-*N,N'*-diethyldicarboxylate[6] were prepared following literature procedures. Analytical thin layer chromatography was performed on Polygram SIL G/UV254 plates. TLC plates were visualized by exposure to short wave ultraviolet light (254 nm, 366 nm). Flash chromatography was performed on Merck silica gel (40-63 mesh) by standard techniques using appropriate mixtures of *n*-pentane and ethyl acetate. ^{1}H and ^{13}C NMR spectra were recorded on a Bruker AV 300 or AV 400, Varian 500 MHz INOVA or Varian Unity plus 600 in solvents as indicated. Chemical shifts (δ) for ^{1}H and ^{13}C NMR spectra are given in ppm relative to TMS. The residual solvent signals were used as references for ^{1}H and ^{13}C NMR spectra and the chemical shifts converted to the TMS scale (TMS: δH = 0.00 ppm, $CDCl_3$: δH = 7.26 ppm, δC = 77.16 ppm; CD_3OD: δH = 3.31 ppm, δC = 49.00 ppm; DMSO-d_6: δH = 2.50 ppm;). Exact ESI mass spectra were recorded on a Bruker Daltonics MicroTof. Mass Calibration was carried out directly before the measurement of the sample using clusters of sodium formate. Infrared spectra were recorded neat on a Shimadzu FTIR-8400S. The wave numbers (υ) of recorded IR-signals are quoted in cm^{-1}.

Experimental section

1) Optimization of the reaction conditions.

Table S1 | Optimization of the reaction conditions.[a]

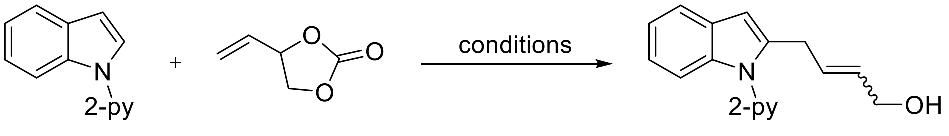

Entry	Cat. (10 mol%)	Additive (20 mol%)	Temp. / ºC	Solvent	Yield of **3a** (%)
1	[$Mn_2(CO)_{10}$]	-	80	Et_2O	41
2	[$Mn_2(CO)_{10}$]	-	80	THF	17
3	[$Mn_2(CO)_{10}$]	-	80	Hexane	26
4	[$Mn_2(CO)_{10}$]	-	80	DMF	79
5	[$MnBr(CO)_5$]	-	80	Et_2O	13
6	[$MnBr(CO)_5$]	Cy_2NH	80	Et_2O	72
7	[$MnBr(CO)_5$]	NaOAc	80	Et_2O	84
8	[$MnBr(CO)_5$]	NaOAc	80	DMF	55
9[b]	[$MnBr(CO)_5$]	NaOAc	90	Et_2O	90
10[b]	[$MnBr(CO)_5$]	NaOAc	90	neat	92
11[b]	[$Mn_2(CO)_{10}$]	NaOAc	90	neat	90
12	Cp*$Mn(CO)_3$	NaOAc	90	Et_2O	0
13	$Mn(OAc)_2$.$4H_2O$	NaOAc	90	Et_2O	0
14	-	NaOAc	90	Et_2O	0

[a] Unless otherwise specified, all reactions were carried out using **1a** (0.2 mmol), **2aa** (0.3 mmol), catalyst precursor (10 mol%), additive (20 mol%) in solvent (1.0 mL) for 10 h, isolated yields. [b] 5 h.

2) Procedure and analytical data of compounds 3-5.

2.1) Procedure and analytical data of compounds 3.

General procedure: In a 10 mL dry Schlenk tube with a stirring bar, *N*-2-pyridylindole (**1**) (0.20 mmol), [$MnBr(CO)_5$] (10 mol%, 5.5 mg) and NaOAc (20 mol%, 3.3 mg) were added under air. Then the reaction vessel was evacuated and filled with argon for three times; Afterwards, vinyl-1,3-dioxolan-2-one (**2aa**) (0.30 mmol) and Et_2O (1 mL, anhydrous, no solvent was added under neat condition) were added under an argon atmosphere. The tube was sealed and the mixture was stirred at 90 °C for the determined time. Then, the volatiles were removed and the

analytically pure product was obtained by flash chromatography (silica; gradient of pentane/EtOAc).

(*E*/*Z*)-4-(1-(pyridin-2-yl)-1*H*-indol-2-yl)but-2-en-1-ol[7]

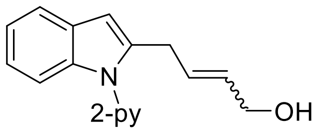

Following the general procedure, product **3a** was isolated in 89% yield. **^{1}H NMR** (300 MHz, $CDCl_3$) δ 8.55 (ddd, *J* = 4.9, 1.9, 0.8 Hz, 1H), 7.85 – 7.71 (m, 1H), 7.52 – 7.44 (m, 1H), 7.35 – 7.30 (m, 1H), 7.27 – 7.19 (m, 2H), 7.09 – 6.94 (m, 2H), 6.51 – 6.20 (m, 1H), 5.71 – 5.23 (m, 2H), 3.99 (d, *J* = 5.1 Hz, 0.25 H), 3.86 (dd, *J* = 5.7, 1.1 Hz, 1.75 H), 3.62 – 3.45 (m, 2H), 1.96 (br, 1H). **^{13}C NMR** (75 MHz, $CDCl_3$) δ *Major isomer:* 151.40, 149.63, 139.04, 138.44, 137.42, 131.28, 128.65, 128.56, 122.25, 122.01, 121.24, 120.84, 120.19, 110.20, 103.29, 63.26, 30.72. *Minor isomer:* 151.34, 149.70, 139.34, 138.57, 130.42, 128.40, 122.03, 120.89, 110.14, 103.02, 58.33, 26.22. *Five peaks are missing probably due to overlap*. **HRMS**: m/z (ESI) calcd for $C_{17}H_{16}N_2NaO$ $[M+Na]^+$ 287,1160, found 287,1155.

(*E*/*Z*)-2-(4-hydroxybut-2-en-1-yl)-1-(pyridin-2-yl)-1*H*-indole-4-carbonitrile

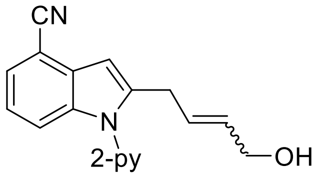

Following the general procedure, product **3b** was isolated in 75% yield. **^{1}H NMR** (300 MHz, $CDCl_3$) δ 8.73 – 8.42 (m, 1H), 7.97 – 7.67 (m, 1H), 7.49 – 7.24 (m, 4H), 7.15 – 6.96 (m, 1H), 6.67 – 6.49 (m, 1H), 5.76 – 5.34 (m, 2H), 4.04 (d, *J* = 6.0 Hz, 0.37 H), 3.95 (dd, *J* = 5.6, 1.3 Hz, 1.63 H), 3.59 (d, *J* = 6.7 Hz, 0.38 H), 3.54 (d, *J* = 6.5 Hz, 1.63 H), 1.68 (br, 1H). **^{13}C NMR** (75 MHz, $CDCl_3$) δ *Major isomer:* ^{13}C NMR (75 MHz, $CDCl_3$) δ 150.36, 149.99, 142.40, 138.86, 137.17, 132.27, 130.14, 127.29, 125.89, 123.20, 121.68, 121.40, 118.83, 115.02, 102.36, 101.87, 63.17, 30.71. *Minor isomer:* ^{13}C NMR (75 MHz, $CDCl_3$) δ 150.31, 150.07, 142.61, 138.98, 131.24, 125.93, 123.22, 121.72, 118.79, 114.93, 102.41, 101.63, 58.38, 26.25. *Four peaks are missing probably due to overlap*. **HRMS**: m/z (ESI) calcd for $C_{18}H_{15}N_3NaO$ $[M+Na]^+$ 312,1113, found 312,1107.

(*E*/*Z*)-4-(6-fluoro-1-(pyridin-2-yl)-1*H*-indol-2-yl)but-2-en-1-ol

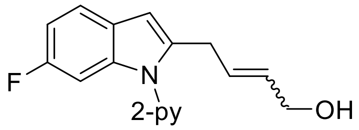

Following the general procedure, product **3c** was isolated in 91% yield. **^{1}H NMR (400 MHz, $CDCl_3$)** δ = 8.71 – 8.61 (ddd, *J* = 4.8, 2.0, 0.8 Hz, 1H), 7.94 – 7.85 (m, 1H), 7.46 (dd, *J* = 8.6,

5.4 Hz, 1H), 7.43 – 7-37 (m, 1H), 7.37 – 7.31 (m, 1H), 7.07 – 6.99 (m, 1H), 6.89 (ddd, *J* = 9.5, 8.6, 2.3 Hz, 1H), 6.44 – 6.38 (m, 1H), 5.79 – 5.44 (m, 2H), 4.10 (d, *J* = 5.4 Hz, 0.26H), 3.98 (dd, *J* = 5.7, 1.3 Hz, 1.74H), 3.62 (d, *J* = 5.9 Hz, 0.27H), 3.59 (d, *J* = 6.6 Hz, 1.74H), 1.85 (br, 1H); **^{13}C NMR (101 MHz, $CDCl_3$)** *Major isomer:* δ = 159.98 (d, *J* = 237.5 Hz), 151.09 , 149.74 , 139.43 (d, *J* = 3.8 Hz), 138.65 , 137.45 (d, *J* = 12.0 Hz), 131.44 , 128.38 , 124.90 , 122.54 , 121.01 , 120.77 (d, *J* = 9.9 Hz), 109.23 (d, *J* = 24.2 Hz), 103.14 , 97.19 (d, *J* = 27.2 Hz), 63.19 , 30.70; *Minor isomer:* δ = 151.03, 149.83, 138.78, 130.57, 128.15, 124.89, 120.80 (d, *J* = 9.9 Hz), 109.27 (d, *J* = 24.0 Hz), 102.85, 97.13 (d, *J* = 27.3 Hz), 58.31, 26.22. *Two peaks are missing probably due to overlap, three peaks probably have too low intensities due to C-F coupling.* **^{19}F NMR (282 MHz, $CDCl_3$)** *Major isomer:* δ = -120.86 (td, *J* = 9.8, 5.4 Hz); *Minor isomer:* δ = -120.79; **HRMS** m/z (ESI): calcd. for $C_{17}H_{15}N_2OFNa$ 305.1061, found 305.1061; **ATR-IR** ν (cm^{-1}) 3333, 3082, 3063, 3013, 2905, 2862, 2357, 1616, 1586, 1570, 1559, 1470, 1439, 1385, 1354, 1296, 1273, 1207, 1180, 1138, 1096, 1049, 972, 937, 833, 810, 779, 679, 656.

(*E*/*Z*)-4-(5-Bromo-1-(pyridin-2-yl)-1*H*-indol-2-yl)but-2-en-1-ol

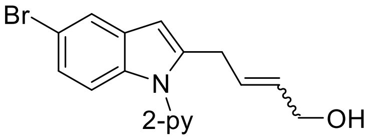

Following the general procedure, product **3d** was isolated in 89% yield. **^{1}H NMR (400 MHz, $CDCl_3$)** δ = 8.54 – 8.59 (m, 1H), 7.87 – 7.77 (m, 1H), 7.63 – 7.57 (m, 1H), 7.35 – 7.29 (m, 1H), 7.27 (ddd, *J* = 7.5, 4.9, 1.0 Hz, 1H), 7.15 – 7.07 (m, 2H), 6.33 – 6.28 (m, 1H), 5.69 – 5.36 (m, 2H), 4.01 (d, *J* = 5.7 Hz, 0.33H), 3.90 (dd, *J* = 5.7, 1.3 Hz, 1.76H), 3.55 (d, *J* = 6.7 Hz, 0.34H), 3.52(d, *J* = 6.6 Hz, 1.76H), 1.56 (br, 1H); **^{13}C NMR (101 MHz, $CDCl_3$)** *Major isomer:* δ = 151.01, 149.80, 140.38, 138.62, 136.15, 131.61, 130.26, 128.21, 124.76, 122.69, 122.62, 121.17, 113.97, 111.75, 102.69, 63.28, 30.71; *Minor isomer:* δ = 150.95, 149.87, 140.66, 138.76, 130.69, 130.28, 128.06, 124.79, 122.72, 122.63, 121.19, 114.02, 111.68, 102.41, 58.40, 26.23. *One peak is missing probably due to overlap*. **HRMS** m/z (ESI): calcd. for $C_{17}H_{15}N_2OBrNa$ 365.0260, found 365.0269; **ATR-IR** ν (cm^{-1}) 3310, 3013, 2859, 1586, 1574, 1470, 1439, 1381, 1339, 1273, 1207, 1150, 1092, 1049, 972, 864, 779, 745, 733.

(*E*/*Z*)-4-(5-iodo-1-(pyridin-2-yl)-1*H*-indol-2-yl)but-2-en-1-ol

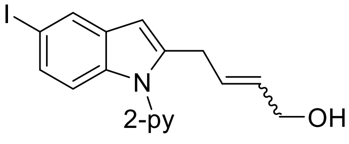

Following the general procedure, product **3e** was isolated in 91% yield. **^{1}H NMR (300 MHz, $CDCl_3$)** δ 8.60 – 8.48 (m, 1H),

7.89 – 7.72 (m, 2H), 7.33 – 7.21 (m, 3H), 7.02 – 6.93 (m, 1H), 6.31 – 6.21 (m, 1H), 5.70 – 5.14 (m, 2H), 3.97 (d, *J* = 5.5 Hz, 0.3 H), 3.86 (dd, *J* = 5.6, 1.2 Hz, 1.7 H), 3.63 – 3.35 (m, 2H), 2.39 (br, 1H); **^{13}C NMR (75 MHz, CDCl$_3$)** *Major isomer:* δ 150.87, 149.73, 140.04, 138.65, 136.61, 131.63, 131.00, 130.24, 128.90, 128.01, 122.65, 121.20, 112.25, 102.35, 84.32, 63.13, 30.59; *Minor isomer:* ^{13}C NMR (75 MHz, CDCl$_3$) δ 150.81, 149.81, 140.31, 138.78, 130.74, 130.27, 128.93, 127.84, 112.18, 102.07, 84.36, 58.28, 26.12. *Four peaks are missing probably due to overlap*. **HRMS** m/z (ESI): calcd. for $C_{17}H_{15}IN_2NaO$ 413,0127, found 413,0121.

(*E*/*Z*)-4-(3-methyl-1-(pyridin-2-yl)-1*H*-indol-2-yl)but-2-en-1-ol

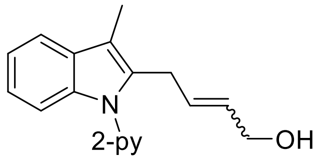

Following the general procedure, product **3f** was isolated in 97% yield. **^{1}H NMR (300 MHz, CDCl$_3$)** δ 8.58 – 8-49 (m, 1H), 7.82 – 7.77 (m, 1H), 7.52 – 7.42 (m, 1H), 7.36 – 7.28 (m, 1H), 7.27 – 7.18 (m, 2H), 7.12 – 7.01 (m, 2H), 5.56 – 5.15 (m, 2H), 3.88 (d, *J* = 5.7 Hz, 0.31H), 3.78 (dd, *J* = 5.8, 1.3 Hz, 1.68H), 3.60 (d, *J* = 5.9 Hz, 0.32H), 3.55 (dd, *J* = 6.1, 1.4 Hz, 1.69H), 2.25 (s, 0.47H), 2.23 (s, 2.48H), 1.45 (br, 1H); **^{13}C NMR (75 MHz, CDCl$_3$)** *Major isomer:* δ = 151.76, 149.56, 138.33, 136.65, 133.89, 130.24, 129.43, 129.05, 122.18, 121.93, 121.19, 120.38, 118.48, 110.82, 110.08, 63.28, 27.91, 8.89; *Minor isomer:* δ = 149.64, 138.50, 134.59, 129.48, 129.15, 122.21, 121.32, 120.46, 110.48, 109.98, 58.23, 23.93, 8.91. *Five peaks are missing due to overlap*. **HRMS** m/z (ESI): calcd. for $C_{18}H_{18}N_2OH$ 279.1492, found 279.1494; **ATR-IR** ν (cm^{-1}) 3310, 3013, 2916, 2859, 1586, 1566, 1470, 1458, 1435, 1362, 1316, 1285, 1223, 1150, 1088, 995, 968, 779, 737.

(*E*/*Z*)-2-methyl-4-(3-methyl-1-(pyridin-2-yl)-1H-indol-2-yl)but-2-en-1-ol

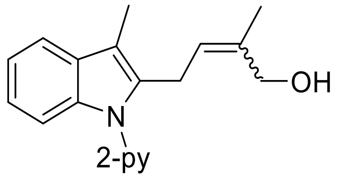

Following the general procedure, 2-methyl-2-vinyloxirane (0.6 mmol) was used instead of **2aa**, product **3g** was isolated in 69% yield. **^{1}H NMR (300 MHz, CDCl$_3$)** ^{1}H NMR δ 8.49 (t, *J* = 4.3 Hz, 1H), 7.77 – 7.65 (m, 1H), 7.49 – 7.38 (m, 1H), 7.25 (t, *J* = 7.4 Hz, 1H), 7.21 – 7.10 (m, 2H), 7.09 – 6.98 (m, 2H), 5.11 (t, *J* = 6.7 Hz, 0.5H), 5.02 (t, *J* = 6.9 Hz, 0.6H), 3.77 (s, 1.1H), 3.62 (s, 0.9H), 3.57 – 3.43 (m, 2H), 2.22 (s, 3H), 2.07 (s, 1H), 1.49 (s, 1.8H), 1.34 (s, 1.3H); **^{13}C NMR (75 MHz, CDCl$_3$)** *Major isomer:* δ 151.73, 149.51, 138.41, 136.60, 135.27, 135.25, 129.48, 124.34, 122.02, 121.90, 121.38, 120.36, 118.34, 110.07, 109.93, 61.05, 23.87, 21.21, 8.87, *Two peaks are missing due to overlap*; *Minor isomer:* δ 149.58, 138.39, 136.70, 135.48, 129.42, 122.62, 121.97, 121.41,

120.28, 110.03, 109.87, 68.28, 23.94, 13.55, 8.88; *Four peaks are missing due to overlap*; **HRMS**: m/z (ESI) calcd for $C_{19}H_{20}N_2NaO$ $[M+Na]^+$ 315,1473, found 315,1468.

Determination of structure was achieved with TOCSY and NOE-Experiments

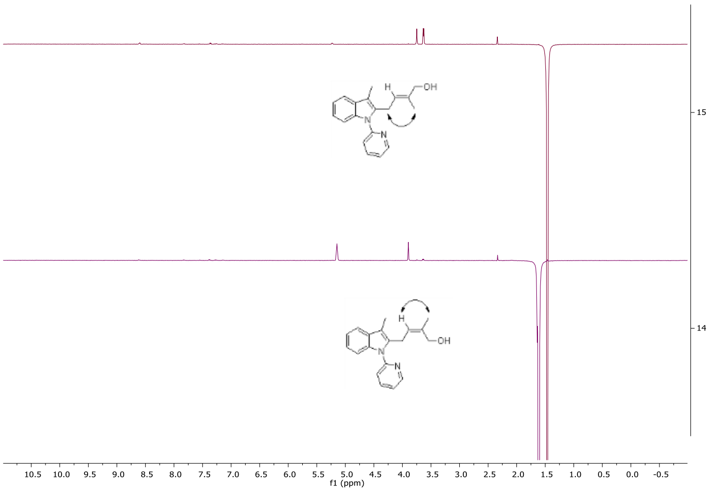

(*E*/*Z*)-2-(4-hydroxybut-2-en-1-yl)-1-(pyridin-2-yl)-1*H*-indole-3-carbaldehyde

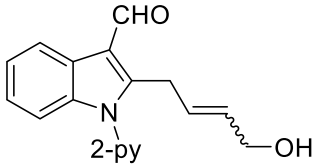

Following the general procedure, product **3h** was isolated in 93% yield. **^{1}H NMR (400 MHz, $CDCl_3$)** δ 10.22 (s, 0.24 H), 10.19 (s, 0.78 H), 8.67 – 8.57 (m, 1H), 8.24 (dt, *J* = 6.8, 0.9 Hz, 0.76H), 8.19 (dt, *J* = 6.8, 0.9 Hz, 0.24 H), 7.95 – 7.82 (m, 1H), 7.42 – 7.32 (m, 2H), 7.26 – 7.06 (m, 3H), 5.61 – 5.41 (m, 1H), 5.38 – 5.20 (m, 1H), 3.94 – 3.85 (m, 1H), 3.85 – 3.73 (m, 3H), 2.08 (s, 1H); **^{13}C NMR (101 MHz, $CDCl_3$)** *Major isomer:* δ 185.32, 150.14, 149.70, 148.83, 139.01, 137.24, 132.12, 126.93, 125.68, 124.14, 124.02, 123.64, 122.20, 121.34, 115.70, 110.61, 62.79, 27.73; *Minor isomer:* δ 150.27, 149.65, 148.86, 139.14, 137.15, 130.74, 126.86, 126.06, 124.09, 122.26, 120.72, 115.30, 110.67, 58.09, 24.09. *Three peaks are missing due to overlap*. **HRMS** m/z (ESI): calcd. for $C_{18}H_{16}N_2NaO_2$ 315,1109, found 315,1104.

(*E*/*Z*)-4-(2-(pyridin-2-yl)phenyl)but-2-en-1-ol[7]

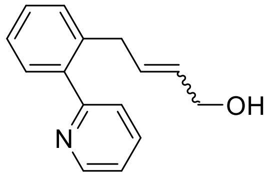

Following the general procedure, product **3i** was isolated in 71% yield. **^{1}H NMR (400 MHz, $CDCl_3$)** δ 8.64 – 8.47 (m, 1H), 7.73 – 7.58 (m, 1H), 7.36 – 7.10 (m, 6H), 5.66 – 5.55 (m, 0.9 H), 5.53 – 5.46 (m, 0.3 H), 5.38 – 5.29 (m, 0.9 H), 3.93 (d, *J* = 5.3 Hz, 0.3 H), 3.87 (dd, *J* = 5.9, 1.3 Hz, 1.8 H), 3.46 – 3.42 (m, 0.3 H), 3.38 (d, *J* = 6.6 Hz, 1.8 H), 1.91 (br, 1H); **^{13}C NMR (101 MHz, $CDCl_3$)** *Major isomer:* δ 159.93, 149.17, 140.43, 137.89, 136.37, 131.51, 130.29, 130.16, 129.96,

128.63, 126.47, 124.30, 121.94, 63.45, 36.08; *Minor isomer:* δ 160.07, 149.07, 140.17, 138.38, 136.65, 131.35, 130.09, 129.85, 129.20, 128.78, 126.40, 124.49, 121.98, 58.14, 31.22.

(*E*/*Z*)-4-(5-methoxy-2-(pyridin-2-yl)phenyl)but-2-en-1-ol

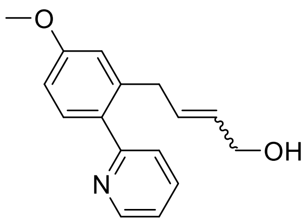

Following the general procedure, product **3j** was isolated in 81% yield. **^{1}H NMR (300 MHz, CDCl$_3$)** δ 8.65 – 8.50 (m, 1H), 7.70 – 7.57 (m, 1H), 7.29 – 7.04 (m, 3H), 6.82 – 6.69 (m, 2H), 5.68 – 5.55 (m, 0.87 H), 5.55 – 5.45 (m, 0.32 H), 5.43 – 5.26 (m, 0.85 H), 3.94 (d, *J* = 3.8 Hz, 0.34 H), 3.88 (d, *J* = 5.2 Hz, 1.73 H), 3.75 (s, 3H), 3.44 (d, *J* = 5.3 Hz, 0.32 H), 3.38 (d, *J* = 6.5 Hz, 1.69 H), 2.02 (br, 1H); **^{13}C NMR (75 MHz, CDCl$_3$)** *Major isomer:* δ 159.74, 159.64, 149.10, 139.53, 133.20, 133.20, 131.30, 131.25, 130.47, 124.32, 121.58, 115.76, 111.56, 63.40, 55.39, 36.28; *Minor isomer:* δ ^{13}C NMR (75 MHz, CDCl$_3$) δ 159.85, 159.80, 148.99, 140.02, 136.60, 131.43, 131.15, 129.43, 124.51, 121.61, 115.42, 111.56, 58.08, 31.37. *Two peaks are missing due to overlap*. **HRMS** m/z (ESI): calcd. for $C_{16}H_{17}NNaO_2$ 278,1157, found 278,1151;

(*E*/*Z*)- 4-(2-(pyridin-2-yl)thiophen-3-yl)but-2-en-1-ol[7]

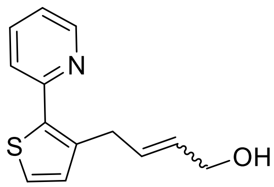

Following the general procedure, product **3k** was isolated in 70% yield. **^{1}H NMR (300 MHz, CDCl$_3$)** δ 8.71 – 8.29 (m, 1H), 7.68 – 7.56 m, 1H), 7.49 – 7.37 (m, 1H), 7.27 – 7.20 (m, 1H), 7.15 – 6.99 (m, 1H), 6.92 (d, *J* = 5.1 Hz, 0.18 H), 6.88 (d, *J* = 5.1 Hz, 0.82 H), 5.91 – 5.76 (m, 0.83 H), 5.71 – 5.65 (m, 0.34 H), 5.65 – 5.51 (m, 0.83 H), 4.18 (d, *J* = 5.3 Hz, 0.36 H), 4.09 – 3.95 (m, 1.70 H), 3.67 (d, *J* = 5.6 Hz, 0.37 H), 3.63 – 3.50 (m, 1.71 H), 1.70 (br, 1H); **^{13}C NMR (75 MHz, CDCl$_3$)** *Major isomer:* δ 153.20, 149.72, 138.51, 137.54, 136.66, 131.02, 130.66, 130.48, 126.00, 122.01, 121.72, 63.49, 32.34; *Minor isomer:* δ ^{13}C NMR (75 MHz, CDCl$_3$) δ 153.34, 149.54, 138.43, 136.90, 130.87, 130.64, 129.37, 125.91, 122.82, 121.82, 58.20, 27.81. *One peak is missing due to overlap*.

(*E*/*Z*)- 4-(1-(pyrimidin-2-yl)-1*H*-indol-2-yl)but-2-en-1-ol[7]

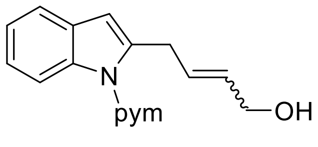

Following the general procedure, product **3l** was isolated in 79% yield. **^{1}H NMR (300 MHz, CDCl$_3$)** δ 8.68 (d, *J* = 4.8 Hz, 2H), 8.21 – 8.09 (m, 1H), 7.50 – 7.39 (m, 1H), 7.19 – 7.06 (m, 2H), 7.04 (t, *J*

= 4.8 Hz, 1H), 6.44 – 6.34 (m, 1H), 5.84 – 5.48 (m, 2H), 4.15 (d, *J* = 5.3 Hz, 0.39 H), 3.93 (dd, *J* = 5.7, 0.87 Hz, 1.69 H), 3.90 – 3.81 (m, 2.08 H), 1.48 (br, 1H); **^{13}C NMR (75 MHz, $CDCl_3$)** *Major isomer:* δ 158.25, 139.73, 137.13, 131.12, 129.61, 129.25, 122.84, 121.96, 119.95, 117.27, 113.81, 106.56, 63.47, 32.57. *Two peaks are missing due to overlap*. *Minor isomer:* δ 158.20, 139.79, 137.16, 130.29, 129.18, 122.89, 122.02, 117.23, 113.97, 106.40, 58.67, 28.23; *Four peaks are missing due to overlap*.

2-(*E*/*Z*)-5-(*Z*)-6-(pyridin-2-yl)hepta-2,5-dien-1-ol

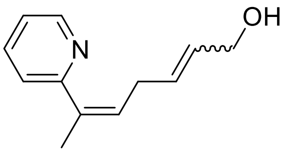

Following the general procedure, product **3m** was isolated in 75% yield. **^{1}H NMR (400 MHz, $CDCl_3$)** δ 8.52 (d, *J* = 3.9 Hz, 1H), 7.66 – 7.52 (m, 1H), 7.18 – 6.98 (m, 2H), 5.74 – 5.43 (m, 3H), 4.13 – 3.90 (m, 2H), 2.81 (t, *J* = 7.5 Hz, 0.34H), 2.74 (t, *J* = 3.94 Hz, 1,66H), 2.38 (br, 1H), 2.05 (s, 2.35H), 2.02 (s, 0.48H); **^{13}C NMR (101 MHz, $CDCl_3$)** *Major isomer:* δ 159.69, 149.26, 136.82, 136.16, 130.97, 129.78, 127.27, 123.13, 121.76, 63.53, 31.91, 23.90; *Minor isomer:* δ ^{13}C NMR (101 MHz, $CDCl_3$) δ 159.74, 149.00, 136.50, 135.58, 130.63, 129.21, 127.92, 121.88, 57.75, 27.80, 24.09. *One peak is missing due to overlap*. **HRMS** m/z (ESI): calcd. for $C_{12}H_{15}NNaO$ 212,1051, found 212,1046.

Determination of structure was achieved with TOCSY and NOE-Experiments

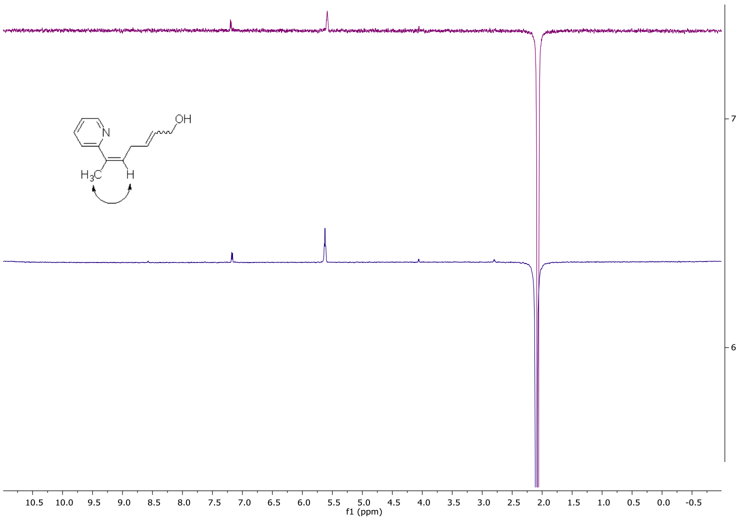

(*E*/*Z*)-4-(3-(pyridin-2-yl)thiophen-2-yl)but-2-en-1-ol

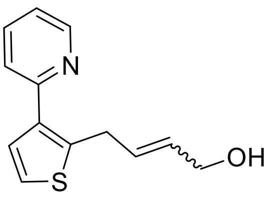

Following the general procedure, product **3n** was isolated in 65% yield. **^{1}H NMR (300 MHz, $CDCl_3$)** δ = 8.58 (d, *J* = 4.0 Hz, 1H), 7.70 – 7.57

(m, 1H), 7.42 – 7.33 (m, 1H), 7.21 (t, *J* = 5.8 Hz, 1H), 7.16 – 7.08 (m, 2H), 5.90 – 5.59 (m, 2H), 4.16 (s, 0.31H), 4.02 (s, 1.69H), 3.87 (d, *J* = 6.1 Hz, 0.30H), 3.79 (d, *J* = 6.2 Hz, 1.69H), 1.59 (s, 1H); **^{13}C NMR (101 MHz, $CDCl_3$)** *Major isomer:* δ = 155.15, 149.53, 141.28, 137.39, 136.52, 131.06, 130.65, 128.88, 122.89, 122.80, 121.53, 63.42, 31.79; *Minor isomer:* δ = 155.20, 149.32, 141.95, 136.83, 136.79, 130.97, 129.90, 128.76, 123.20, 122.85, 121.63, 58.12, 27.21; **HRMS** m/z (ESI): calcd. for $C_{13}H_{13}NOSNa$ 254.0610, found 254.0613; **ATR-IR** ν (cm^{-1}) 3275, 3063, 3013, 2893, 2855, 1586, 1566, 1535, 1466, 1439, 1420, 1373, 1339, 1277, 1231, 1150, 1092, 991, 968, 945, 856, 791, 741, 718, 679, 648.

2.2) Procedure and analytical data of compounds 4.

General procedure: In a 10 mL dry Schlenk tube with a stirring bar, *N*-2-pyridylindole (**1**) (0.20 mmol), [$Mn_2(CO)_{10}$] (10 mol%, 7.8 mg) were added under air. Then the reaction vessel was evacuated and filled with argon for three times; Afterwards, 2-vinylcyclopropane-1,1-dicarboxylate (**2ba**) (0.30 mmol) and DMF (0.2 mL, anhydrous, no solvent was added under neat condition) were added under an argon atmosphere. The tube was sealed and the mixture was stirred at 90 °C for the determined time. Then, the volatiles were removed and the analytically pure product was obtained by flash chromatography (silica; gradient of pentane/EtOAc).

(*E*/*Z*)-dimethyl 2-(4-(1-(pyridin-2-yl)-1*H*-indol-2-yl)but-2-en-1-yl)malonate[8]

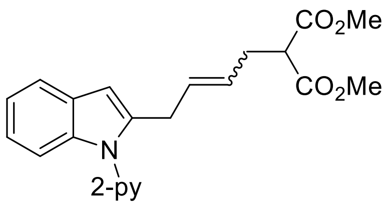

Following the general procedure, [$MnBr(CO)_5$] (10 mol%) and NaOAc (20 mol%) were used to replace $Mn_2(CO)_{10}$, product **4a** was isolated in 75% yield. **^{1}H NMR (400 MHz, $CDCl_3$)** δ 8.69 – 8.43 (m, 1H), 7.85 – 7.73 (m, 1H), 7.53 – 7.46 (m, 1H), 7.37 (d, *J* = 8.0 Hz, 0.35 H), 7.34 (d, *J* = 8.0 Hz, 0.73 H), 7.28 – 7.20 (m, 2H), 7.11 – 7.00 (m, 2H), 6.35 (s, 0.25 H), 6.33 (s, 0.66 H), 5.62 – 5.49 (m, 1H), 5.38 – 5.20 (m, 1H), 3.62 (s, 6H), 3.57 (d, *J* = 7.3 Hz, 0.62 H), 3.48 (d, *J* = 6.7 Hz, 1.4 H), 3.32 – 3.22 (m, 1H), 2.54 (t, *J* = 8.0 Hz, 0.55 H), 2.46 (t, *J* = 7.7 Hz, 1.42 H); **^{13}C NMR (101 MHz, $CDCl_3$)** *Major isomer:* δ 169.37, 151.42, 149.64, 139.39, 138.35, 137.37, 129.86, 128.61, 127.75, 122.12, 121.92, 121.12, 120.78, 120.14, 110.20, 103.08, 52.60, 51.75, 31.79, 31.06. *Two peaks are missing due to overlap. Minor isomer:* δ 169.39, 149.79, 139.43, 138.45, 137.42, 129.08, 126.49, 122.14, 102.91, 52.66, 51.63,

26.79, 26.05. *Nine peaks are missing due to overlap.*

(*E*/*Z*)-dimethyl 2-(4-(6-fluoro-1-(pyridin-2-yl)-1*H*-indol-2-yl)but-2-en-1-yl)malonate

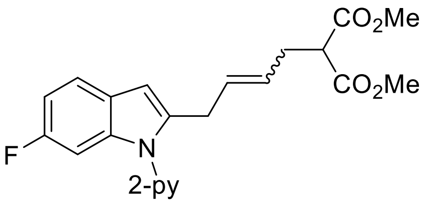

Following the general procedure, product **4b** was isolated in 87% yield. **^{1}H NMR (400 MHz, CDCl$_3$)** δ 8.70 – 8.60 (m, 1H), 7.96 – 7.83 (m, 1H), 7.50 – 7.29 (m, 3H), 7.04 (dd, *J* = 10.1, 2.3 Hz, 1H), 6.88 (td, *J* = 9.1, 2.4 Hz, 1H), 6.39 (s, 0.31H), 6.37 (s, 0.66H), 5.67 – 5.54 (m, 1H), 5.46 – 5.27 (m, 1H), 3.70 (s, 6H), 3.61 (d, *J* = 7.3 Hz, 0.67H), 3.52 (d, *J* = 6.6 Hz, 1.45H), 3.40 – 3.28 (m, 1H), 2.61 (t, *J* = 7.6 Hz, 0.61H), 2.54 (t, *J* = 7.3 Hz, 1.39H). **^{13}C NMR (101 MHz, CDCl$_3$)** *Major isomer* δ 169.35, 159.96 (d, *J* = 237.2 Hz), 151.11, 149.77, 139.76 (d, *J* = 3.9 Hz), 138.57, 137.38 (d, *J* = 12.2 Hz), 129.67, 127.93, 124.95, 122.42, 120.89, 120.70 (d, *J* = 9.9 Hz), 109.18 (d, *J* = 24.2 Hz), 102.94, 97.20 (d, *J* = 27.1 Hz), 52.63, 51.73, 31.77, 31.06 *Two peaks are missing due to overlap. Minor isomer* δ 169.38, 149.91, 139.80 (d, *J* = 4.2 Hz), 138.68, 137.44 (d, *J* = 12.2 Hz), 128.87, 126.67, 122.45, 120.90, 102.74, 52.69, 51.59, 26.79, 26.05. *Eight Peaks are missing due to C-F coupling and overlap*; **^{19}F NMR (282 MHz, CDCl$_3$)** *Major isomer:* δ -121.01 (td, *J* = 9.8, 5.6 Hz); *Minor isomer:* δ -121.02; **HRMS** m/z (ESI): calcd. for $C_{22}H_{21}N_2O_4FNa$ 419.1378, found 419.1375; **ATR-IR** ν (cm^{-1}) 3005, 2955, 1748, 1732, 1620, 1559, 1470, 1435, 1385, 1350, 1273, 1231, 1204, 1150, 1107, 1049, 1018, 980, 833, 810, 779, 748.

(*E*/*Z*)-dimethyl 2-(4-(5-bromo-1-(pyridin-2-yl)-1*H*-indol-2-yl)but-2-en-1-yl)malonate[8]

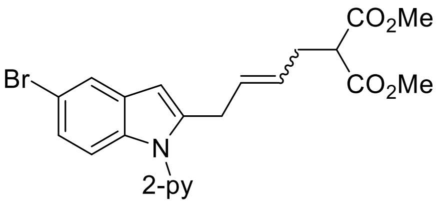

Following the general procedure, product **4c** was isolated in 63% yield. **^{1}H NMR (400 MHz, CDCl$_3$)** δ 8.69 – 8.59 (m, 1H), 7.95 – 7.85 (m, 1H), 7.67 (t, *J* = 1.2 Hz, 1H), 7.42 – 7.31 (m, 2H), 7.19 (d, *J* = 1.3 Hz, 2H), 6.36 (s, 0.26H), 6.34 (s, 0.69H), 5.68 – 5.55 (m, 1H), 5.46 – 5.27 (m, 1H), 3.70 (s, 4.07 H), 3.70 (s, 1.8 H), 3.62 (d, *J* = 7.3 Hz, 0.58H), 3.52 (d, *J* = 6.9 Hz, 1.50H), 3.39 – 3.30 (m, 1H), 2.61 (td, *J* = 7.6, 1.5 Hz, 0.59H), 2.54 (td, *J* = 7.4, 1.2 Hz, 1.48H); **^{13}C NMR (101 MHz, CDCl$_3$)** δ *Major isomer* 169.34, 150.98, 149.78, 140.73, 138.56, 136.09, 130.29, 129.41, 128.15, 124.66, 122.62, 122.52, 121.09, 113.92, 111.75, 102.45, 52.65, 51.70, 31.77, 31.02 *Two peaks are missing due to overlap. Minor isomer* δ 169.37, 149.92, 140.78, 138.66, 136.15, 130.31, 128.60, 126.88, 122.54,

121.10, 111.73, 102.27, 52.71, 51.55, 26.80, 26.01. *Six Peaks are missing due to overlap*. **HRMS** m/z (ESI): calcd. for $C_{22}H_{21}N_2O_4BrNa$ 479.0577, found 479.0576; **ATR-IR** ν (cm^{-1}) 3005, 2951, 1748, 1732, 1586, 1543, 1435, 1385, 1339, 1273, 1231, 1204, 1150, 1049, 1015, 972, 864, 783, 733.

(*E*/*Z*)-dimethyl 2-(4-(5-iodo-1-(pyridin-2-yl)-1*H*-indol-2-yl)but-2-en-1-yl)malonate[8]

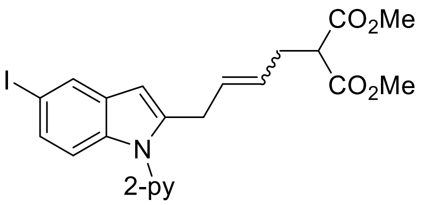

Following the general procedure, product **4d** was isolated in 52% yield. **^{1}H NMR (400 MHz, CDCl$_3$)** δ 8.62 – 8.54 (m, 1H), 7.88 – 7.77 (m, 2H), 7.34 – 7.25 (m, 3H), 7.02 (d, *J* = 8.6 Hz, 1H), 6.27 (s, 0.27H), 6.25 (s, 0.58H), 5.63 – 5.46 (m, 1H), 5.41 – 5.19 (m, 1H), 3.63 (s, 4.23H), 3.62 (s, 1.71H), 3.54 (d, *J* = 7.3 Hz, 0.63H), 3.45 (d, *J* = 6.4 Hz, 1.30H), 3.33 – 3.23 (m, 1H), 2.53 (t, *J* = 7.5 Hz, 0.59H), 2.47 (t, *J* = 7.2 Hz, 1.34H); **^{13}C NMR (101 MHz, CDCl$_3$)** *Major isomer* δ 169.33, 150.93, 149.78, 140.38, 138.55, 136.58, 131.08, 130.20, 129.41, 128.89, 128.14, 122.52, 121.09, 112.26, 102.16, 84.27, 52.65, 51.70, 31.77, 30.95. *Two peaks are missing due to overlap*. *Minor isomer* δ 169.36, 149.92, 140.44, 138.65, 136.64, 128.61, 126.88, 122.54, 112.24, 101.98, 52.71, 51.55, 26.79, 25.95. *Eight Peaks are missing due to overlap*; **HRMS** m/z (ESI): calcd. for $C_{22}H_{21}IN_2NaO_4$ 527,0444, found 527,0438;

(*E*/*Z*)-dimethyl 2-(4-(5-methoxy-1-(pyridin-2-yl)-1*H*-indol-2-yl)but-2-en-1-yl)malonate[8]

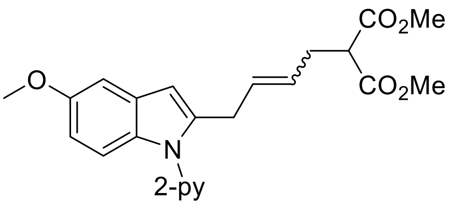

Following the general procedure, product **4e** was isolated in 90% yield. **^{1}H NMR (400 MHz, CDCl$_3$)** δ 8.61 – 8.48 (m, 1H), 7.86 – 7.70 (m, 1H), 7.34 (d, *J* = 8.0 Hz, 0.27H), 7.30 (d, *J* = 8.0 Hz, 0.77H), 7.23 – 7.13 (m, 2H), 6.95 (d, *J* = 2.4 Hz, 1H), 6.69 (dd, *J* = 8.9, 2.5 Hz, 1H), 6.28 (s, 0.25H), 6.25 (s, 0.70H), 5.60 – 5.46 (m, 1H), 5.36 – 5.20 (m, 1H), 3.76 (s, 3H), 3.62 (s, 6H), 3.56 (d, *J* = 7.3 Hz, 0.57H), 3.47 (d, *J* = 6.6 Hz, 1.46H), 3.22 – 3.21 (m, 1H), 2.55 (t, *J* = 7.2 Hz, 0.53H), 2.46 (t, *J* = 7.2 Hz, 1.43H); **^{13}C NMR (101 MHz, CDCl$_3$)** *Major isomer* δ 169.34, 154.86, 151.48, 149.54, 139.89, 138.31, 132.43, 129.88, 129.14, 127.67, 121.88, 120.75, 111.40, 111.01, 102.98, 102.30, 55.90, 52.58, 51.72, 31.76, 31.13. *Two peak is missing due to overlap*. *Minor isomer* δ 169.36, 149.68, 139.95, 138.41, 132.47, 129.10, 126.39, 121.90, 110.99, 102.80, 52.64, 51.59, 26.76, 26.13. *Nine*

peaks are missing due to overlap.

(*E*/*Z*)-dimethyl 2-(4-(3-methyl-1-(pyridin-2-yl)-1*H*-indol-2-yl)but-2-en-1-yl)malonate[8]

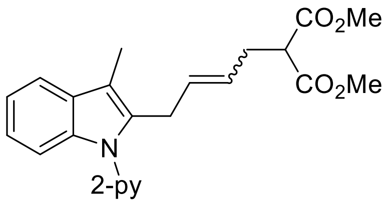

Following the general procedure, [$MnBr(CO)_5$] (10 mol%) and NaOAc (20 mol%) were used to replace $Mn_2(CO)_{10}$, product **4f** was isolated in 89% yield. **^{1}H NMR (400 MHz, $CDCl_3$)** δ 8.58 – 8.49 (m, 1H), 7.81 – 7.72 (m, 1H), 7.49 – 7.42 (m, 1H), 7.30 (t, *J* = 8.3 Hz, 1H), 7.28 – 7.15 (m, 2H), 7.10 – 7.01 (m, 2H), 5.46 – 5.35 (m, 0.64H), 5.33 – 5.22 (m, 0.37H), 5.14 – 4.98 (m, 1H), 3.66 – 3.60 (m, 2.91H), 3.56 (s, 3.83H), 3.50 (d, *J* = 5.9 Hz, 1.32H), 3.22 – 3.10 (m, 1H), 2.43 (t, *J* = 7.4 Hz, 0.72H), 2.35 (t, *J* = 7.3 Hz, 1.32H), 2.25 (s, 1.08H), 2.20 (s, 2.20H); **^{13}C NMR (101 MHz, $CDCl_3$)** *Major isomer* δ 169.30, 151.70, 149.51, 138.21, 136.54, 133.94, 130.27, 129.42, 126.46, 122.05, 121.77, 121.03, 120.28, 118.35, 110.69, 110.12, 52.50, 51.76, 31.71, 28.19, 8.77. *Two peak is missing due to overlap*. *Minor isomer* δ 169.36, 149.74, 138.32, 136.58, 134.76, 129.86, 129.47, 125.14, 122.06, 121.08, 120.32, 118.38, 110.37, 109.94, 52.62, 51.46, 26.74, 23.65, 8.87. *Four peaks are missing due to overlap*.

(*E*/*Z*)-diethyl 2-(4-(3-methyl-1-(pyridin-2-yl)-1*H*-indol-2-yl)but-2-en-1-yl)malonate

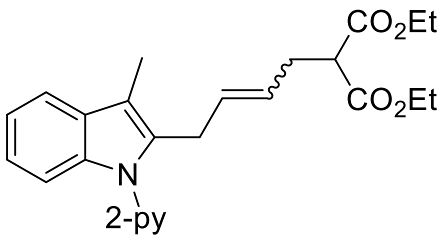

Following the general procedure, [$MnBr(CO)_5$] (10 mol%) and NaOAc (20 mol%) were used to replace $Mn_2(CO)_{10}$, product **4g** was isolated in 95% yield using diethyl 2-vinylcyclopropane-1,1-dicarboxylate (**2bb**) as the starting material. **^{1}H NMR (300 MHz, $CDCl_3$)** δ 8.58 – 8.50 (m, 1H), 7.81 – 7.71 (m, 1H), 7.51 – 7.42 (m, 1H), 7.36 – 7.14 (m, 3H), 7.09 – 7.00 (m, 2H), 5.48 – 5.34 (m, 0.7H), 5.32 – 5.20 (m, 0.3H), 5.17 – 4.97 (m, 1H), 4.18 – 3.93 (m, 4H), 3.63 (d, *J* = 6.7 Hz, 0.6H), 3.49 (d, *J* = 5.8 Hz, 1.3H), 3.19 – 3.06 (m, 1H), 2.43 (t, *J* = 7.4 Hz, 0.7H), 2.35 (t, *J* = 7.3 Hz, 1.4H), 2.25 (s, 1H), 2.20 (s, 2H), 1.17 (t, *J* = 7.1 Hz, 2H), 1.10 (t, *J* = 7.1 Hz, 4.2H); **^{13}C NMR (75 MHz, $CDCl_3$)** *Major isomer:* δ 168.93, 151.68, 149.48, 138.21, 136.51, 133.94, 130.05, 129.40, 126.65, 122.01, 121.76, 121.04, 120.25, 118.32, 110.64, 110.09, 61.38, 52.05, 31.63, 28.17, 14.09, 8.77, *Two peaks are missing due to overlap*; *Minor isomer:* δ 169.00, 149.71, 138.31, 136.55, 134.81, 129.63, 129.45, 125.34, 121.07, 120.29, 118.36, 110.33, 109.93, 61.50, 51.78, 26.66, 23.66, 14.17, 8.87; *Five peaks are missing due to overlap*; **HRMS**: m/z (ESI)

calcd for $C_{25}H_{28}N_2NaO_4$ $[M+Na]^+$ 443,1947, found 443,1941.

(*E*/*Z*)-dimethyl 2-(4-(2-(pyridin-2-yl)phenyl)but-2-en-1-yl)malonate[8]

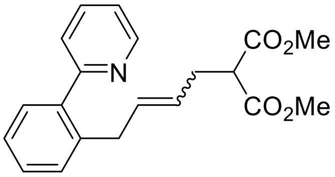

Following the general procedure, $[MnBr(CO)_5]$ (10 mol%) and NaOAc (20 mol%) were used to replace $Mn_2(CO)_{10}$, product **4h** was isolated in 57% yield. **^{1}H NMR (300 MHz, $CDCl_3$)** δ 8.67 – 8.55 (m, 1H), 7.73 – 7.62 (m, 1H), 7.33 – 7.14 (m, 6H), 5.58 – 5.41 (m, 1H), 5.29 – 5.10 (m, 1H), 3.64 (s, 1.68H), 3.61 (s, 4.27H), 3.45 (d, *J* = 7.3 Hz, 0.56H), 3.34 (d, *J* = 6.5 Hz, 1.48H), 3.31 – 3.22 (m, 1H), 2.56 – 2.41 (m, 2H); **^{13}C NMR (75 MHz, $CDCl_3$)** *Major isomer* δ 169.43, 160.01, 149.31, 140.41, 138.03, 136.27, 132.73, 129.95, 129.91, 128.51, 126.57, 126.37, 124.27, 121.84, 52.64, 51.67, 36.23, 31.90. *Two peaks are missing due to overlap*; *Minor isomer* δ 160.01, 149.31, 138.42, 136.41, 131.93, 129.97, 129.77, 128.65, 126.33, 125.10, 124.31, 52.64, 51.67, 31.09, 26.79. *Five Peaks are missing due to overlap*; **HRMS** m/z (ESI): calcd. for $C_{20}H_{21}NNaO_4$ 362,1368, found 362,1363.

(*E*/*Z*)-dimethyl 2-(4-(5-methoxy-2-(pyridin-2-yl)phenyl)but-2-en-1-yl)malonate[8]

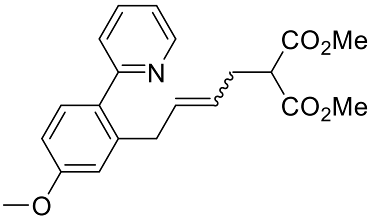

Following the general procedure, product **4i** was isolated in 50% yield. **^{1}H NMR (400 MHz, $CDCl_3$)** δ 8.66 – 8.51 (m, 1H), 7.70 – 7.57 (m, 1H), 7.32 – 7.21 (m, 2H), 7.14 (ddd, *J* = 7.5, 4.9, 1.1 Hz, 1H), 6.82 – 6.64 (m, 2H), 5.60 – 5.47 (m, 1H), 5.31 – 5.14 (m, 1H), 3.77 (s, 3H), 3.64 (s, 1.07H), 3.61 (s, 4.92H), 3.46 (d, *J* = 7.9 Hz, 0.34H), 3.35 (d, *J* = 6.6 Hz, 1.62H), 3.32 – 3.23 (m, 1H), 2.57 – 2.40 (m, 2H); **^{13}C NMR (101 MHz, $CDCl_3$)** *Major isomer* δ 169.43, 159.74, 159.67, 149.19, 139.69, 136.20, 133.20, 132.58, 131.25, 126.78, 124.28, 121.47, 115.47, 111.63, 55.40, 52.56, 51.89, 36.44, 31.90. *Two peaks are missing due to overlap. Minor isomer* δ ^{13}C NMR (75 MHz, $CDCl_3$) δ 159.83, 149.25, 140.06, 136.34, 131.81, 131.31, 125.31, 124.32, 115.32, 111.58, 52.63, 51.67, 31.30, 26.81. *Seven peaks are missing due to overlap*; **HRMS** m/z (ESI): calcd. for $C_{21}H_{24}NO_5$ 370,1654, found 370,1649.

(*E*/*Z*)-Dimethyl-2-(4-(4-(pyridin-2-yl)thiophen-3-yl)but-2-en-1-yl)malonate

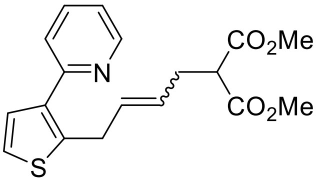

Following the general procedure, product **4j** was isolated in 53% yield. **^{1}H NMR (400 MHz, $CDCl_3$)** δ 8.62 – 8.53 (m, 1H), 7.68 – 7.59 (m, 1H), 7.40 – 7.32 (m, 1H), 7.24 – 7.20 (m, 1H), 7.14 – 7.05

(m, 2H), 5.76 – 5.61 (m, 1H), 5.51 – 5.28 (m, 1H), 3.87 (dd, *J* = 7.3, 1.6 Hz, 0.50H), 3.74 (dd, *J* = 6.6, 1.4 Hz, 1.49H), 3.66 (s, 1.43H), 3.64 (s, 4.56H), 3.40 – 3.31 (t, *J* = 7.6 Hz, 1H), 2.68 (td, *J* = 7.6, 1.6 Hz, 0.50H), 2.55 (m, 1.50H); **^{13}C NMR (101 MHz, $CDCl_3$)** *Major isomer* δ = 169.41, 155.14, 149.54, 141.68, 137.25, 136.44, 131.91, 128.84, 127.56, 122.84, 122.72, 121.45, 52.65, 51.81, 32.21 31.81 *Two peaks are missing due to overlap, Minor isomer* δ = 169.44, 155.23, 141.98, 137.06, 136.50, 131.14, 128.82, 126.12, 122.57, 121.44, 52.70, 51.69, 27.17, 26.90 *Four peaks are missing due to overlap*; **HRMS** m/z (ESI): calcd. for $C_{18}H_{19}NO_4SNa$ 368.0927, found 368.0929; **ATR-IR** ν (cm^{-1}) 3005, 2951, 1748, 1732, 1586, 1566, 1532, 1474, 1435, 1339, 1269, 1235, 1200, 1150, 1022, 972, 856, 791, 745, 718, 702.

2.3) Procedure and analytical data of compounds 5.

General procedure: In a 10 mL dry Schlenk tube with a stirring bar, *N*-2-pyridylindole (**1**) (0.20 mmol), [$MnBr(CO)_5$] (10 mol%, 5.5 mg) and NaOAc (20 mol%, 3.3 mg) were added under air. Then the reaction vessel was evacuated and filled with argon for three times; Afterwards, diazabicycle (**2ca**) (0.30 mmol) and Et_2O (1 mL, anhydrous, no solvent was added under neat condition) were added under an argon atmosphere. The tube was sealed and the mixture was stirred at 90 °C for the determined time. Then, the volatiles were removed and the analytically pure product was obtained by flash chromatography (silica; gradient of pentane/EtOAc).

diethyl 1-(2-(1-(pyridin-2-yl)-1*H*-indol-2-yl)cyclopent-3-en-1-yl)hydrazine-1,2-dicarboxylate

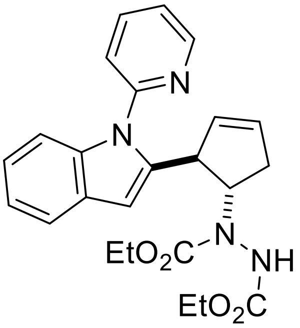

Following the general procedure, product **5a** was isolated in 83% yield. **^{1}H NMR (400 MHz, CD_3OD)** δ 8.61 (d, *J* = 3.9 Hz, 1H), 8.02 (td, *J* = 7.8, 1.9 Hz, 1H), 7.56 (d, *J* = 7.9 Hz, 1H), 7.54 – 7.49 (m, 1H), 7.45 (dd, *J* = 7.5, 5.0 Hz, 1H), 7.20 – 7.13 (m, 1H), 7.11 – 7.03 (m, 2H), 6.51 (s, 1H), 5.80 – 5.68 (m, 1H), 5.59 (s, 1H), 5.12 (s, 1H), 4.45 – 4.27 (m, 1H), 4.21 – 3.86 (m, 4H), 2.66 (dd, *J* = 17.2, 8.4 Hz, 1H), 2.57 – 2.36 (m, 1H), 1.39 – 0.96 (m, 6H); **^{13}C NMR (101 MHz, CD_3OD)** δ 157.53, 155.93, 150.98, 149.13, 141.82, 139.23, 137.91, 130.94, 129.06, 128.39, 122.82, 122.57, 121.67, 120.37, 119.81, 109.58, 101.28, 64.89, 62.11, 61.39, 46.10, 34.60, 13.49, 13.38; **HRMS**: m/z (ESI) calcd for $C_{24}H_{26}N_4NaO_4$ $[M+Na]^+$ 457,1852, found 457,1846. **ATR-IR** ν (cm^{-1}) 3279, 2982, 2931, 1748, 1701, 1589, 1469, 1454, 1435, 1142, 1230, 1211, 1126, 1057, 953, 926, 787, 741, 702. 3005.

di-tert-butyl 1-(-2-(1-(pyridin-2-yl)-1*H*-indol-2-yl)cyclopent-3-en-1-yl)hydrazine-1,2-dicarboxylate

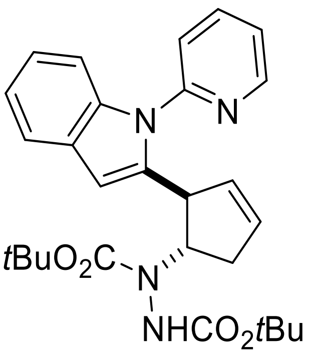

Following the general procedure, product **5b** was isolated in 70% yield using di-tert-butyl 2,3-diazabicyclo[2.2.1]hept-5-ene-2,3-dicarboxylate (**2cb**) as the starting material. **^{1}H NMR (600 MHz, CD_3OD)** δ 8.62 (d, *J* = 5.0 Hz, 1H), 8.01 (s, 1H), 7.65 – 7.40 (m, 3H), 7.21 – 7.12 (m, 1H), 7.12 – 6.98 (m, 2H), 6.54 (s, 1H), 5.69 (s, 1H), 5.52 (s, 1H), 5.15 (s, 1H), 4.29 (s, 1H), 2.67 – 2.45 (m, 2H), 1.65 – 1.05 (m, 18H); **^{13}C NMR (126 MHz, CD_3OD)** δ 157.79, 156.45, 152.42, 150.57, 150.46, 140.60, 139.32, 132.49, 130.21, 129.84, 124.23, 123.96, 122.95, 121.72, 121.11, 110.94, 102.65, 82.15, 81.63, 64.05, 47.25, 35.76, 28.61, 28.50; **HRMS**: m/z (ESI) calcd for $C_{28}H_{34}N_4NaO_4$ $[M+Na]^+$ 513.2478, found 513.2472.

diethyl 1-(-2-(5-methoxy-1-(pyridin-2-yl)-1*H*-indol-2-yl)cyclopent-3-en-1-yl)hydrazine-1,2-dicarboxylate

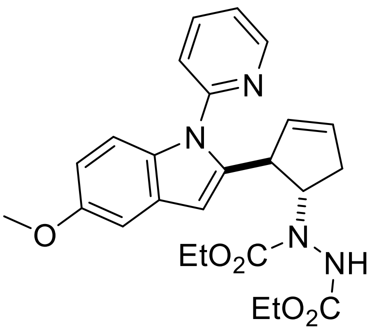

Following the general procedure, product **5c** was isolated in 94% yield. **^{1}H NMR (400 MHz, CD_3OD)** δ 8.59 (d, *J* = 3.8 Hz, 1H), 8.09 – 7.92 (m, 1H), 7.52 (s, d, *J* = 7.9 Hz, 1H), 7.43 (dd, *J* = 7.1, 5.2 Hz, 1H), 7.09 (d, *J* = 8.9 Hz, 1H), 7.04 (d, *J* = 2.3 Hz, 1H), 6.73 (dd, *J* = 8.9, 2.4 Hz, 1H), 6.44 (s, 1H), 5.77 – 5.68 (m, 1H), 5.59 (s, 1H), 5.10 (s, 1H), 4.45 – 4.27 (m, 1H), 4.16 – 3.94 (m, 4H), 3.79 (s, 3H), 2.74 – 2.57 (m, 1H), 2.55 – 2.43 (m, 1H), 1.26 – 1.05 (m, 6H); **^{13}C NMR (101 MHz, CD_3OD)** δ 158.80, 157.30, 156.29, 152.47, 150.41, 143.81, 140.56, 134.35, 132.36, 130.37, 130.12, 123.96, 123.60, 112.64, 111.72, 103.33, 102.67, 66.21, 63.47, 62.76, 56.13, 47.52, 36.03, 14.85, 14.74; **HRMS**: m/z (ESI) calcd for $C_{25}H_{28}N_4NaO_5$ $[M+Na]^+$ 487,1957, found 487,1952.

diethyl 1-(-2-(6-fluoro-1-(pyridin-2-yl)-1*H*-indol-2-yl)cyclopent-3-en-1-yl)hydrazine-1,2-dicarboxylate

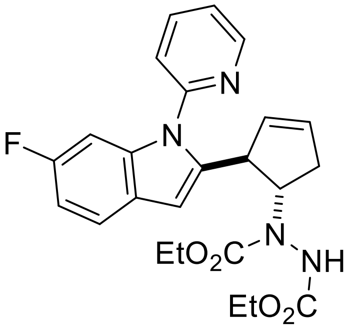

Following the general procedure, product **5d** was isolated in 81% yield. **^{1}H NMR (400 MHz, CD_3OD)** δ 8.63 (d, *J* = 3.7 Hz,

1H), 8.05 (t, *J* = 7.6 Hz, 1H), 7.56 (d, *J* = 7.9 Hz, 1H), 7.52 – 7.42 (m, 2H), 6.94 – 6.82 (m, 2H), 6.50 (s, 1H), 5.72 (dd, *J* = 5.7, 2.2 Hz, 1H), 5.58 (s, 1H), 5.10 (s, 1H), 4.32 (s, 1H), 4.18 – 3.95 (m, 4H), 2.78 – 2.57 (m, 1H), 2.56 – 2.38 (m, 1H), 1.30 – 1.12 (m, 6H); **^{13}C NMR (101 MHz, CD_3OD)** δ 161.21 (d, J = 237.5 Hz), 158.87, 157.28, 152.01, 150.66, 143.96, 140.77, 139.31, 139.19, 132.36, 130.57, 126.22, 124.47, 123.75, 122.03 (d, *J* = 9.7 Hz), 109.89(d, *J* = 24.3 Hz), 102.59, 97.63 (d, *J* = 27.4 Hz), 65.25, 63.53, 62.76, 47.58, 35.96, 14.84, 14.74; **^{19}F NMR (**564 **MHz, $CDCl_3$)** δ -122.62. **HRMS**: m/z (ESI) calcd for $C_{24}H_{25}FN_4NaO_4$ $[M+Na]^+$ 475,1758, found 475,1752.

diethyl 1-(-2-(3-methyl-1-(pyridin-2-yl)-1*H*-indol-2-yl)cyclopent-3-en-1-yl)hydrazine-1,2-dicarboxylate

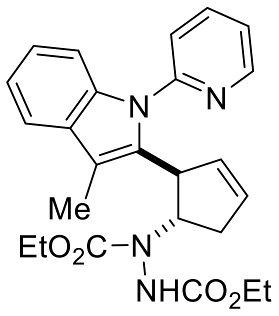

Following the general procedure, $[MnBr(CO)_5]$ (20 mol%) and NaOAc (40 mol%) were used under neat condition at 100 °C for 37 h, product **5e** was isolated in 94% yield. **^{1}H NMR (400 MHz, CD_3OD)** δ 8.58 (s, 1H), 7.97 (td, *J* = 7.8, 1.8 Hz, 1H), 7.54 – 7.48 (m, 1H), 7.48 – 7.38 (m, 2H), 7.13 – 7.00 (m, 3H), 5.71 – 5.51 (m, 2H), 5.33 (br, 1H), 4.34 (s, 1H), 4.20 – 3.88 (m, 4H), 2.71 – 2.47 (m, 2H), 2.34 (s, 3H), 1.30 – 0.88 (m, 6H); **^{13}C NMR (126 MHz, CD_3OD)** δ 158.64, 157.60, 153.05, 150.26, 140.20, 138.63, 137.56, 132.40, 131.06, 129.81, 124.52, 124.04, 123.09, 121.18, 118.97, 111.68, 110.74, 67.27, 63.37, 62.82, 47.18, 36.30, 14.71, 14.46, 9.03; **HRMS**: m/z (ESI) calcd for $C_{25}H_{28}N_4NaO_4$ $[M+Na]^+$ 471,2008, found 471,2003.

diethyl 1-(2-(2-(pyridin-2-yl)phenyl)cyclopent-3-en-1-yl)hydrazine-1,2-dicarboxylate[9]

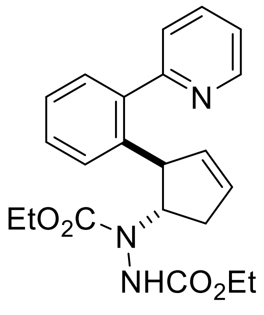

Following the general procedure, $[MnBr(CO)_5]$ (20 mol%) and NaOAc (40 mol%) were used under neat condition at 100 °C for 37 h. product **5f** was isolated in 78% yield. The structure was determined by comparison with the reported literature.[9] **^{1}H NMR (300 MHz, CD_3OD)** δ 8.78 – 8.49 (m, 1H), 7.90 (d, *J* = 1.8 Hz, 1H), 7.49 (dt, *J* = 7.8, 1.1 Hz, 1H), 7.46 – 7.16 (m, 5H), 5.78 (dd, *J* = 6.0, 2.5 Hz, 1H), 5.69 – 5.50 (m, 1H), 4.95 (s, 1H), 4.30 (s, 1H), 4.23 – 3.88 (m, 4H), 2.59 (m, 2H), 1.42 – 0.81 (m, 6H); **^{13}C NMR (75 MHz, CD_3OD)** δ 160.99, 158.58, 157.45, 149.36, 141.49, 138.69, 134.62, 131.04, 130.12, 128.95, 127.56, 126.69, 123.56,

69.99, 63.37, 62.78, 50.87, 36.14, 14.85, 14.66; **HRMS**: m/z (ESI) calcd for $C_{22}H_{25}N_3NaO_4$ $[M+Na]^+$ 418,1743, found 418,1737.

Diethyl 1-(2-(2-(5-methoxypyridin-2-yl)phenyl)cyclopent-3-en-1-yl)hydrazine-1,2-dicarboxylate[9]

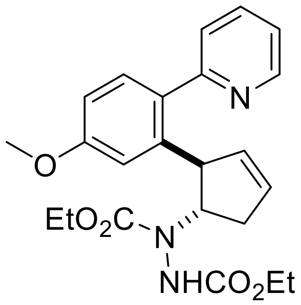

Following the general procedure, $[MnBr(CO)_5]$ (20 mol%) and NaOAc (40 mol%) were used under neat condition at 100 °C for 37 h. product **5g** was isolated in 72% yield. The structure was determined by comparison with the reported literature.[9] **^{1}H NMR (400 MHz, CD_3OD)** 8.78 – 8.44 (m, 1H), 7.86 (t, *J* = 7.3 Hz, 1H), 7.46 (d, *J* = 7.8 Hz, 1H), 7.41 – 7.31 (m, 1H), 7.25 (d, *J* = 9.0 Hz, 1H), 6.96 – 6.79 (m, 2H), 5.86 – 5.73 (m, 1H), 5.56 (s, 1H), 4.97 (s, 1H), 4.42 – 4.29 (m, 1H), 4.19 – 3.90 (m, 4H), 3.82 (s, 3H), 2.78 – 2.38 (m, 2H), 1.34 – 0.96 (m, 6H); **^{13}C NMR (101 MHz, CD_3OD)** δ 161.68, 160.84, 158.51, 157.53, 149.25, 138.66, 134.56, 134.13, 132.56, 130.29, 126.77, 123.19, 114.19, 113.11, 69.91, 63.40, 62.88, 55.73, 50.73, 36.01, 14.86, 14.64; **HRMS**: m/z (ESI) calcd for $C_{23}H_{27}N_3NaO_5$ $[M+Na]^+$ 448,1848, found 448,1843.

diethyl 1-(-2-((*Z*)-2-(pyridin-2-yl)prop-1-en-1-yl)cyclopent-3-en-1-yl)hydrazine-1,2-dicarboxylate

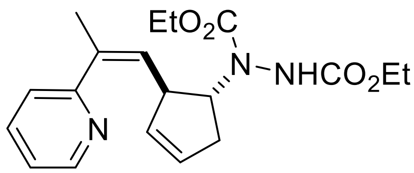

Following the general procedure, $[MnBr(CO)_5]$ (20 mol%) and NaOAc (40 mol%) were used at 100 °C for 24 h, product **5h** was isolated in 62% yield. **^{1}H NMR (400 MHz, CD_3OD)** δ 8.53 (s, 1H), 7.80 (t, *J* = 7.2 Hz, 1H), 7.37 (d, *J* = 7.9 Hz, 1H), 7.33 – 7.24 (m, 1H), 5.74 – 5.59 (m, 1H), 5.50 – 5.46 (m, 1H), 5.44 – 5.38 (m, 1H), 4.63 (br, 1H), 4.24 – 4.00 (m, 4H), 3.72 – 3.60 (m, 1H), 2.66 – 2.28 (m, 4H), 2.08 (d, *J* = 1.1 Hz, 3H), 1.28 – 1.15 (m, 6H); **^{13}C NMR (101 MHz, CD_3OD)** δ 160.65, 158.60, 157.80, 149.52, 138.52, 137.90, 133.62, 132.78, 129.93, 125.01, 123.47, 66.91, 63.48, 62.70, 36.50, 35.75, 24.45, 14.86, 14.83; **HRMS**: m/z (ESI) calcd for $C_{19}H_{25}N_3NaO_4$ $[M+Na]^+$ 382,1743, found 382,1748.

Determination of structure was achieved with TOCSY and NOE-Experiments

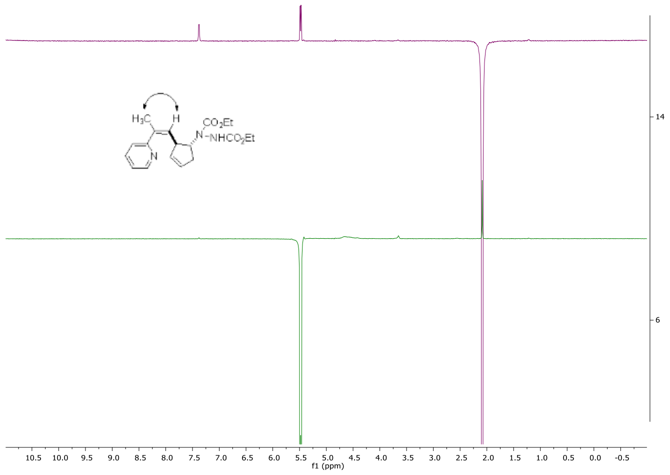

3) Gram-scale synthesis of 1a.

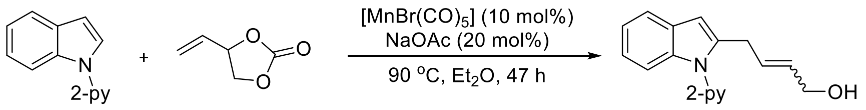

1a 6.0 mmol **2aa** 9.0 mmol **3a** 1.44g 91% yield, *E*/*Z* = 6.7

General procedure: In a 100 mL dry Schlenk tube with a stirring bar, *N*-2-pyridylindole (**1a**) (6.0 mmol), [MnBr(CO)$_5$] (10 mol%, 165 mg) and NaOAc (20 mol%, 98.4 mg) were added under air. Then the reaction vessel was evacuated and filled with argon for three times; Afterwards, vinyl-1,3-dioxolan-2-one (**2aa**) (9.0 mmol) and Et$_2$O (20 mL, anhydrous) were added under an argon atmosphere. The tube was sealed and the mixture was stirred at 90 °C for 47 h. Then, the volatiles were removed and the analytically pure product was obtained in 91% yield (1.44 g, *E*/*Z* = 6.7) by flash chromatography (silica; gradient of pentane/EtOAc).

4) Radical trapping experiments.

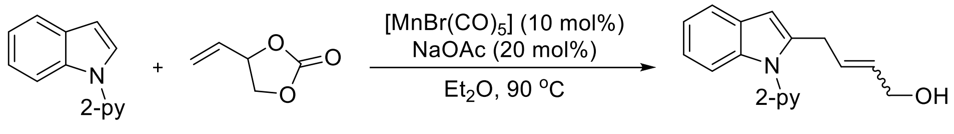

1a **2aa** **3a**

optimized condition	90%
with BHT (1.0 eq.)	91%
with 1,1-diphenylethylene (1.0 eq.)	90%

Scheme S1. Radical trapping experiments, isolated yield.

General procedure: In a 10 mL dry Schlenk tube with a stirring bar, *N*-2-pyridylindole (**1a**) (0.2

mmol), radical inhibitor (0.2 mmol), [MnBr(CO)$_5$] (10 mol%, 5.5 mg) and NaOAc (20 mol%, 3.2 mg) were added under air. Then the reaction vessel was evacuated and filled with argon for three times; Afterwards, vinyl-1,3-dioxolan-2-one (**2aa**) (0.3 mmol) and Et_2O (1 mL, anhydrous) were added under an argon atmosphere. The tube was sealed and the mixture was stirred at 90 °C for 5 h. Then, the volatiles were removed and the analytically pure product was obtained by flash chromatography (silica; gradient of pentane/EtOAc).

5) H/D scrambling experiments.

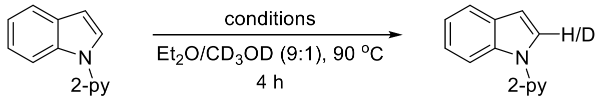

1a **1a'**

Conditions	Deuterated ratio in 2-position
[MnBr(CO)$_5$] (10 mol%) NaOAc (20 mol%)	85%
[MnBr(CO)$_5$] (10 mol%)	71%
NaOAc (20 mol%)	0%

Scheme S2. H/D scrambling experiments.

General procedure: In a 10 mL dry Schlenk tube with a stirring bar, *N*-2-pyridylindole (**1a**) (0.2 mmol), [MnBr(CO)$_5$] (10 mol%, 5.5 mg) and/or NaOAc (20 mol%, 3.2 mg) were added under air. Then the reaction vessel was evacuated and filled with argon for three times; Afterwards, Et_2O (0.9 mL, anhydrous) and CD_3OD (0.1 mL) were added under an argon atmosphere. The tube was sealed and the mixture was stirred at 90 °C for 4 h. Then, the volatiles were removed and the analytically pure product was obtained by flash chromatography (silica; gradient of pentane/EtOAc). The H/D-ratio was determined by ^{1}H NMR.

Condition: NaOAc and $MnBr(CO)_5$

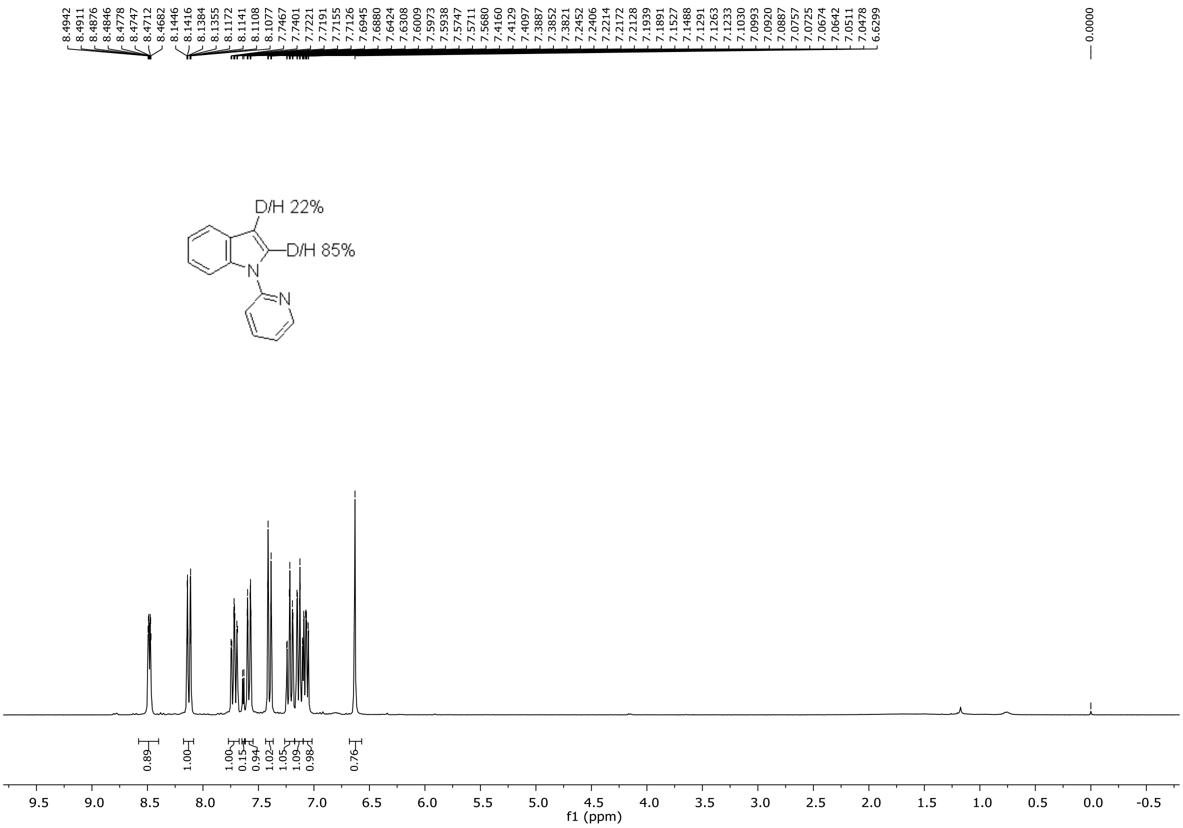

Condition: $MnBr(CO)_5$

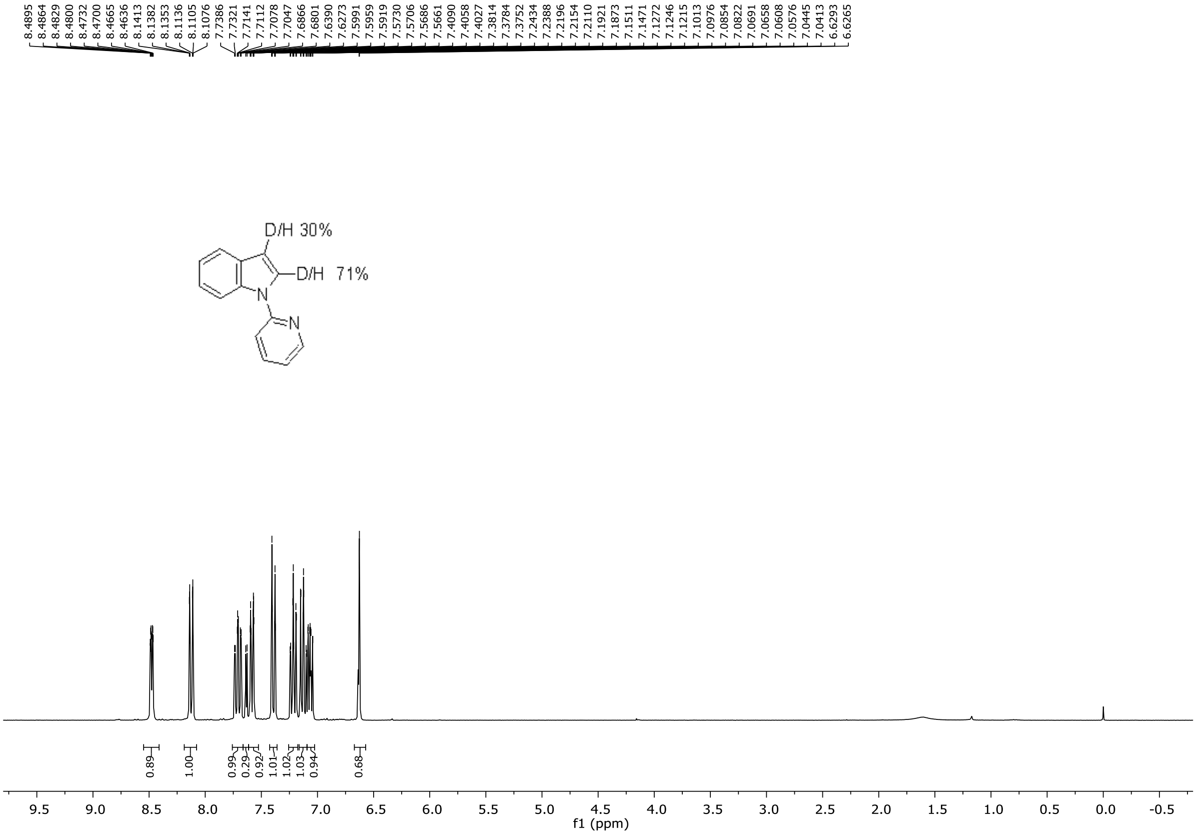

Condition: NaOAc

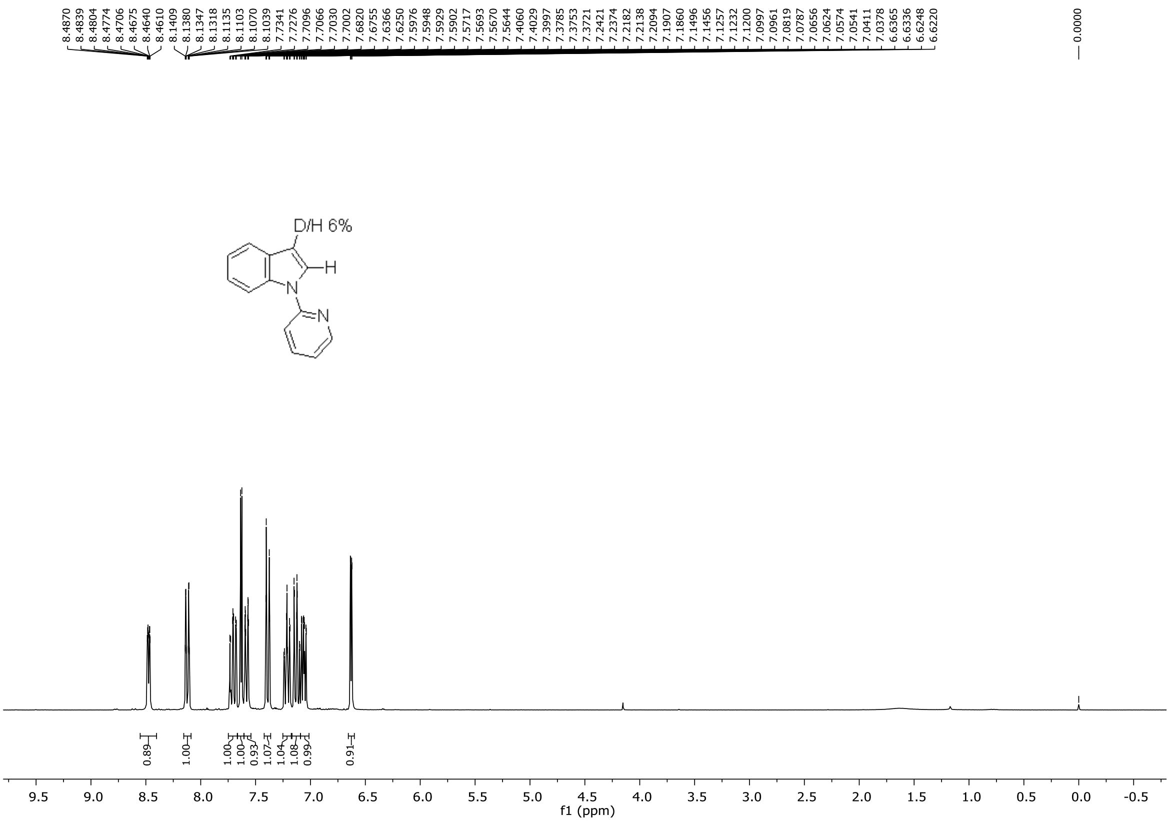

1-(pyridin-2-yl)-1*H*-indole

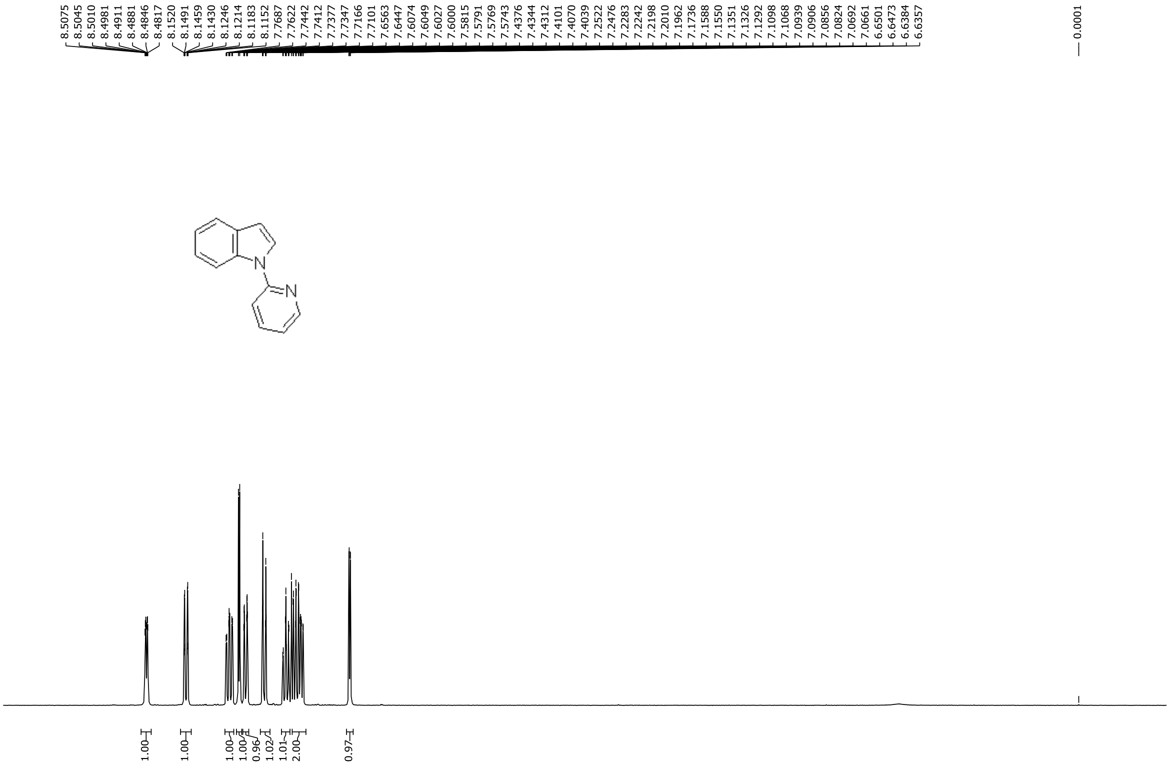

6) KIE experiments.

Parallel experiments for the synthesis of allylic alcohol.

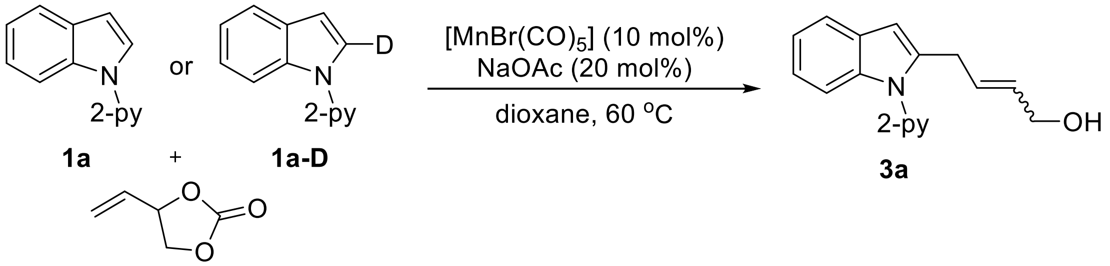

Following the general procedure 2.1, *N*-2-pyridylindole (**1a**) (0.20 mmol) or 2-deuterated *N*-2-pyridylindole (**1a-D**) (0.20 mmol), [MnBr(CO)$_5$] (10 mol%, 5.5 mg) NaOAc (20 mol%, 3.3 mg), 1,3,5-trimethoxybenzene (0.068 mmol, internal standard), vinyl-1,3-dioxolan-2-one (**2aa**) (0.30 mmol) and dioxane (2 mL, anhydrous) were added under an argon atmosphere in two different reaction tubes. The reactions were stirred at 60 °C and samples of 100 μL were taken at intervals to analyze by ^{1}H NMR. KIE value was measured averaging for each run. Note: The yields of **3a** are 55% and 54% respectively when **1a** or **1a-D** was submitted under the optimized condition for 5 minutes, therefore, 60 ^{o}C was used.

Reaction Time	Yield of **3a** (**1a** was used) %	Yield of **3a** (**1a-D** was used) %
5 min	11	10
10 min	32	30
30 min	49	48
k_H/k_D (on average)	1.0	

7) Proposed mechanism.

The proposed reaction pathway is depicted in Scheme S3. The reaction starts with the base-assisted cyclomanganation of *N*-(2-pyridyl)-indole **1a**, forming a five-membered manganacycle **I**. Afterwards, coordination of vinyl-1,3-dioxolan-2-one (**2aa**) to manganacycle **I** is followed by a migratory insertion to furnish the seven-membered manganacycle **II**, which undergoes β-oxygen elimination and subsequent protonolysis to deliver the desired product **3a** and regenerate the active manganese-(I) complex. Here the *anti*-β-oxygen elimination of the manganacycle **II** to afford the *trans*-adduct as a major product might be a favorable pathway.

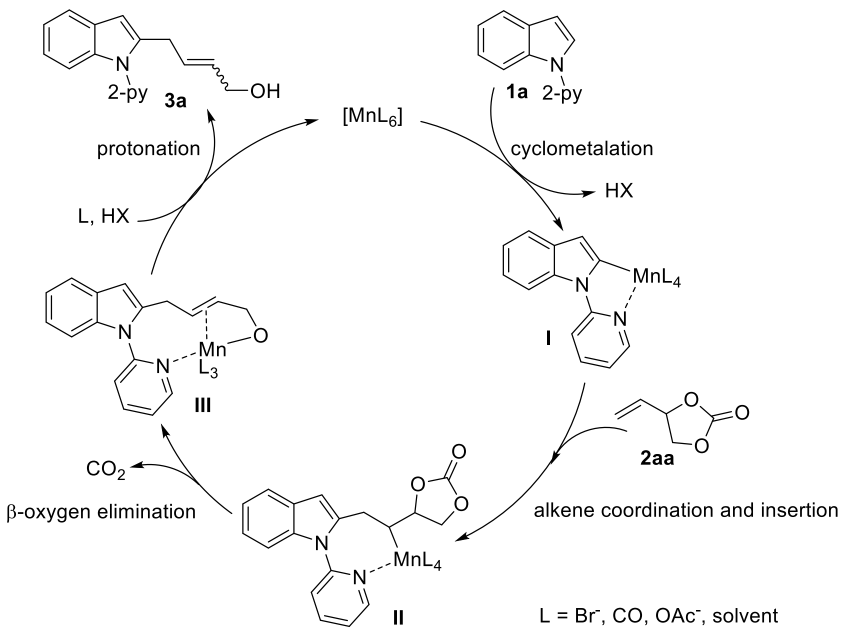

Scheme S3. Proposed mechanism.

References

[1] a) K. Seth, M. Nautiyal, P. Purohit, N. Parikh, A. K. Chakraborti, *Chem. Commun.* **2015**, *51*, 191-194; b) C. Sagnes, G. Fournet, B. Joseph, *Synlett* **2009**, *2009*, 433-436; c) V. K. Tiwari, N. Kamal, M. Kapur, *Org. Lett.* **2015**, *17*, 1766-1769.

[2] S. Xu, X. Huang, X. Hong, B. Xu, *Org. Lett.* **2012**, *14*, 4614-4617.

[3] a) S. Kathiravan, I. A. Nicholls, *Chem. Commun.* **2014**, *50*, 14964-14967; b) W. Rauf, A. L. Thompson, J. M. Brown, *Chem. Commun.* **2009**, 3874-3876; c) Y. Zou, G. Yue, J. Xu, J. Zhou, *Eur. J. Org. Chem.* **2014**, *2014*, 5901-5905.

[4] C. Chatalova-Sazepin, Q. Wang, G. M. Sammis, J. Zhu, *Angew. Chem. Int. Ed.* **2015**, *54*, 5443-5446.

[5] A. T. Parsons, M. J. Campbell, J. S. Johnson, *Org. Lett.* **2008**, *10*, 2541-2544; b) K. Miura, N. Fujisawa and A. Hosomi, *J. Org. Chem.* 2004, **69**, 2427.

[6] F. Menard, C. F. Weise, M. Lautens, *Org. Lett.* **2007**, *9*, 5365-5367.

[7] S. Yu, X. Li, *Org. Lett.* **2014**, *16*, 1200-1203.

[8] D. Zell, Q. Bu, M. Feldt, L. Ackermann, *Angew. Chem. Int. Ed.* **2016**, *55*, 7408-7412.

[9] Y. Zhang, Q. Wu, S. Cui, *Chem. Sci.* **2014**, *5*, 297-302.

NMR Spectra of Products

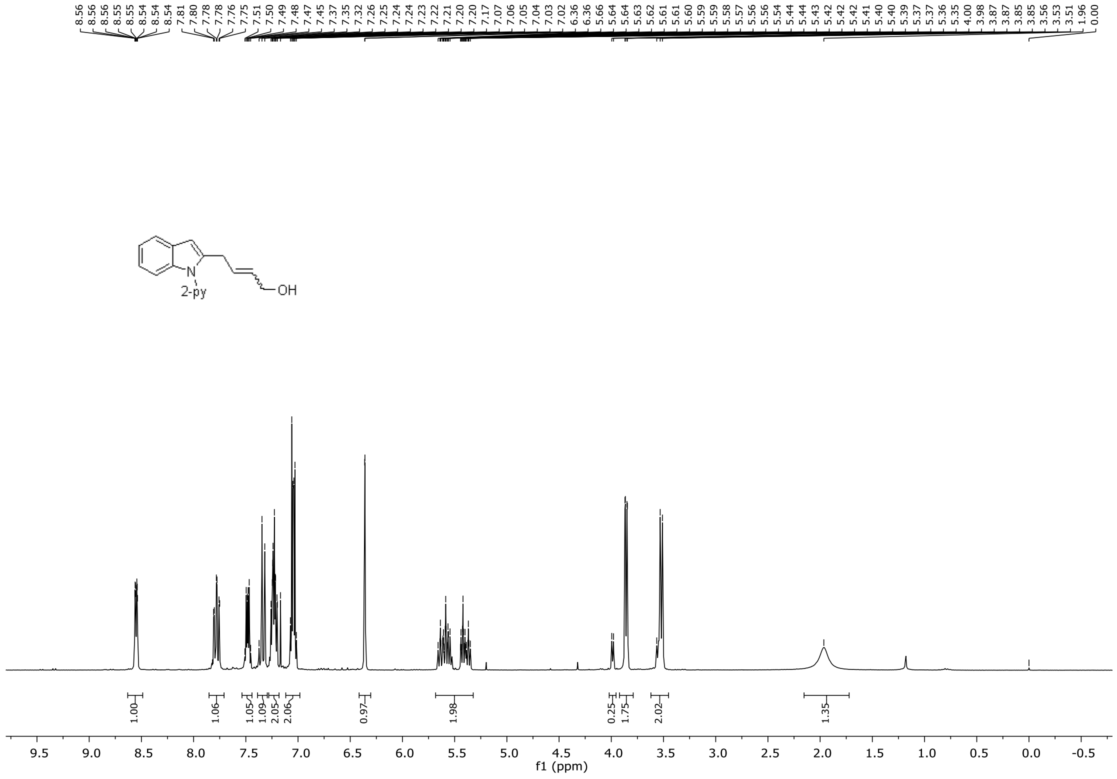

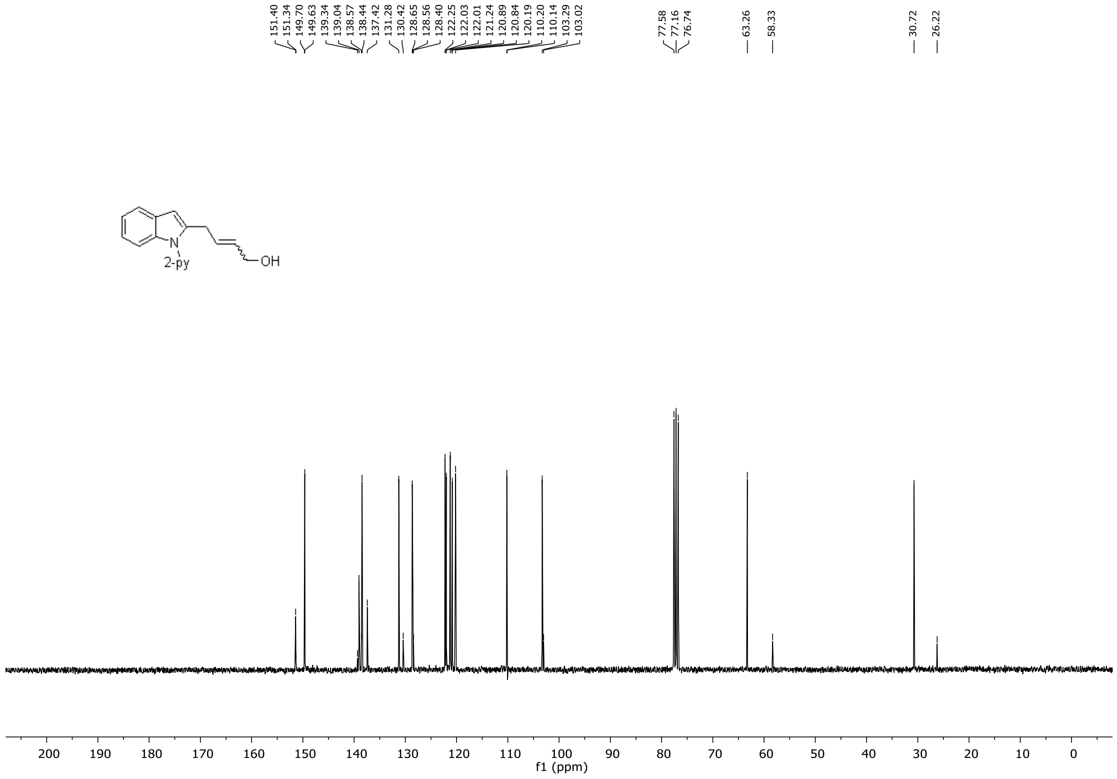

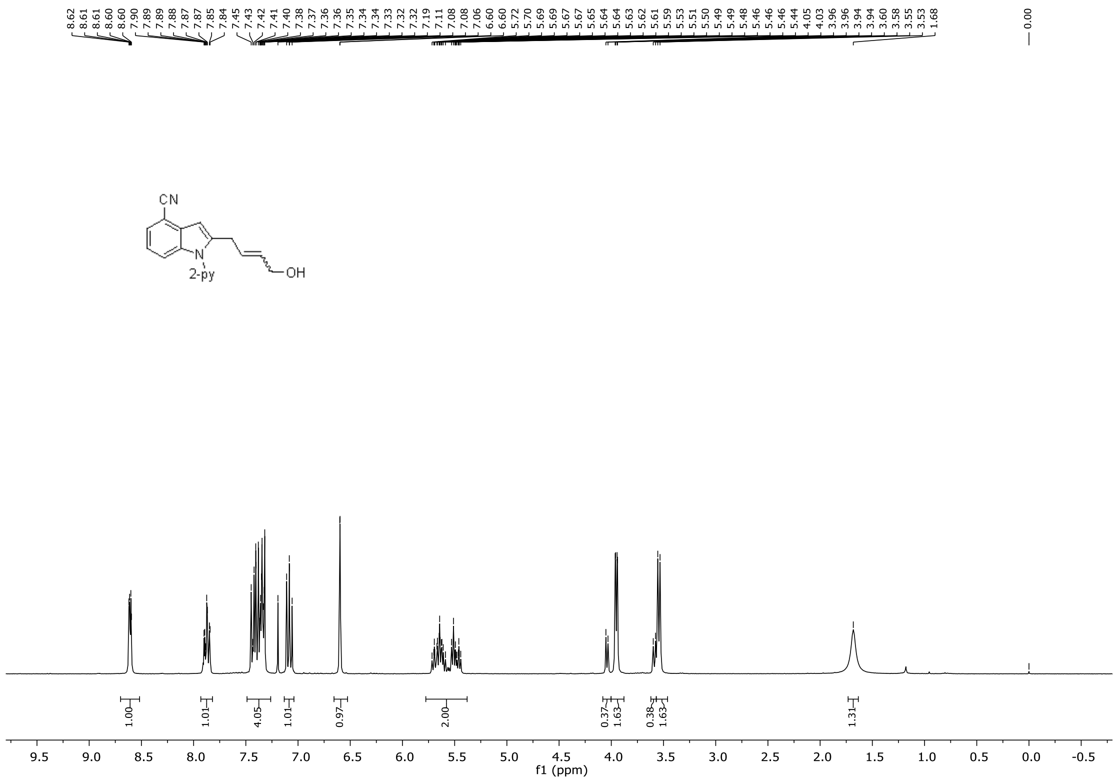
8.62 8.61 8.61 8.60 7.90 7.89 7.89 7.88 7.87 7.87 7.85 7.84 7.45 7.43 7.42 7.41 7.40 7.38 7.37 7.36 7.35 7.34 7.34 7.33 7.32 7.32 7.19 7.11 7.08 7.08 7.06 6.60 6.60 5.72 5.70 5.69 5.69 5.67 5.65 5.64 5.64 5.63 5.62 5.61 5.59 5.53 5.51 5.50 5.49 5.49 5.48 5.46 5.46 5.44 4.05 4.03 3.96 3.96 3.94 3.94 3.60 3.58 3.55 3.53 1.68
0.00
CN
N
2-py
OH
1.00
1.01
4.05
1.01
0.97
2.00
0.37
1.63
0.38
1.63
1.31
9.5 9.0 8.5 8.0 7.5 7.0 6.5 6.0 5.5 5.0 4.5 4.0 3.5 3.0 2.5 2.0 1.5 1.0 0.5 0.0 -0.5
f1 (ppm)

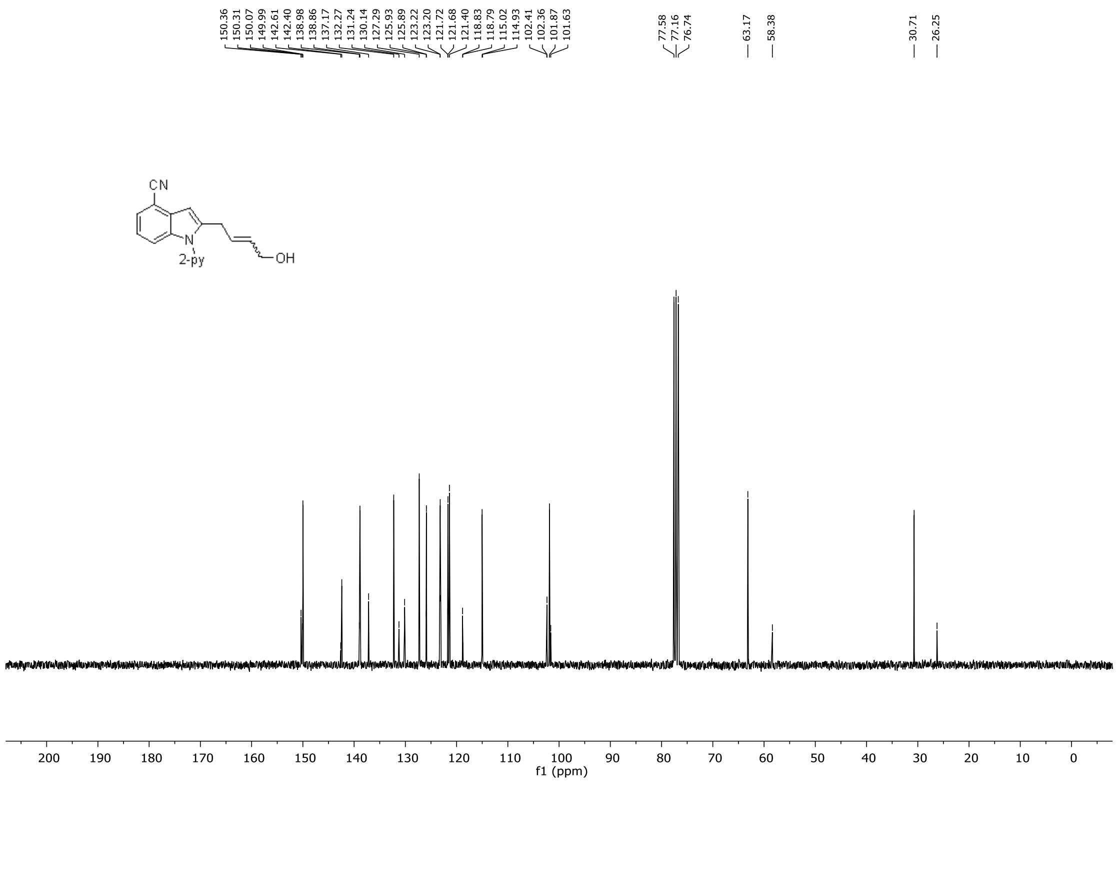
150.36 150.31 150.07 149.99 142.61 142.40 138.98 138.86 137.17 132.27 131.24 130.14 127.29 125.93 125.89 123.22 123.20 121.72 121.68 121.40 118.83 118.79 115.02 114.93 102.41 102.36 101.87 101.63
77.58 77.16 76.74
63.17
58.38
30.71
26.25
CN
N
2-py
OH
200 190 180 170 160 150 140 130 120 110 100 90 80 70 60 50 40 30 20 10 0
f1 (ppm)

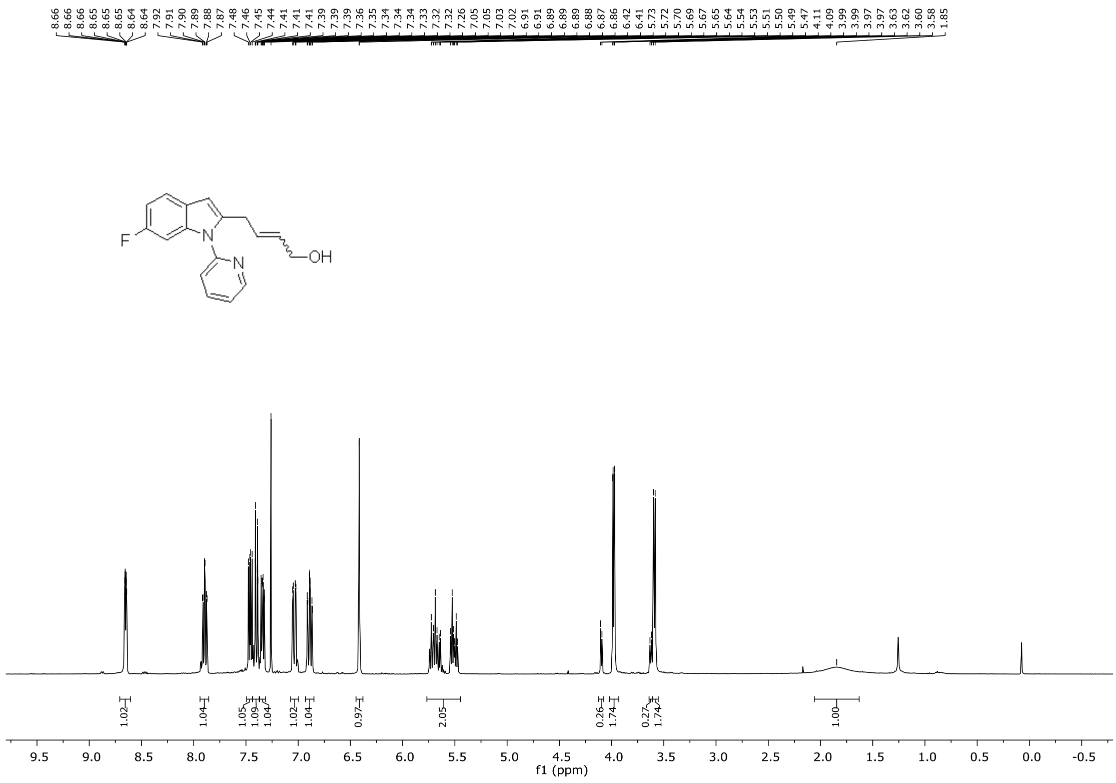

8.66 8.66 8.66 8.65 8.65 8.65 8.64 8.64 7.92 7.91 7.90 7.89 7.88 7.87 7.48 7.46 7.45 7.44 7.41 7.41 7.41 7.39 7.39 7.39 7.39 7.36 7.35 7.34 7.34 7.34 7.33 7.32 7.32 7.26 7.05 7.05 7.03 7.02 6.91 6.91 6.89 6.89 6.89 6.88 6.87 6.86 6.42 6.41 5.73 5.72 5.70 5.69 5.67 5.65 5.64 5.54 5.53 5.51 5.50 5.49 5.47 4.11 4.09 3.99 3.99 3.97 3.97 3.63 3.62 3.60 3.58 1.85
F
N
N
OH
1.02 1.04 1.05 1.09 1.04 1.02 1.04 0.97 2.05 0.26 1.74 0.27 1.74 1.00
9.5 9.0 8.5 8.0 7.5 7.0 6.5 6.0 5.5 5.0 4.5 4.0 3.5 3.0 2.5 2.0 1.5 1.0 0.5 0.0 -0.5
f1 (ppm)

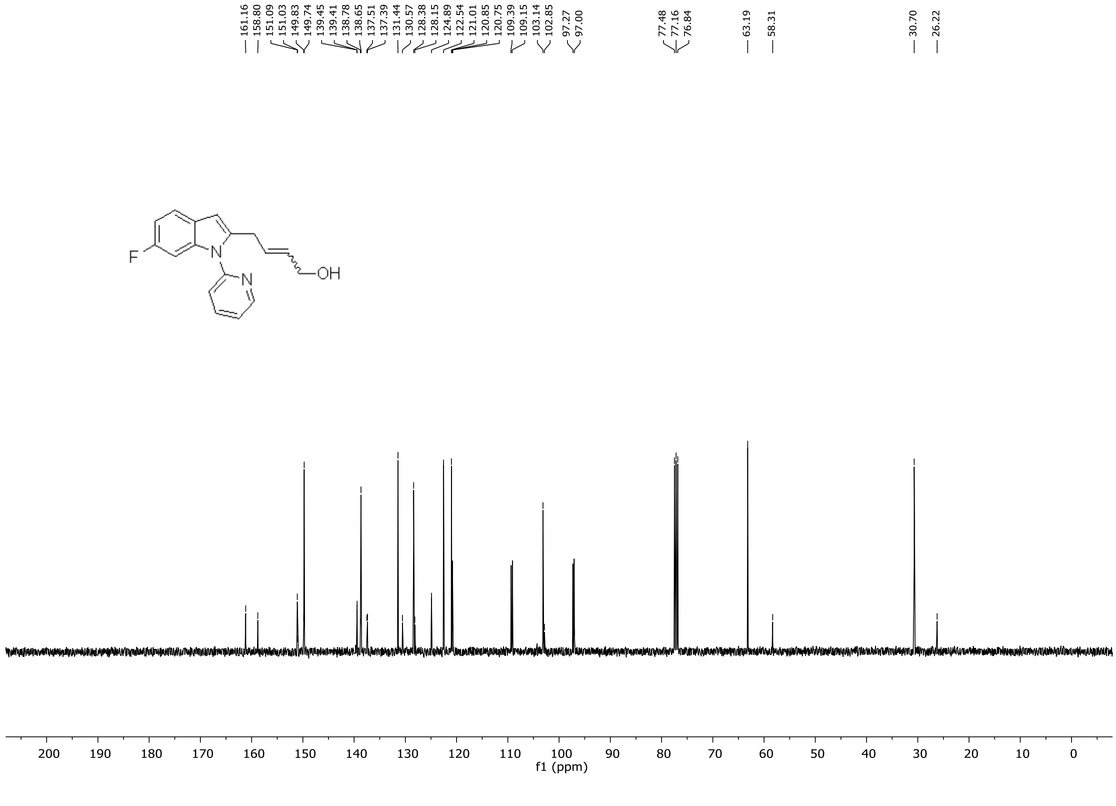

161.16 158.80 151.09 151.03 149.83 149.74 139.45 139.41 138.78 138.65 137.51 137.39 131.44 130.57 128.38 128.15 124.89 122.54 121.01 120.85 120.75 109.39 109.15 103.14 102.85 97.27 97.00 77.48 77.16 76.84 63.19 58.31 30.70 26.22
F
N
N
OH
200 190 180 170 160 150 140 130 120 110 100 90 80 70 60 50 40 30 20 10 0
f1 (ppm)

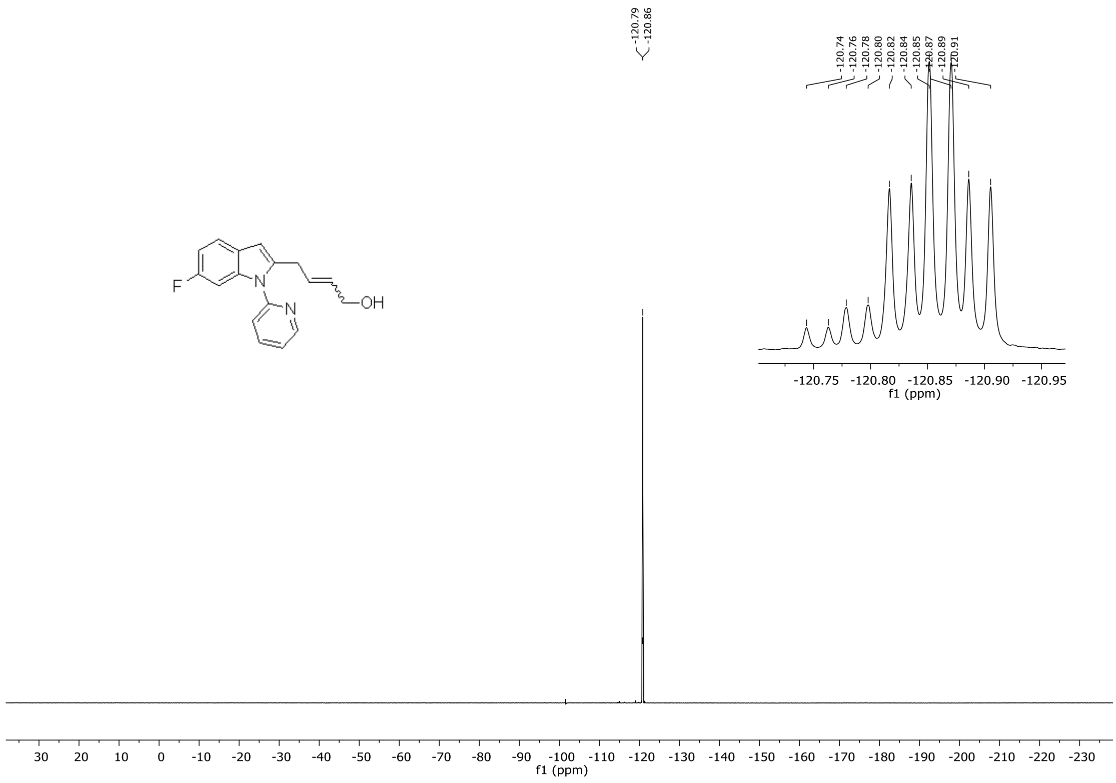
F
N
N
OH
f1 (ppm)

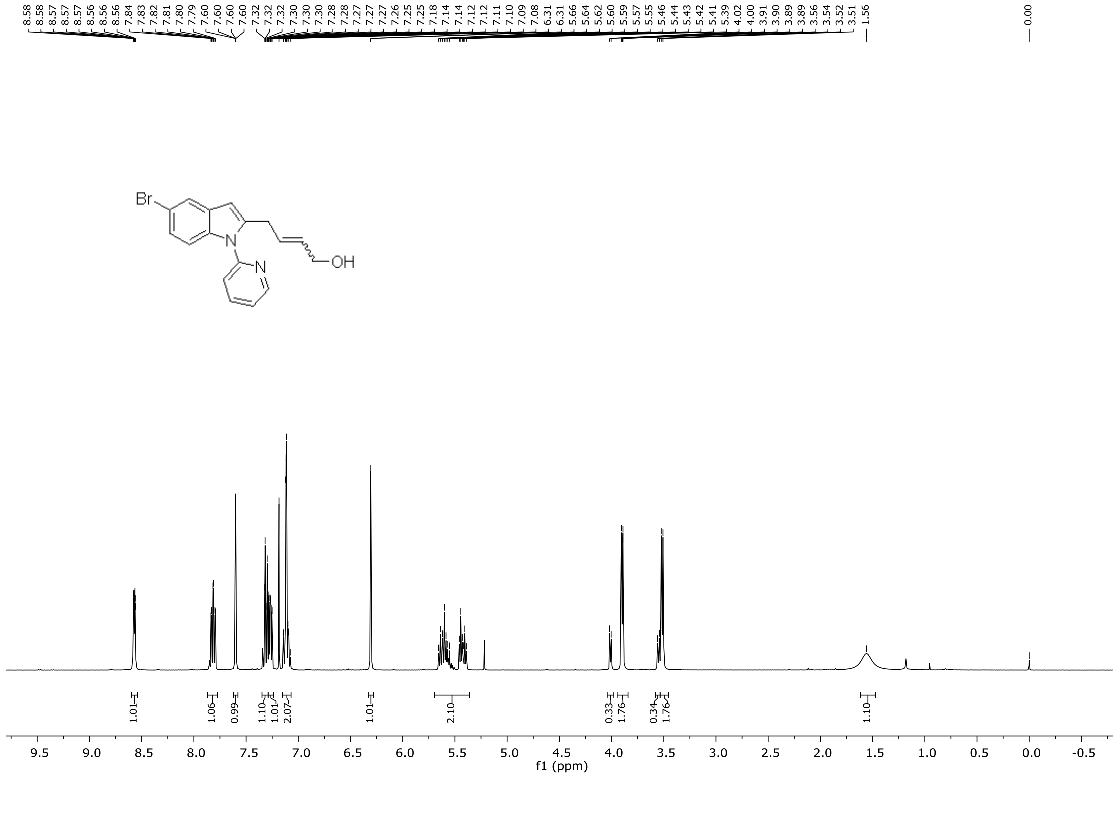
Br
N
N
OH
f1 (ppm)

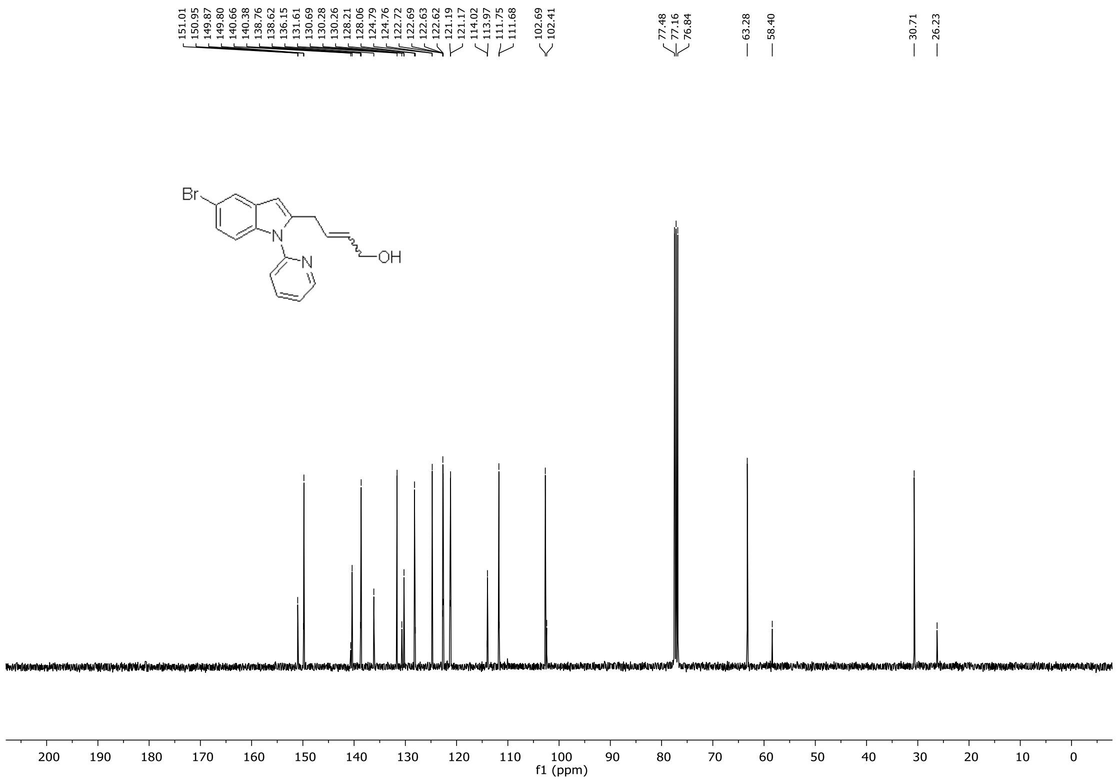
151.01
150.95
149.87
149.80
140.66
140.38
138.76
138.62
136.15
131.61
130.69
130.28
130.26
128.21
128.06
124.79
124.76
122.72
122.69
122.63
122.62
121.19
121.17
114.02
113.97
111.75
111.68
102.69
102.41
77.48
77.16
76.84
63.28
58.40
30.71
26.23
Br
N
N
OH
200 190 180 170 160 150 140 130 120 110 100 90 80 70 60 50 40 30 20 10 0
f1 (ppm)

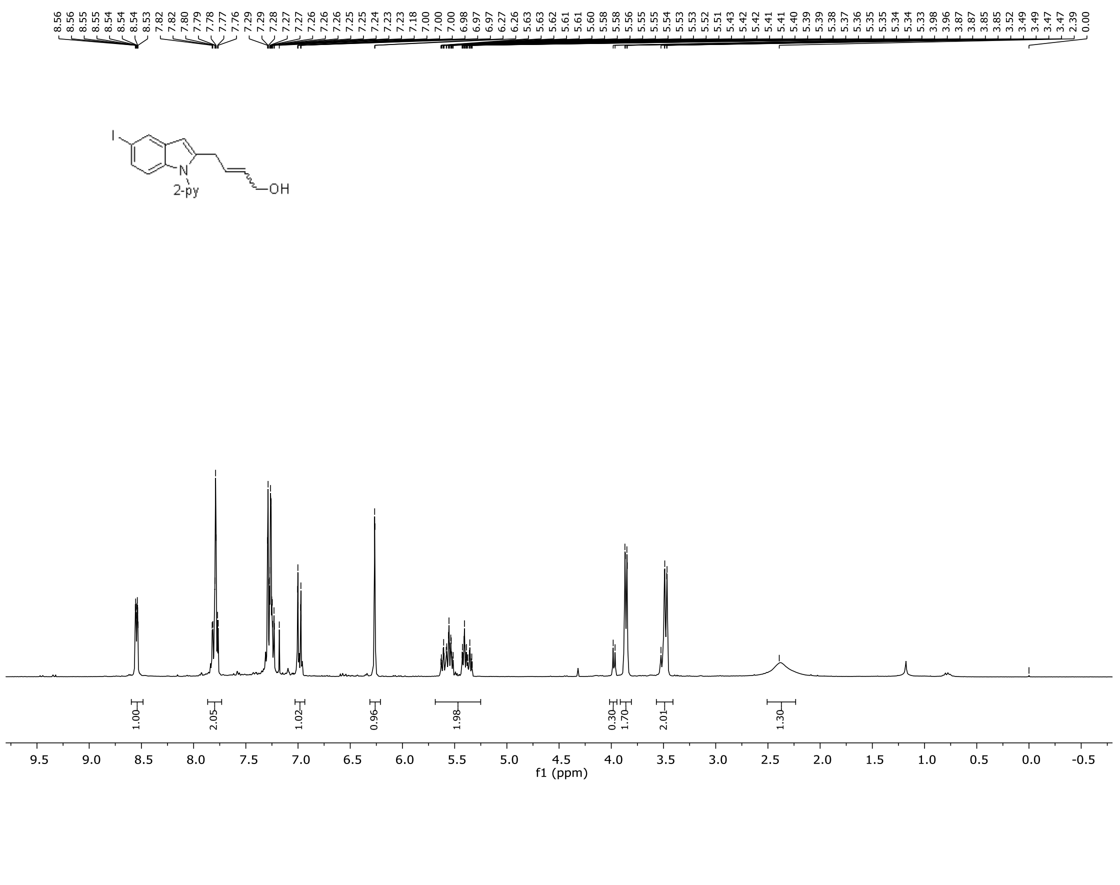
I
N
2-py
OH
1.00
2.05
1.02
0.96
1.98
0.30
1.70
2.01
1.30
9.5 9.0 8.5 8.0 7.5 7.0 6.5 6.0 5.5 5.0 4.5 4.0 3.5 3.0 2.5 2.0 1.5 1.0 0.5 0.0 -0.5
f1 (ppm)

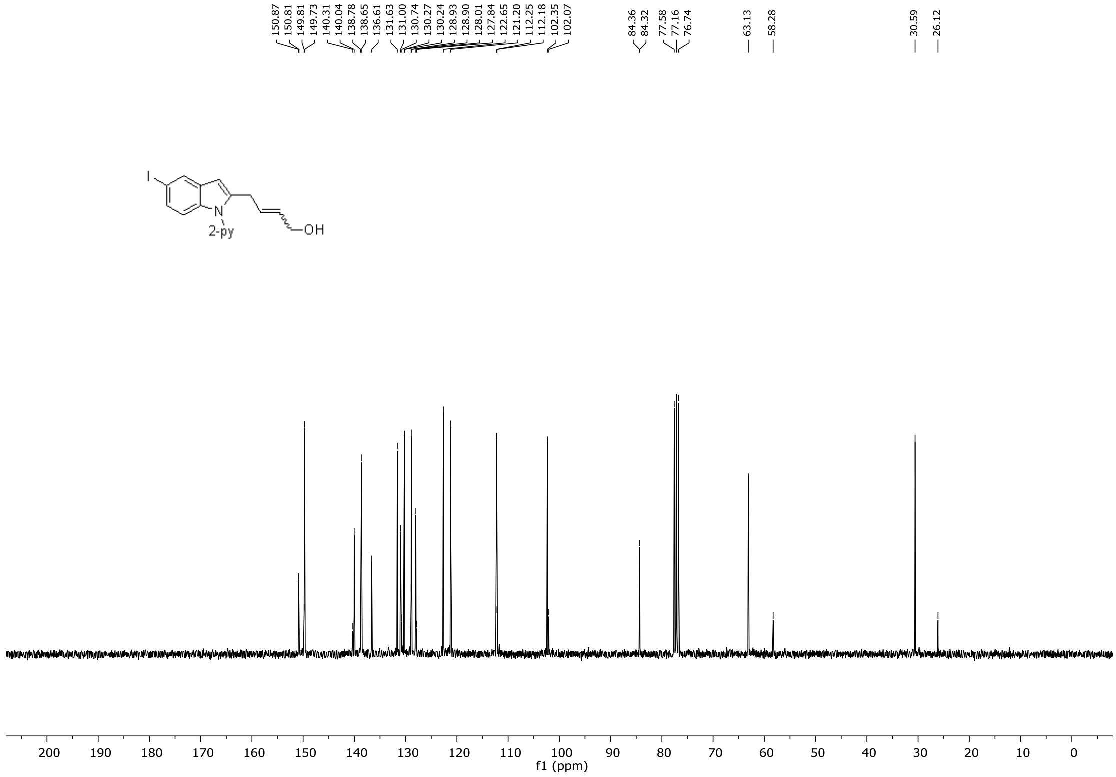
150.87
150.81
149.81
149.73
140.31
140.04
138.78
138.65
136.61
131.63
131.00
130.74
130.27
130.24
128.93
128.90
128.01
127.84
122.65
121.20
112.25
112.18
102.35
102.07
84.36
84.32
77.58
77.16
76.74
63.13
58.28
30.59
26.12
I
N
2-py
OH
200 190 180 170 160 150 140 130 120 110 100 90 80 70 60 50 40 30 20 10 0
f1 (ppm)

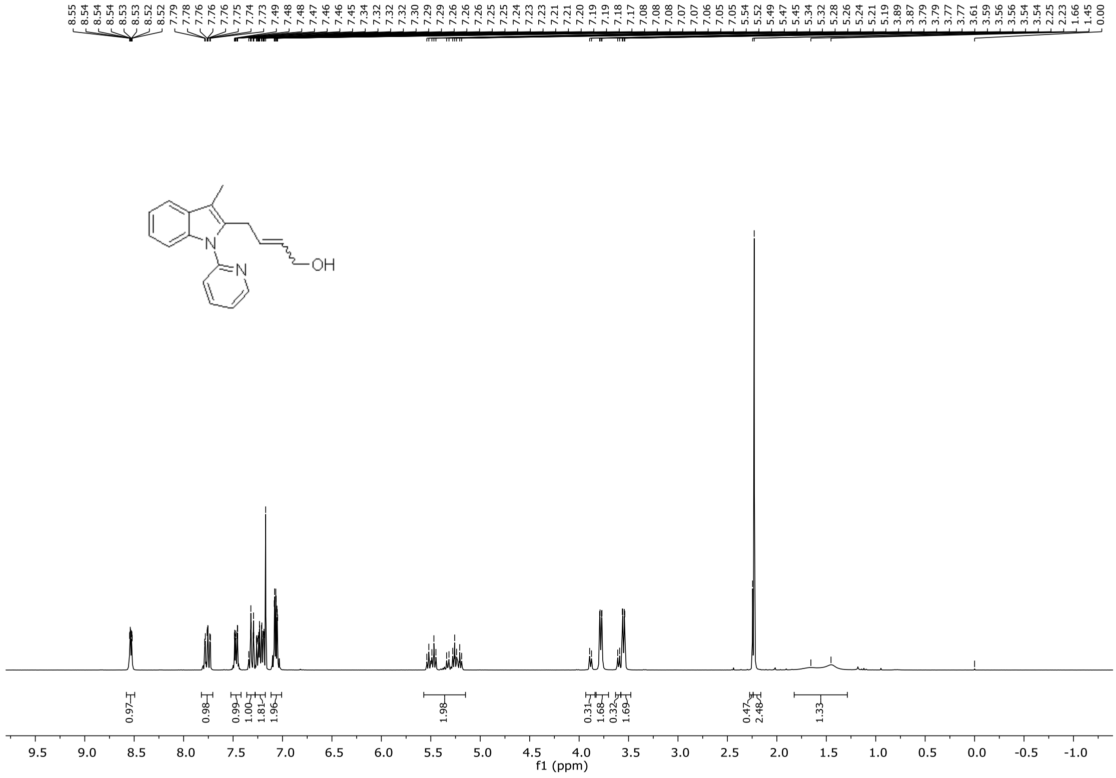
8.55
8.54
8.54
8.54
8.53
8.53
8.52
8.52
7.79
7.78
7.76
7.76
7.75
7.74
7.73
7.49
7.48
7.47
7.46
7.46
7.45
7.34
7.32
7.32
7.32
7.30
7.29
7.29
7.26
7.26
7.26
7.25
7.24
7.23
7.23
7.21
7.21
7.20
7.19
7.19
7.18
7.17
7.08
7.08
7.08
7.07
7.07
7.06
7.05
7.05
5.54
5.52
5.49
5.47
5.45
5.34
5.32
5.28
5.26
5.24
5.21
5.19
3.89
3.87
3.79
3.79
3.77
3.77
3.61
3.59
3.56
3.56
3.54
3.54
2.25
2.23
1.66
1.45
0.00
N
N
OH
0.97
0.98
0.99
1.00
1.81
1.96
1.98
0.31
1.68
0.32
1.69
0.47
2.48
1.33
9.5 9.0 8.5 8.0 7.5 7.0 6.5 6.0 5.5 5.0 4.5 4.0 3.5 3.0 2.5 2.0 1.5 1.0 0.5 0.0 -0.5 -1.0
f1 (ppm)

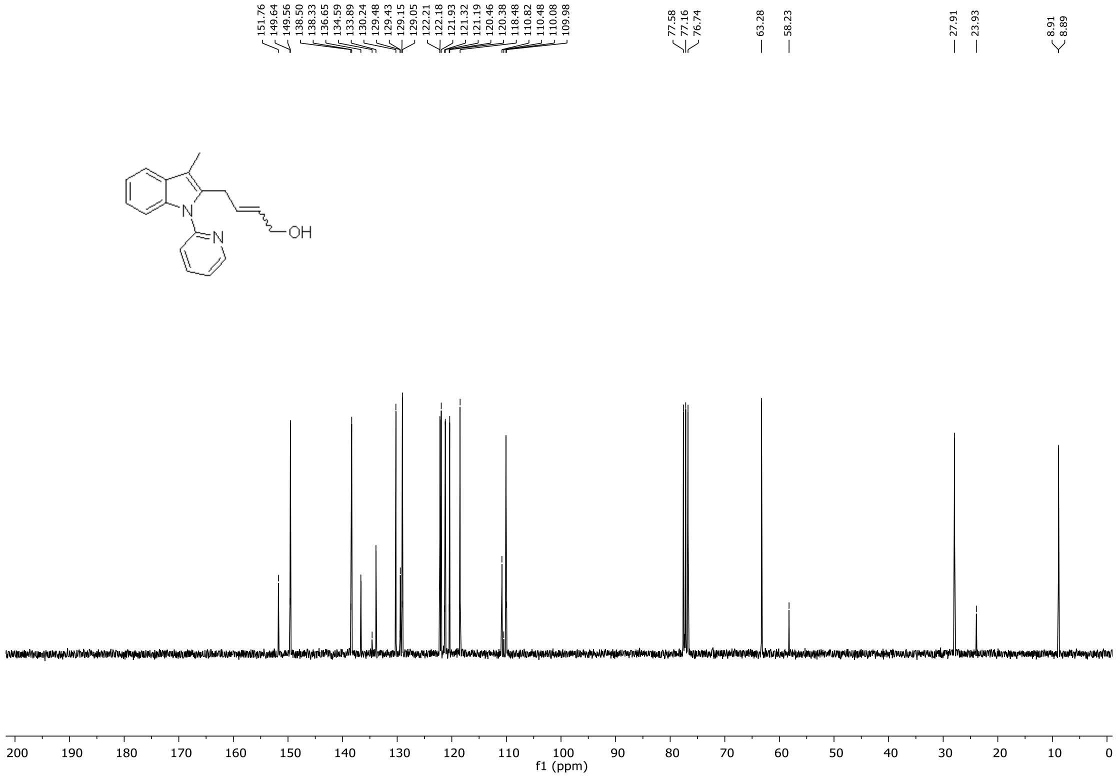
OH
N
f1 (ppm)

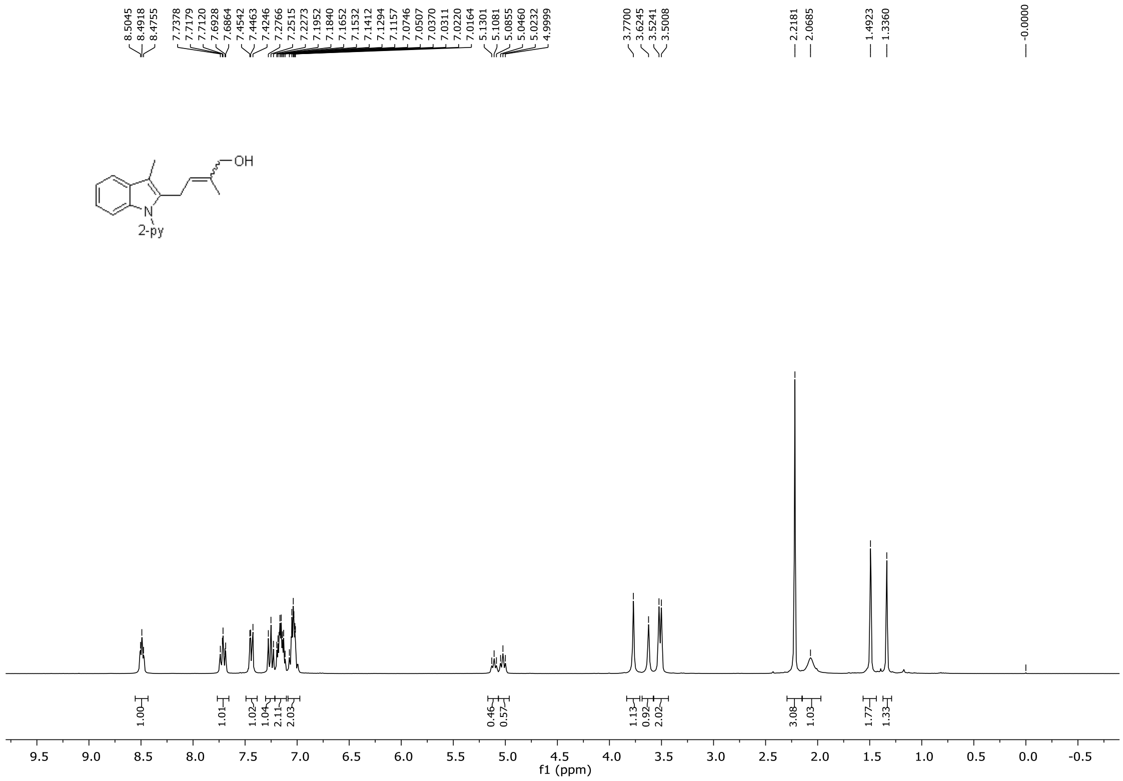
OH
N
2-py
f1 (ppm)

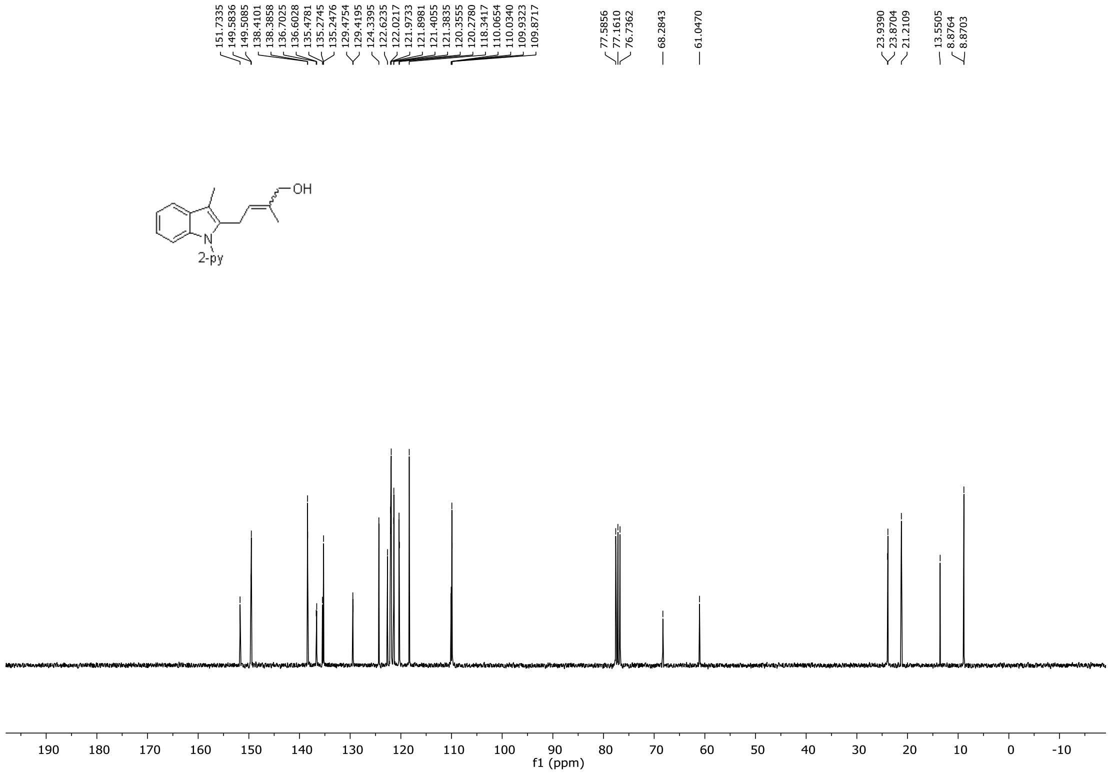
151.7335
149.5836
149.5085
138.4101
138.3858
136.7025
136.6028
135.4781
135.2745
135.2476
129.4754
129.4195
124.3395
122.6235
122.0217
121.9733
121.8981
121.4055
121.3835
120.3555
120.2780
118.3417
110.0654
110.0340
109.9323
109.8717
77.5856
77.1610
76.7362
68.2843
61.0470
23.9390
23.8704
21.2109
13.5505
8.8764
8.8703
OH
N
2-py
190 180 170 160 150 140 130 120 110 100 90 80 70 60 50 40 30 20 10 0 -10
f1 (ppm)

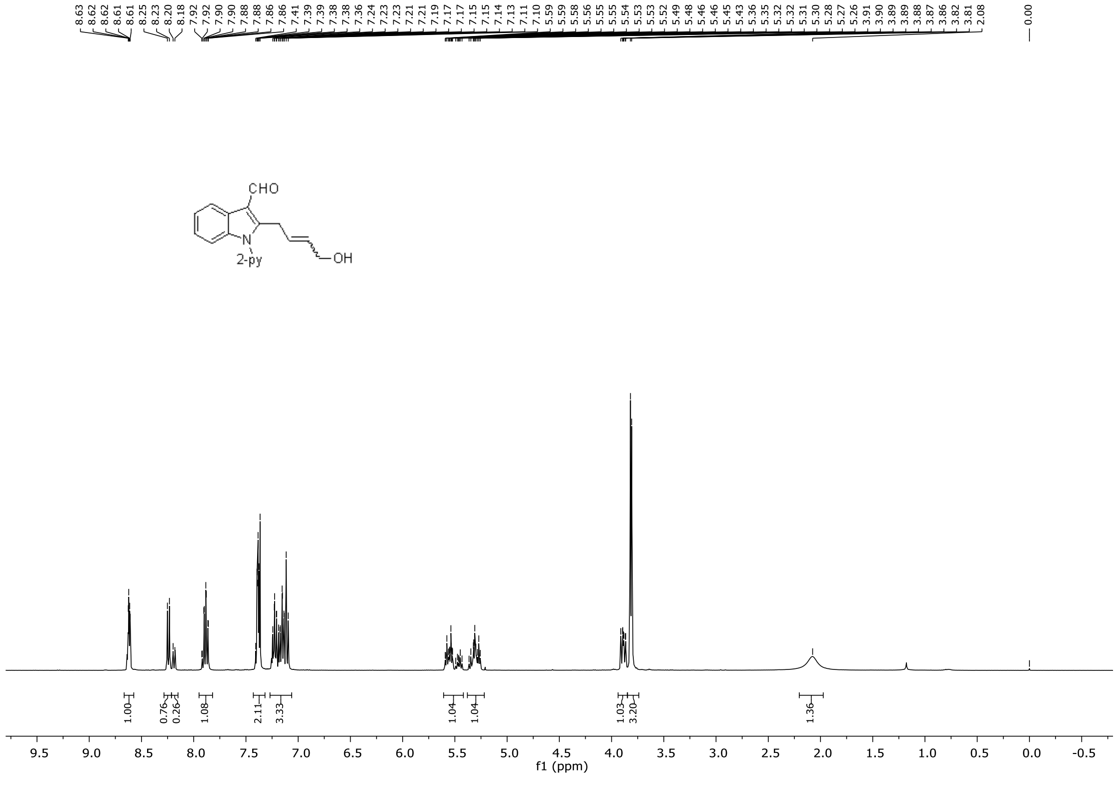
8.63
8.62
8.62
8.61
8.61
8.25
8.23
8.20
8.18
7.92
7.92
7.90
7.90
7.88
7.86
7.86
7.41
7.39
7.39
7.38
7.38
7.36
7.24
7.23
7.23
7.21
7.21
7.19
7.17
7.17
7.15
7.15
7.14
7.13
7.11
7.10
5.59
5.59
5.58
5.56
5.55
5.55
5.54
5.53
5.53
5.52
5.49
5.48
5.46
5.46
5.45
5.43
5.36
5.35
5.32
5.31
5.30
5.28
5.27
5.26
3.91
3.90
3.89
3.88
3.87
3.86
3.82
3.81
2.08
0.00
CHO
N
2-py
OH
1.00
0.76
0.26
1.08
2.11
3.33
1.04
1.04
1.03
3.20
1.36
9.5 9.0 8.5 8.0 7.5 7.0 6.5 6.0 5.5 5.0 4.5 4.0 3.5 3.0 2.5 2.0 1.5 1.0 0.5 0.0 -0.5
f1 (ppm)

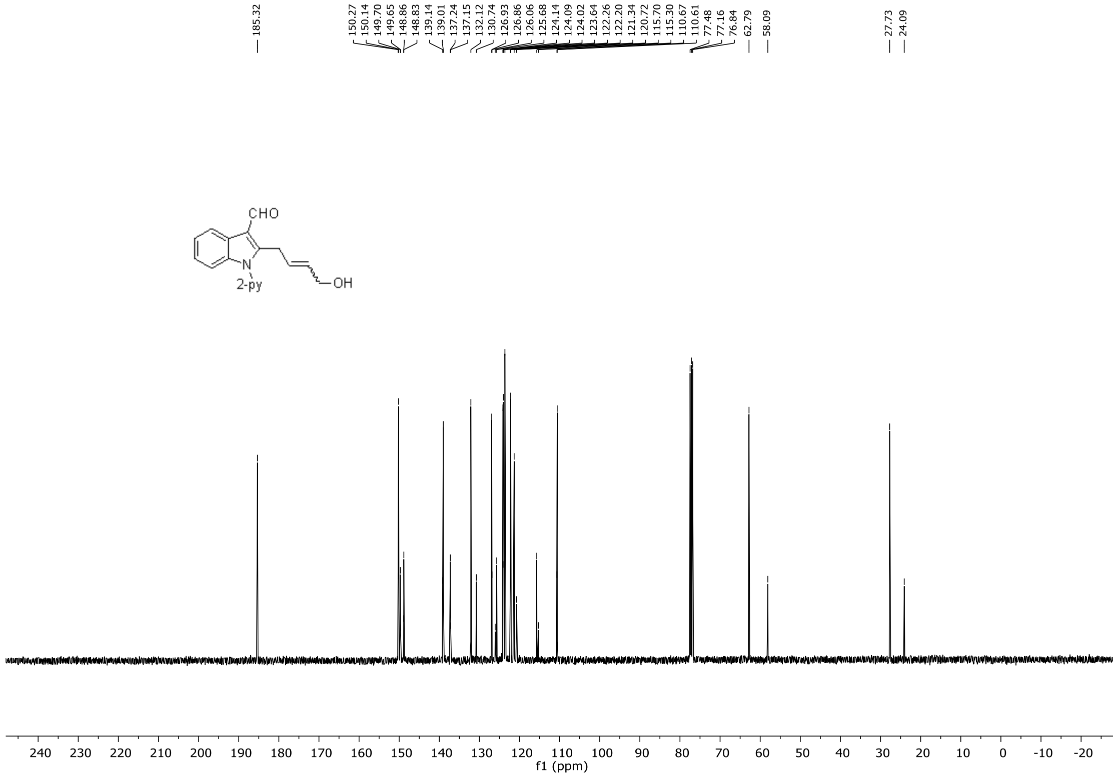

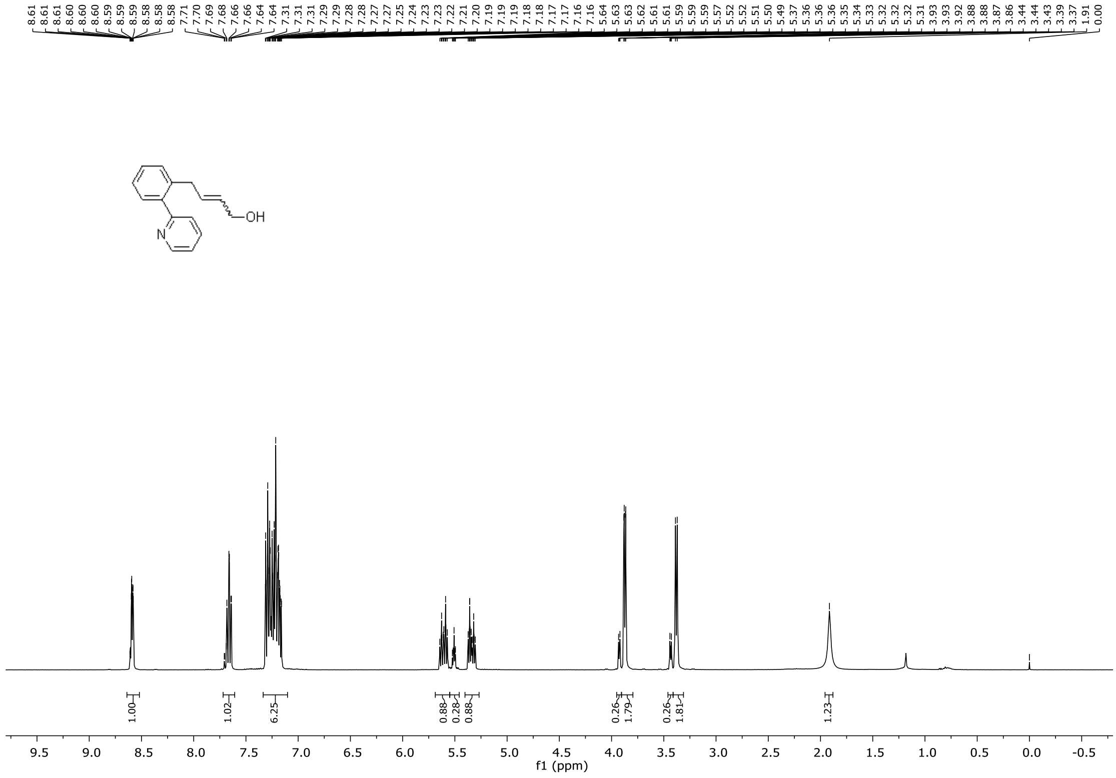

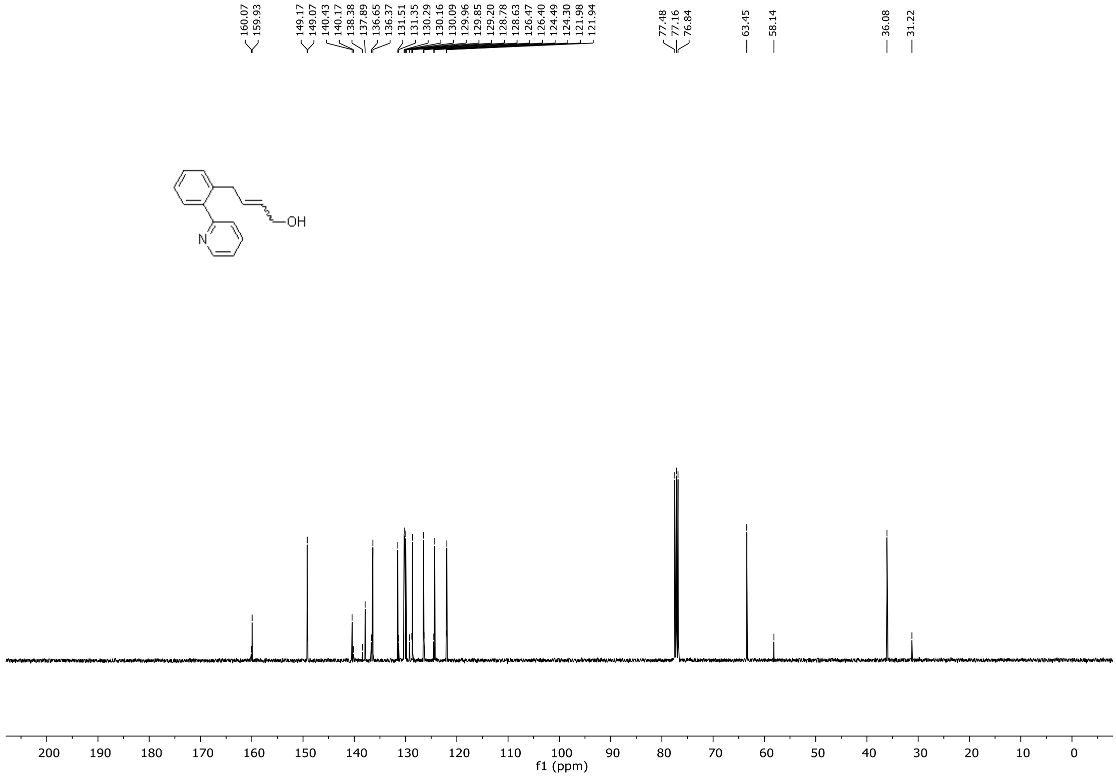
160.07
159.93
149.17
149.07
140.43
140.17
138.38
137.89
136.65
136.37
131.51
131.35
130.29
130.16
130.09
129.96
129.85
129.20
128.78
128.63
126.47
126.40
124.49
124.30
121.98
121.94
77.48
77.16
76.84
63.45
58.14
36.08
31.22
OH
N
f1 (ppm)

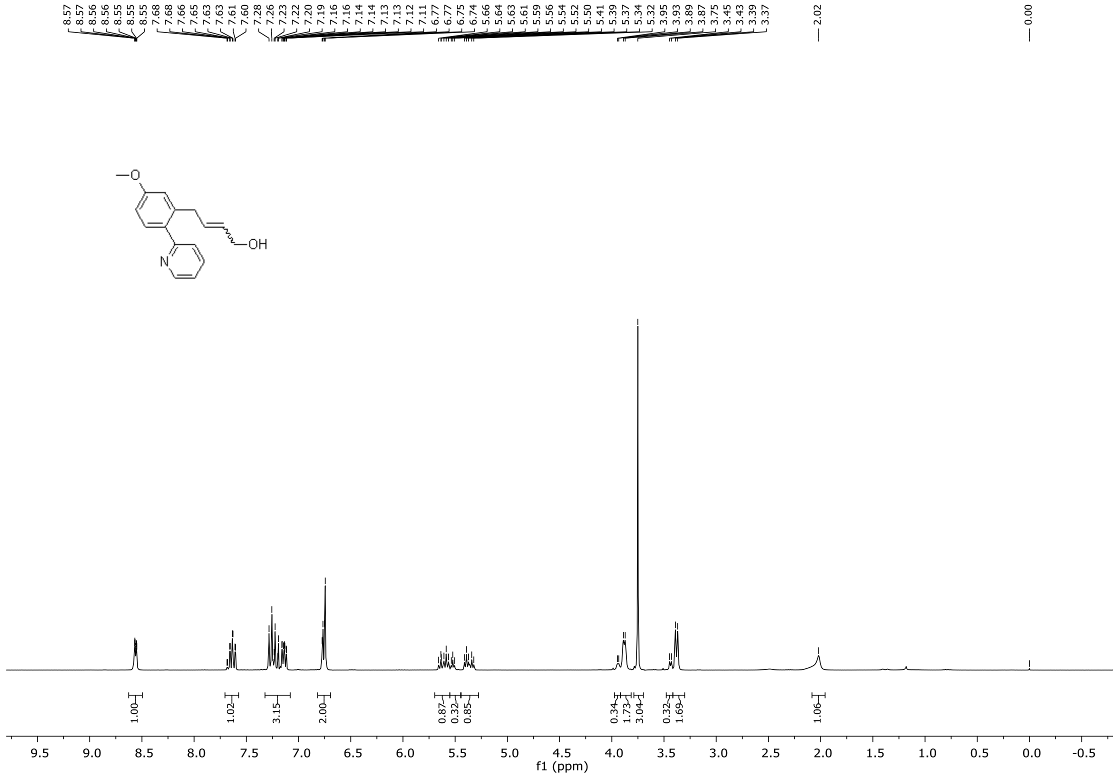
8.57
8.57
8.56
8.55
8.55
8.55
7.68
7.66
7.65
7.63
7.63
7.61
7.60
7.28
7.26
7.23
7.22
7.20
7.19
7.16
7.16
7.14
7.14
7.13
7.13
7.12
7.11
6.77
6.75
6.74
5.66
5.64
5.63
5.61
5.59
5.56
5.54
5.52
5.50
5.41
5.39
5.37
5.34
5.32
3.95
3.93
3.89
3.87
3.75
3.45
3.43
3.39
3.37
2.02
0.00
O
OH
N
1.00
1.02
3.15
2.00
0.87
0.32
0.85
0.34
1.73
3.04
0.32
1.69
1.06
f1 (ppm)

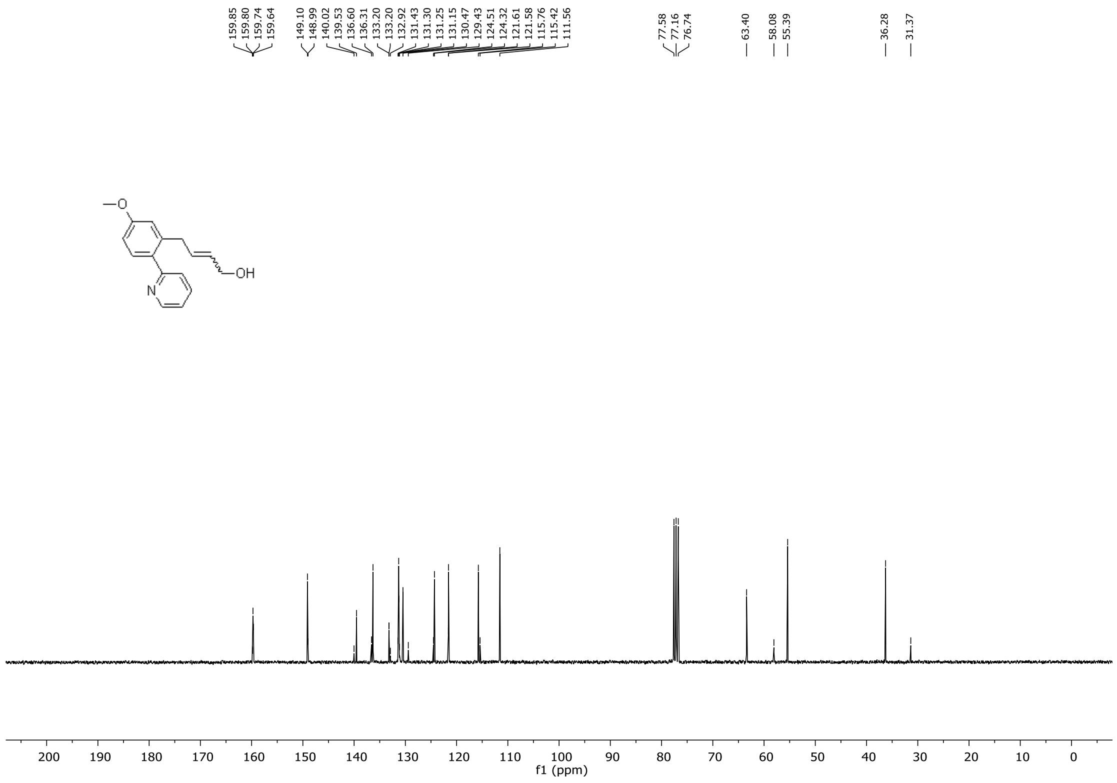
OH
N
f1 (ppm)

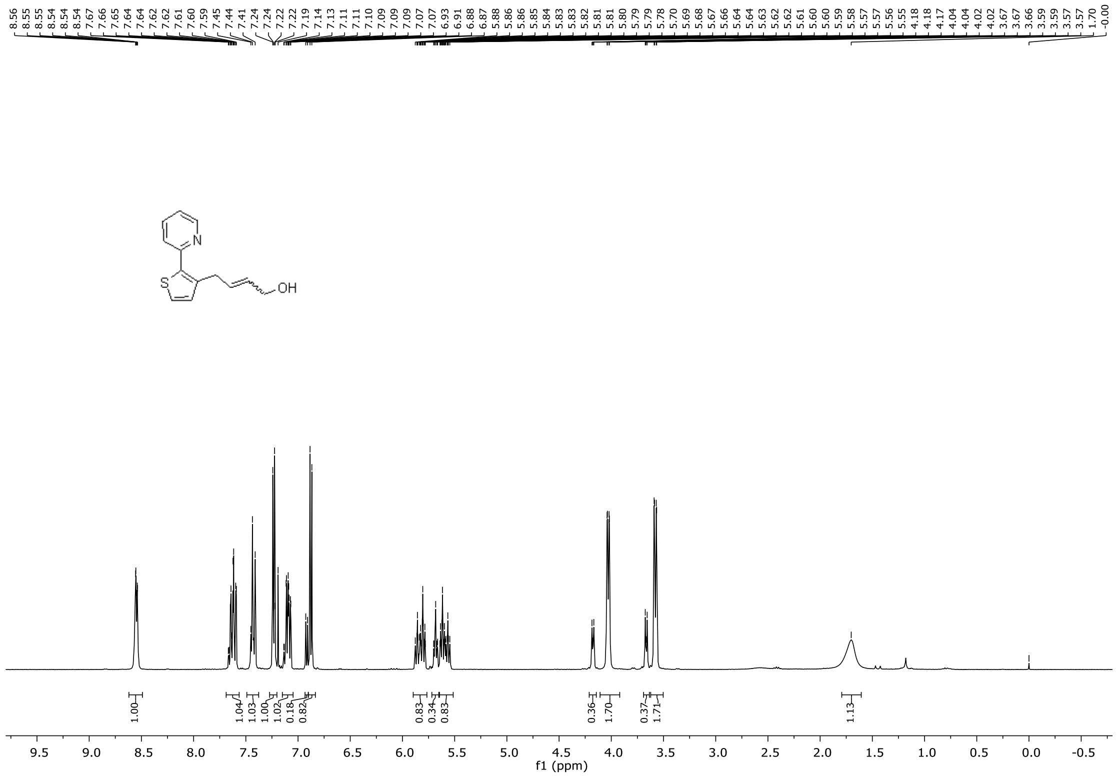
N
S
OH
f1 (ppm)

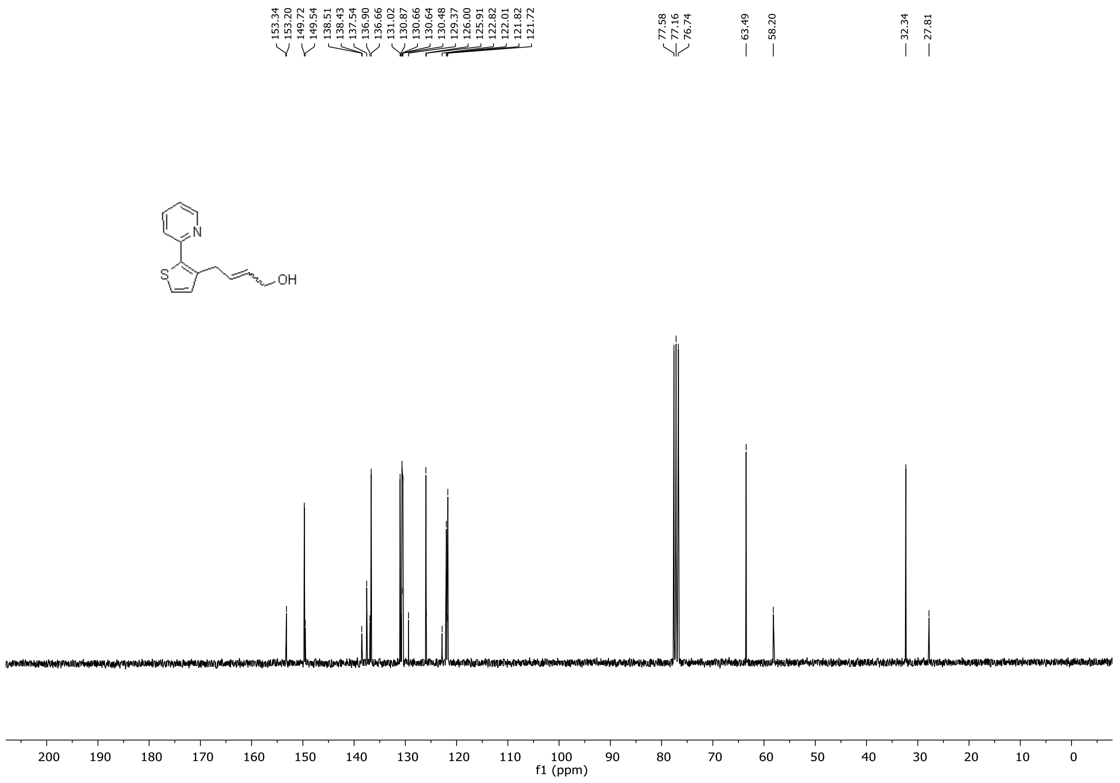

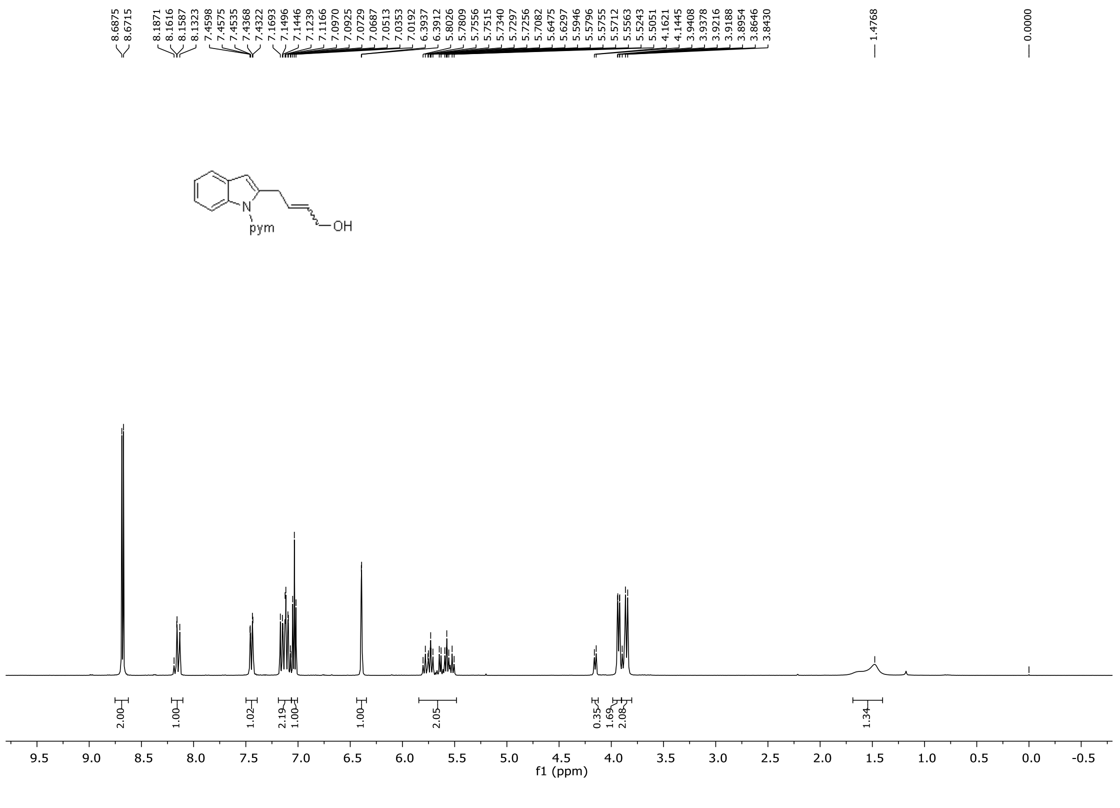

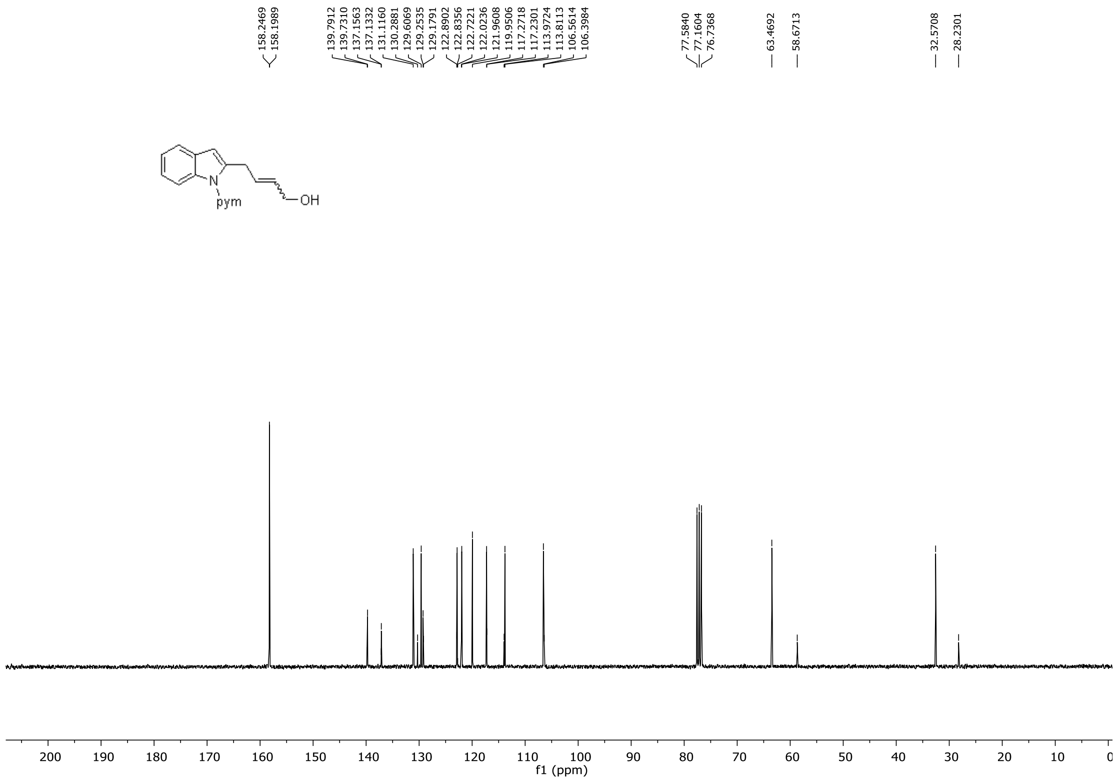
158.2469
158.1989
139.7912
139.7310
137.1563
137.1332
131.1160
130.2881
129.6069
129.2535
129.1791
122.8902
122.8356
122.7221
122.0236
121.9608
119.9506
117.2718
117.2301
113.9724
113.8113
106.5614
106.3984
77.5840
77.1604
76.7368
63.4692
58.6713
32.5708
28.2301
N
pym
OH
f1 (ppm)

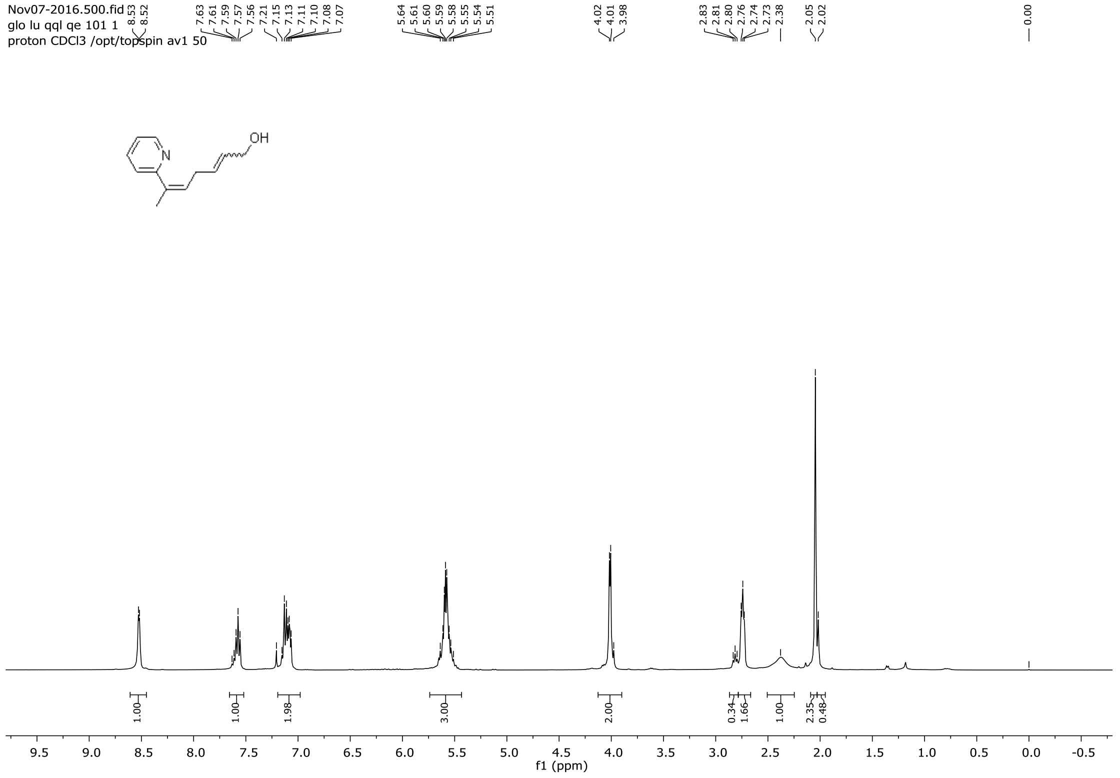
Nov07-2016.500.fid
glo lu qql qe 101 1
proton CDCl3 /opt/topspin av1 50
8.53
8.52
7.63
7.61
7.59
7.57
7.56
7.21
7.15
7.13
7.11
7.10
7.08
7.07
5.64
5.61
5.60
5.59
5.58
5.55
5.54
5.51
4.02
4.01
3.98
2.83
2.81
2.80
2.76
2.74
2.73
2.38
2.05
2.02
0.00
N
OH
1.00
1.00
1.98
3.00
2.00
0.34
1.66
1.00
2.35
0.48
f1 (ppm)

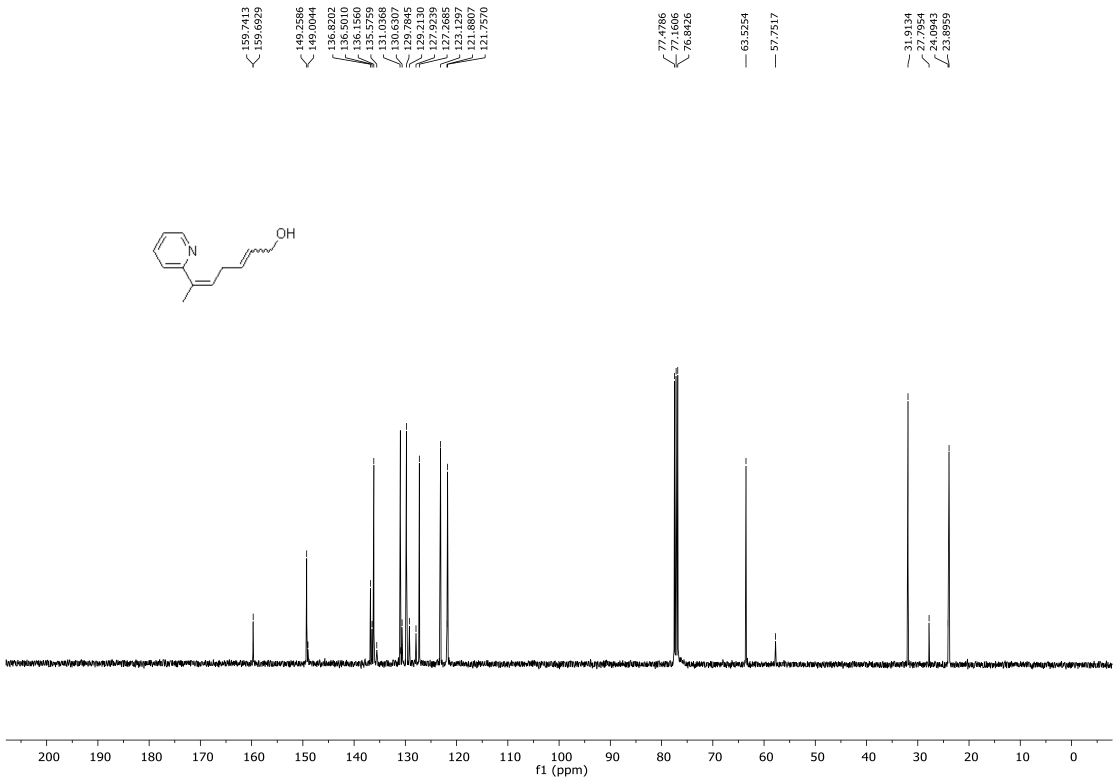
159.7413
159.6929
149.2586
149.0044
136.8202
136.5010
136.1560
135.5759
131.0368
130.6307
129.7845
129.2130
127.9239
127.2685
123.1297
121.8807
121.7570
77.4786
77.1606
76.8426
63.5254
57.7517
31.9134
27.7954
24.0943
23.8959
N
OH
f1 (ppm)

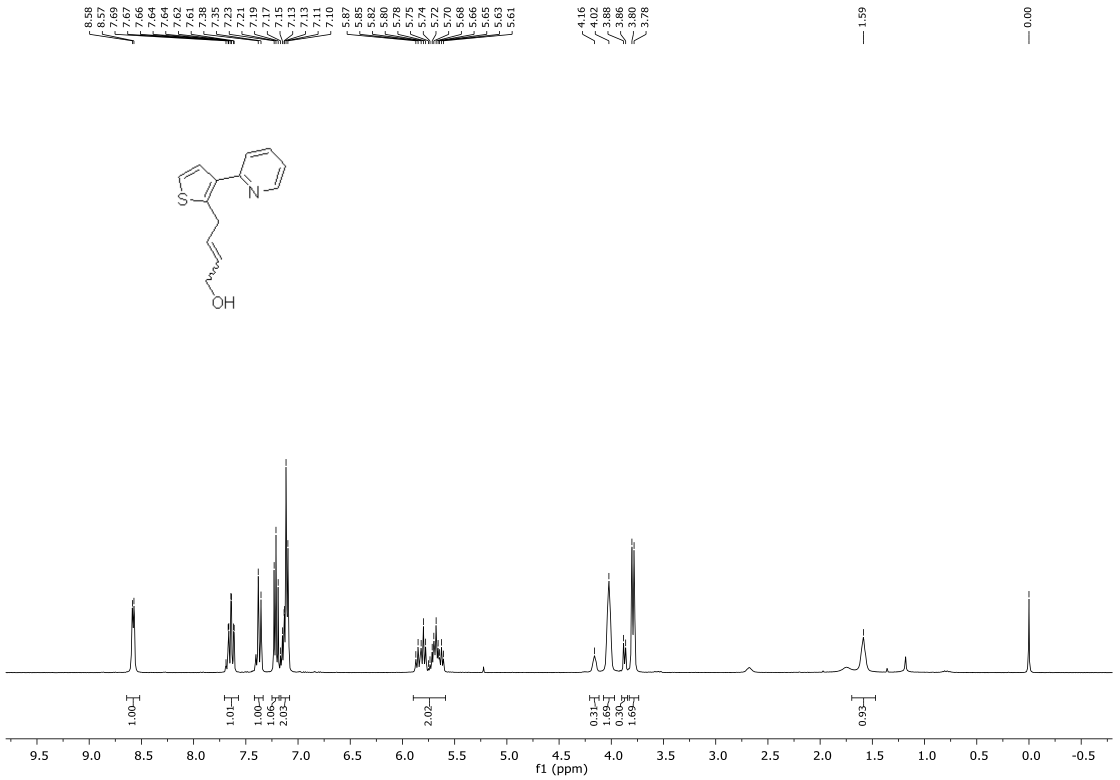
8.58
8.57
7.69
7.67
7.66
7.64
7.64
7.62
7.61
7.38
7.35
7.23
7.21
7.19
7.17
7.15
7.13
7.13
7.11
7.10
5.87
5.85
5.82
5.80
5.78
5.75
5.74
5.72
5.70
5.68
5.66
5.65
5.63
5.61
4.16
4.02
3.88
3.86
3.80
3.78
1.59
0.00
S
N
OH
1.00
1.01
1.00
1.06
2.03
2.02
0.31
1.69
0.30
1.69
0.93
f1 (ppm)

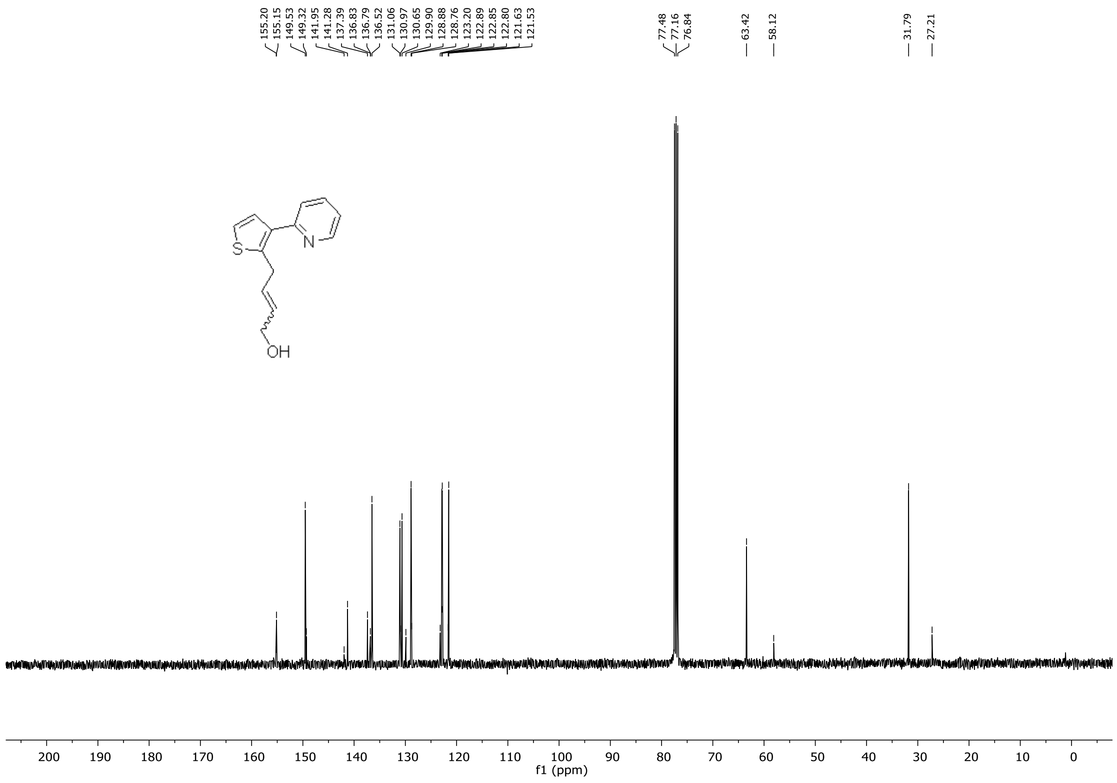
S
N
OH
f1 (ppm)

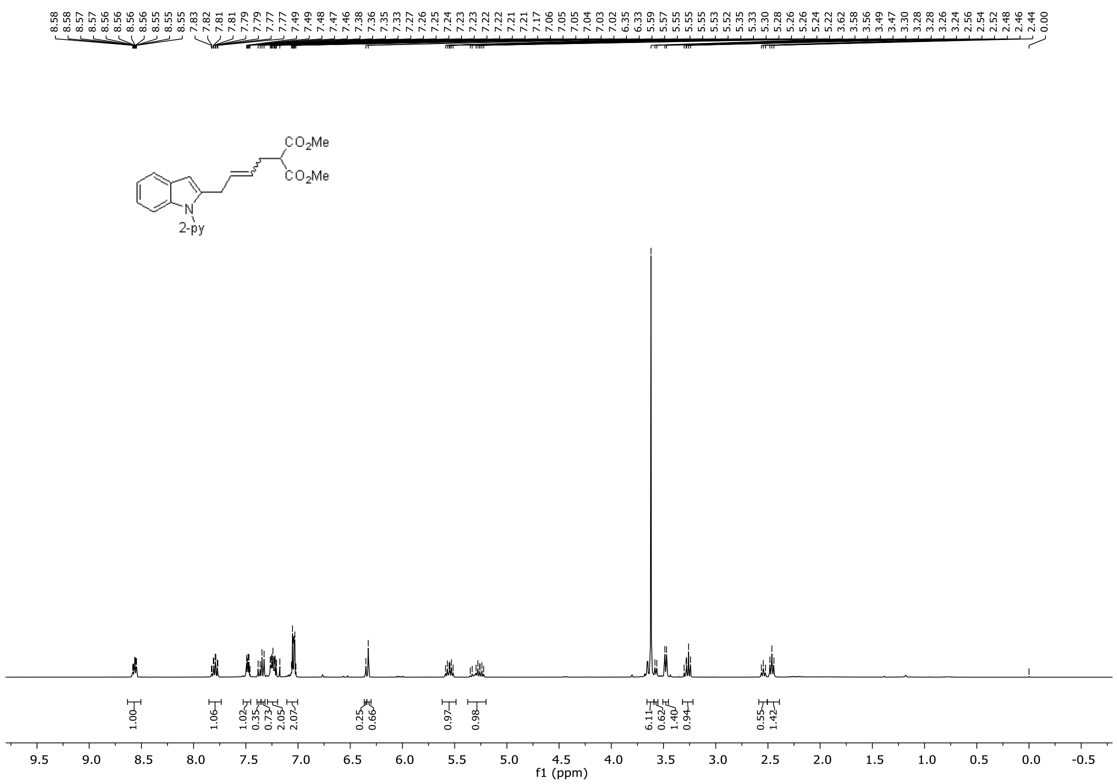
CO2Me
CO2Me
N
2-py
f1 (ppm)

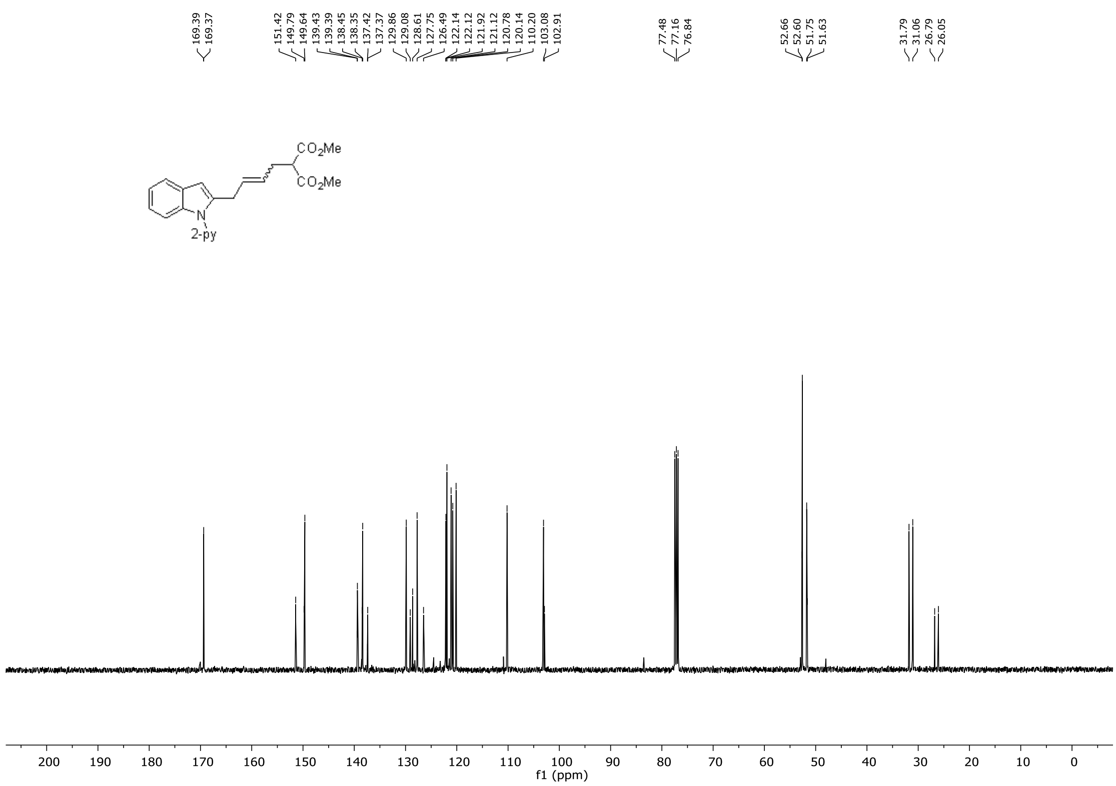
169.39
169.37
151.42
149.79
149.64
139.43
139.39
138.45
138.35
137.42
137.37
129.86
129.08
128.61
127.75
126.49
122.14
122.12
121.92
121.12
120.78
120.14
110.20
103.08
102.91
77.48
77.16
76.84
52.66
52.60
51.75
51.63
31.79
31.06
26.79
26.05
CO2Me
CO2Me
N
2-py
f1 (ppm)

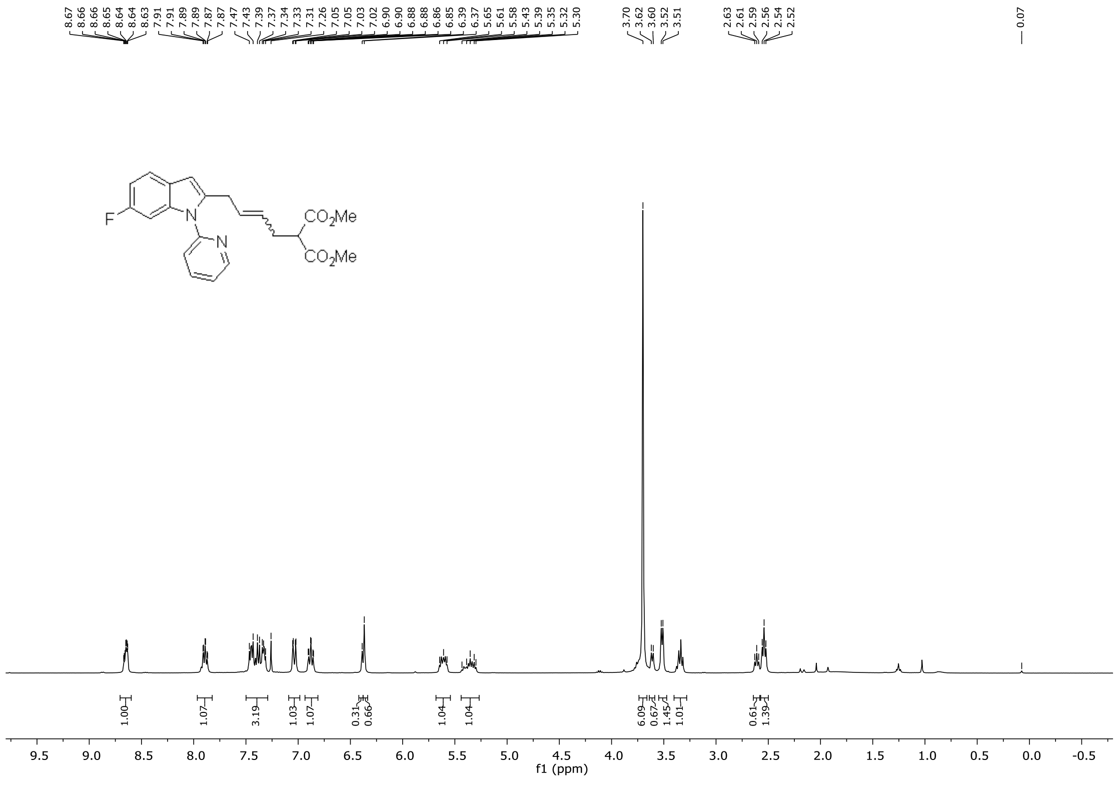
8.67
8.66
8.66
8.65
8.64
8.63
7.91
7.91
7.89
7.89
7.87
7.87
7.47
7.43
7.39
7.37
7.34
7.33
7.31
7.26
7.05
7.03
7.02
6.90
6.88
6.88
6.86
6.85
6.39
6.37
5.65
5.61
5.58
5.43
5.39
5.35
5.32
5.30
3.70
3.62
3.60
3.52
3.51
2.63
2.61
2.59
2.56
2.54
2.52
0.07
F
N
N
CO2Me
CO2Me
1.00
1.07
3.19
1.03
1.07
0.31
0.66
1.04
1.04
6.09
0.67
1.45
1.01
0.61
1.39
f1 (ppm)

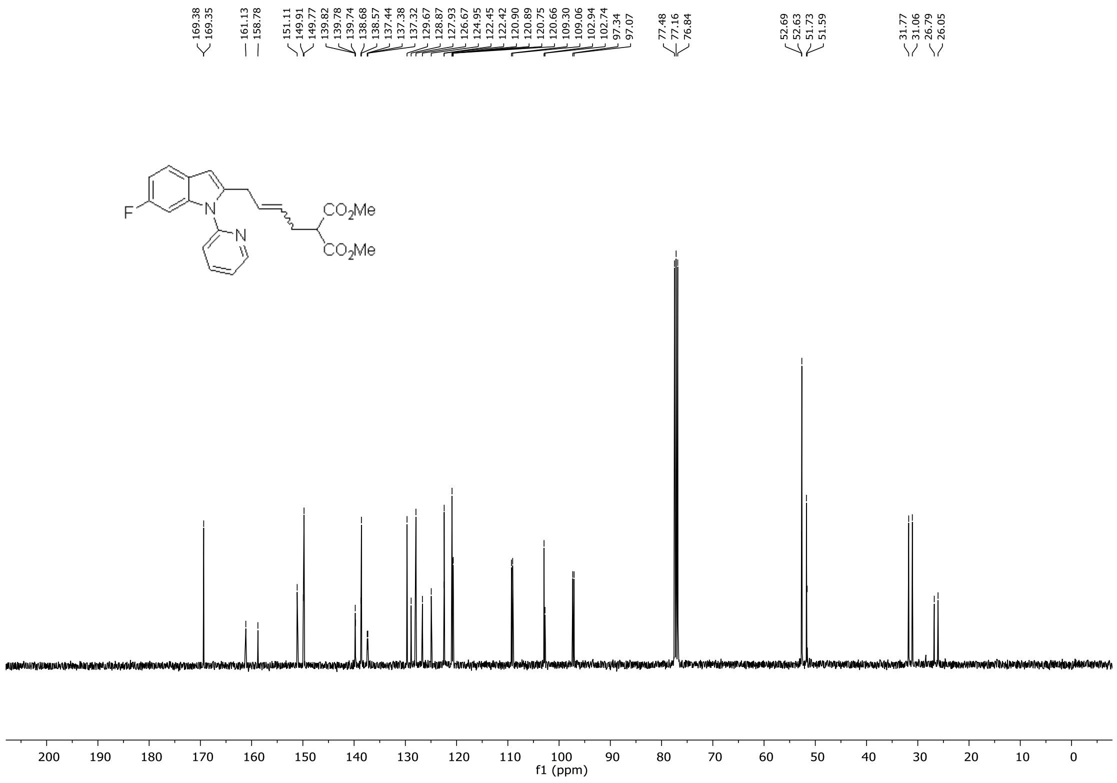
169.38
169.35
161.13
158.78
151.11
149.91
149.77
139.82
139.78
139.74
138.68
138.57
137.44
137.38
137.32
129.67
128.87
127.93
126.67
124.95
122.45
122.42
120.90
120.89
120.75
120.66
109.30
109.06
102.94
102.74
97.34
97.07
77.48
77.16
76.84
52.69
52.63
51.73
51.59
31.77
31.06
26.79
26.05
F
N
N
CO2Me
CO2Me
f1 (ppm)

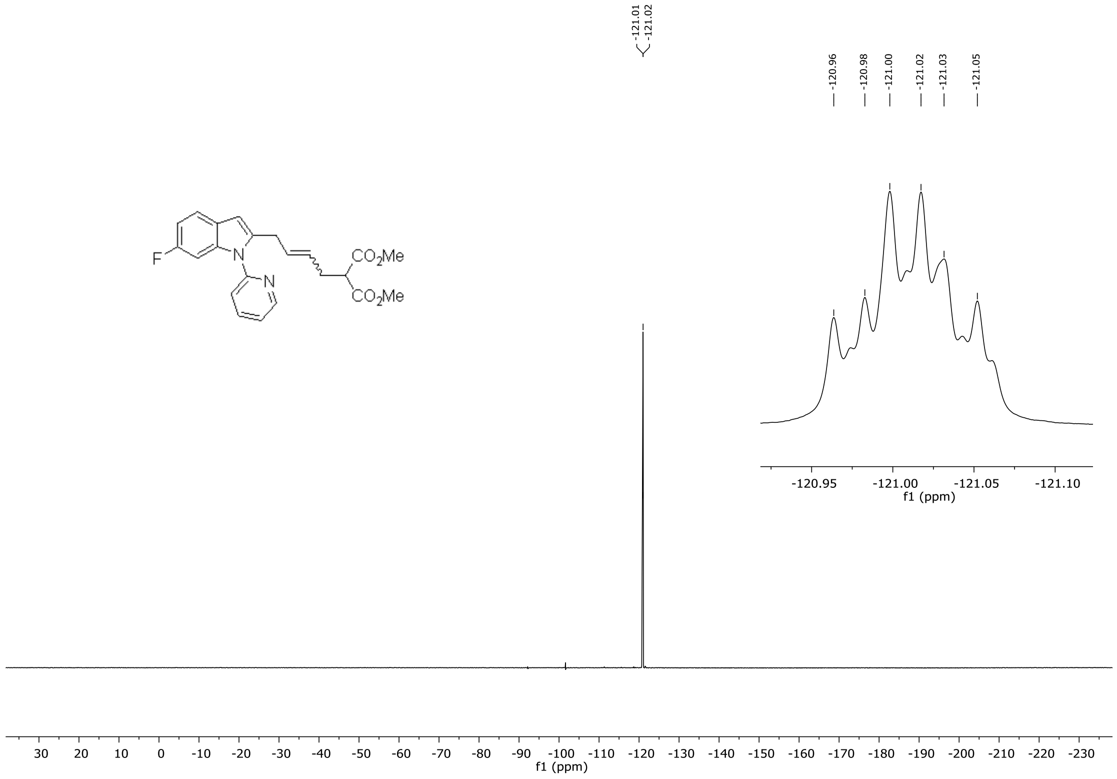
-121.01
-121.02
-120.96
-120.98
-121.00
-121.02
-121.03
-121.05
F
N
N
CO2Me
CO2Me
f1 (ppm)
f1 (ppm)

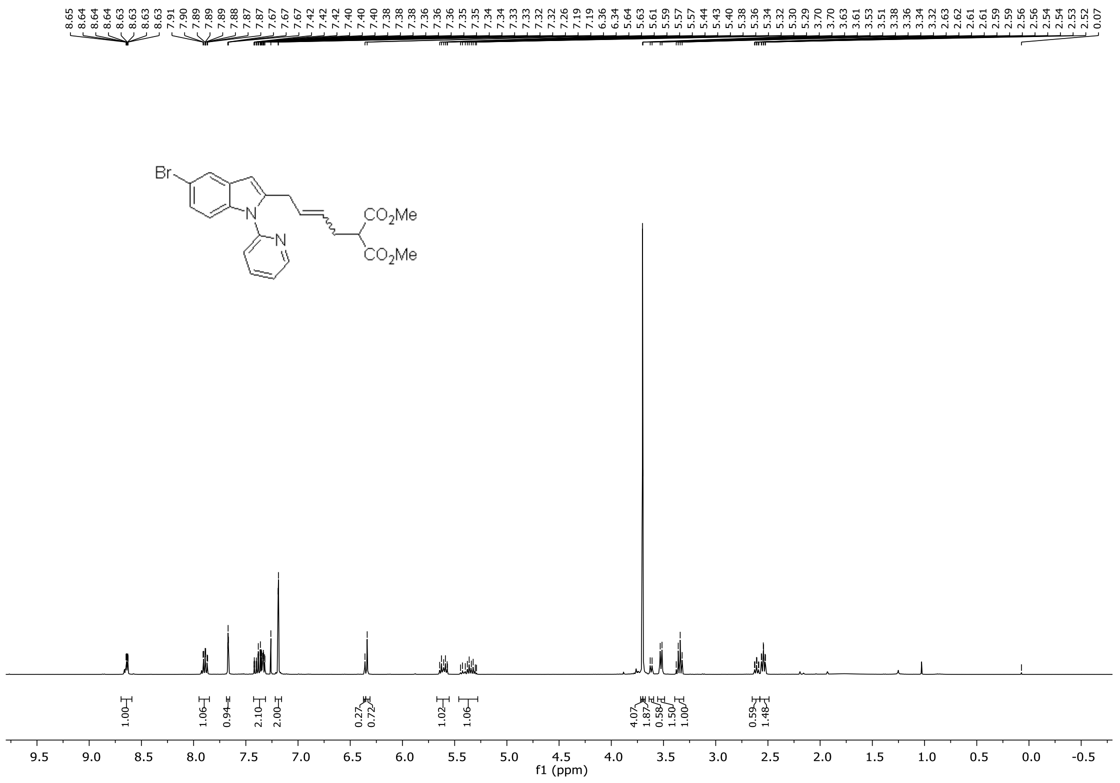
8.65 8.64 8.64 8.63 8.63 8.63 8.63 7.91 7.90 7.89 7.89 7.88 7.87 7.87 7.67 7.67 7.67 7.42 7.42 7.40 7.40 7.38 7.38 7.38 7.36 7.36 7.35 7.35 7.34 7.34 7.33 7.33 7.32 7.32 7.26 7.19 7.19 6.36 6.34 5.64 5.63 5.61 5.59 5.57 5.57 5.44 5.43 5.40 5.38 5.36 5.34 5.32 5.30 5.29 3.70 3.63 3.61 3.53 3.51 3.38 3.36 3.34 3.32 2.63 2.62 2.61 2.61 2.59 2.59 2.56 2.54 2.54 2.53 2.52 0.07
Br
N
N
CO2Me
CO2Me
1.00 1.06 0.94 2.10 2.00 0.27 0.72 1.02 1.06 4.07 1.87 0.58 1.50 1.00 0.59 1.48
9.5 9.0 8.5 8.0 7.5 7.0 6.5 6.0 5.5 5.0 4.5 4.0 3.5 3.0 2.5 2.0 1.5 1.0 0.5 0.0 -0.5
f1 (ppm)

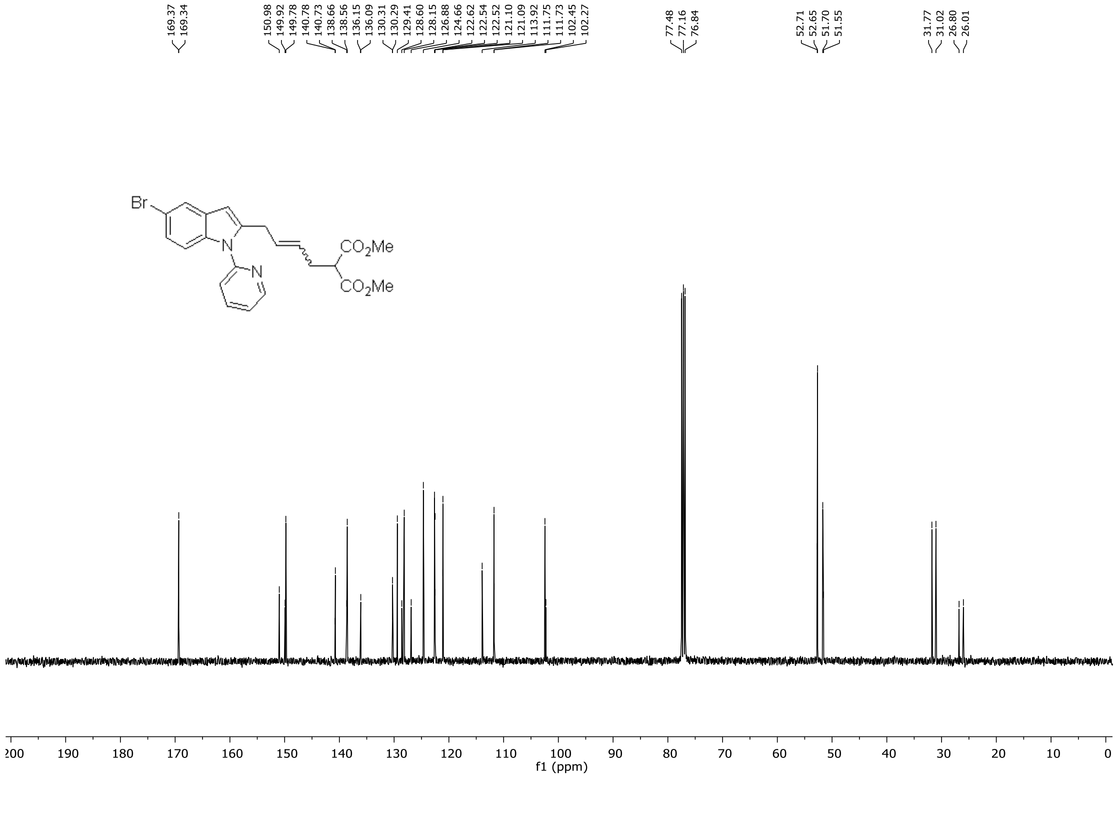
169.37 169.34
150.98 149.92 149.78 140.78 140.73 138.66 138.56 136.15 136.09 130.31 130.29 129.41 128.60 128.15 126.88 124.66 122.62 122.54 122.52 121.10 121.09 113.92 111.75 111.73 102.45 102.27
77.48 77.16 76.84
52.71 52.65 51.70 51.55
31.77 31.02 26.80 26.01
Br
N
N
CO2Me
CO2Me
200 190 180 170 160 150 140 130 120 110 100 90 80 70 60 50 40 30 20 10 0
f1 (ppm)

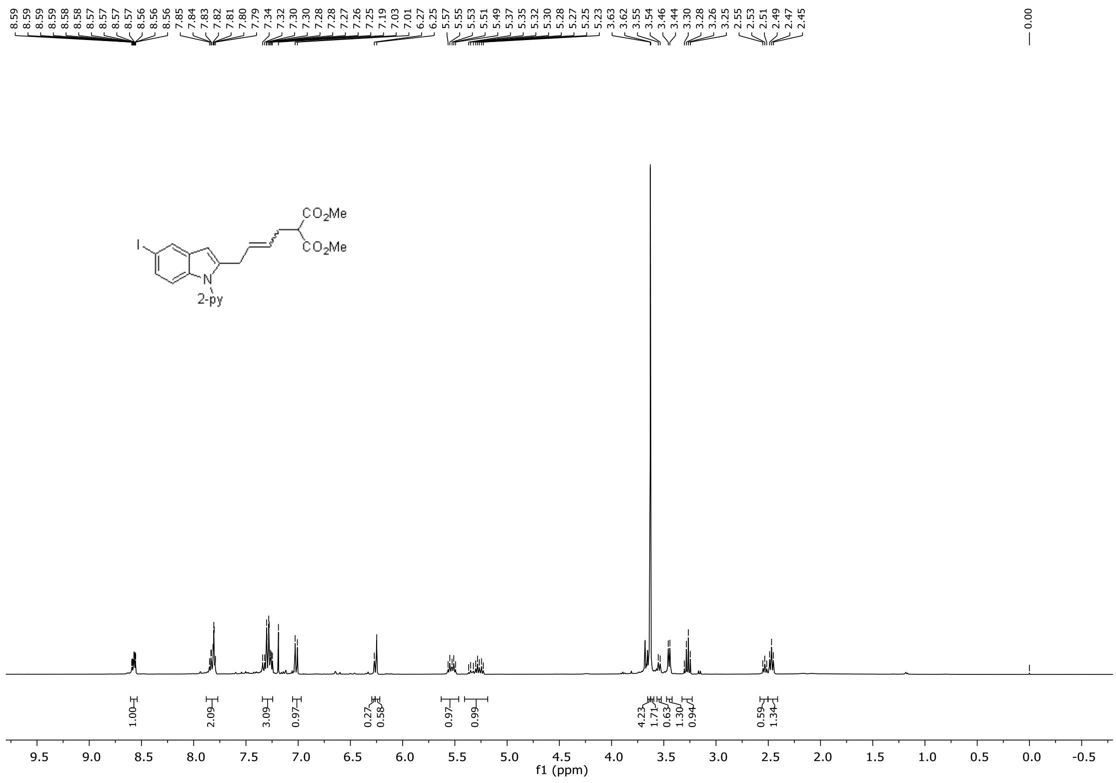

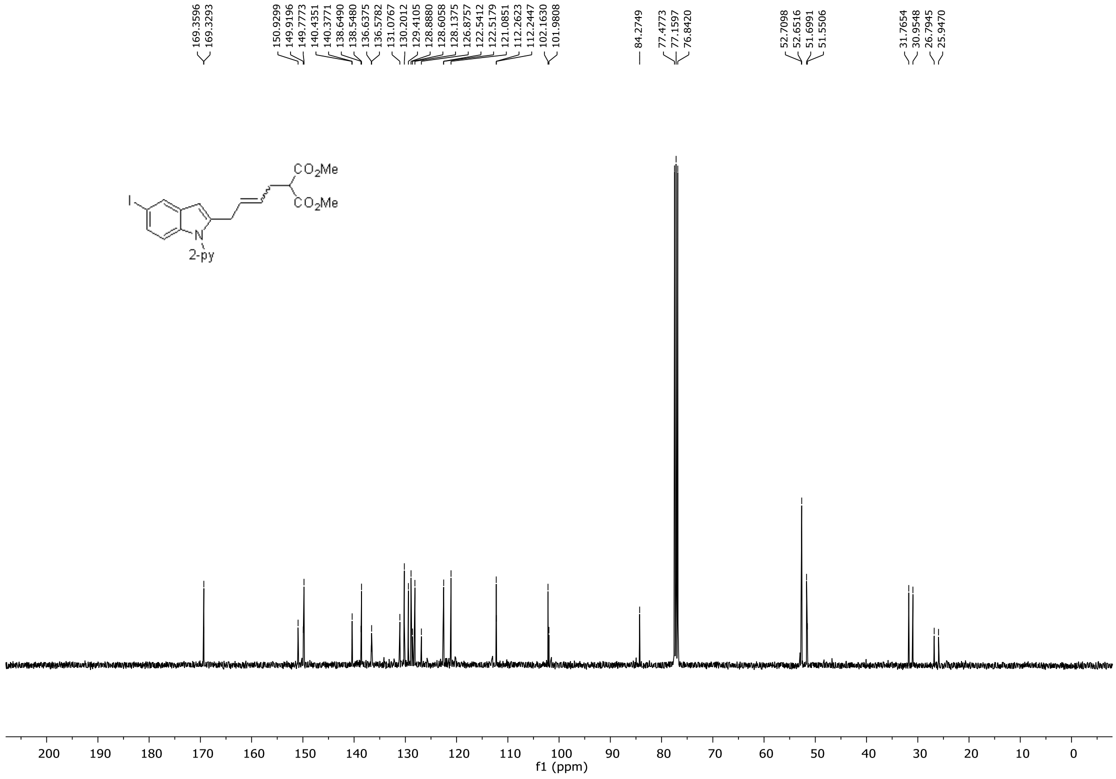

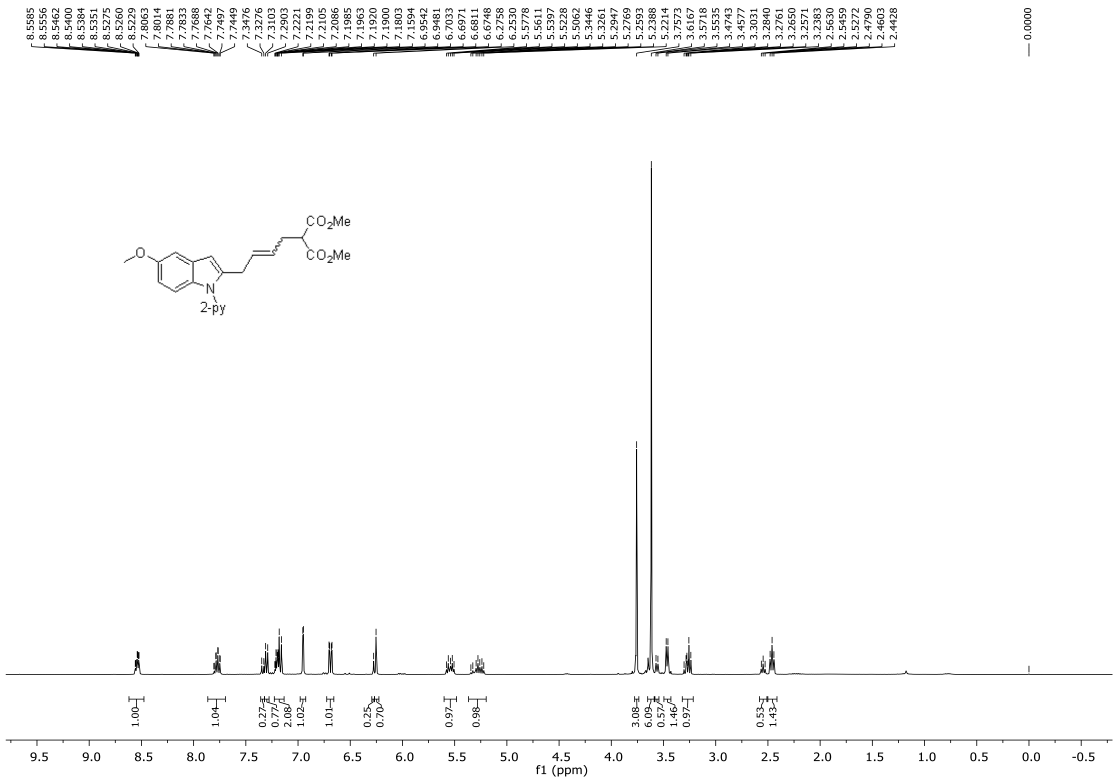

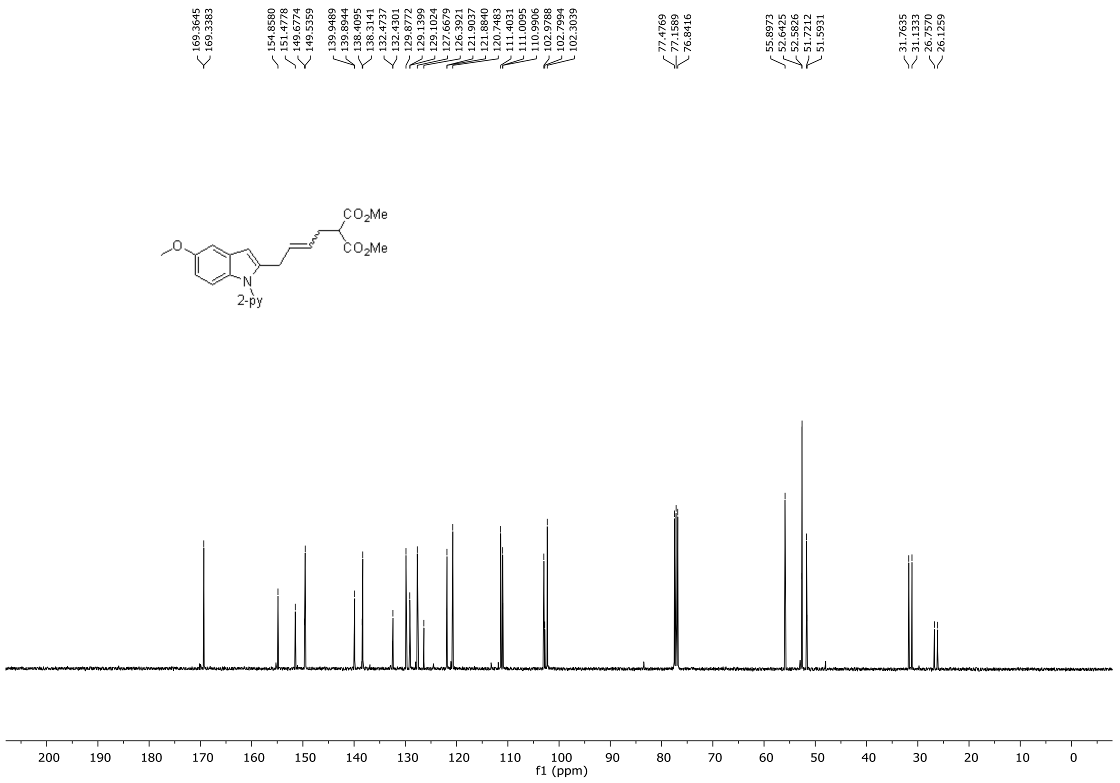

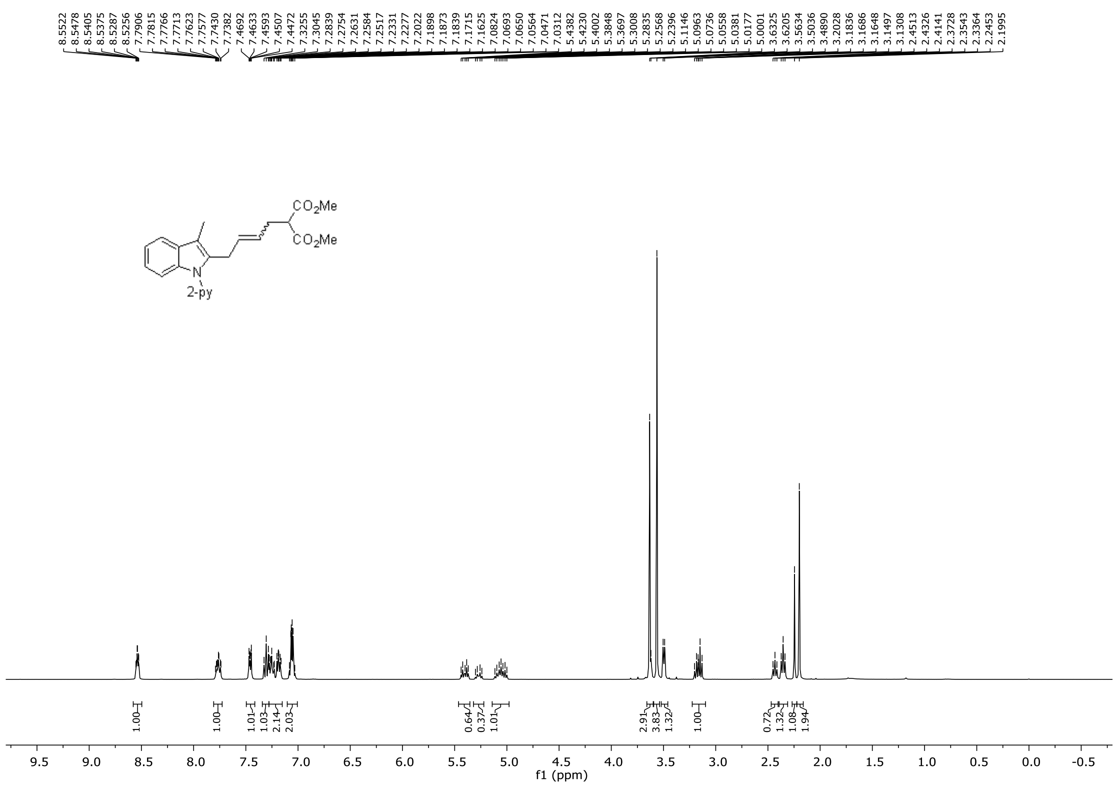

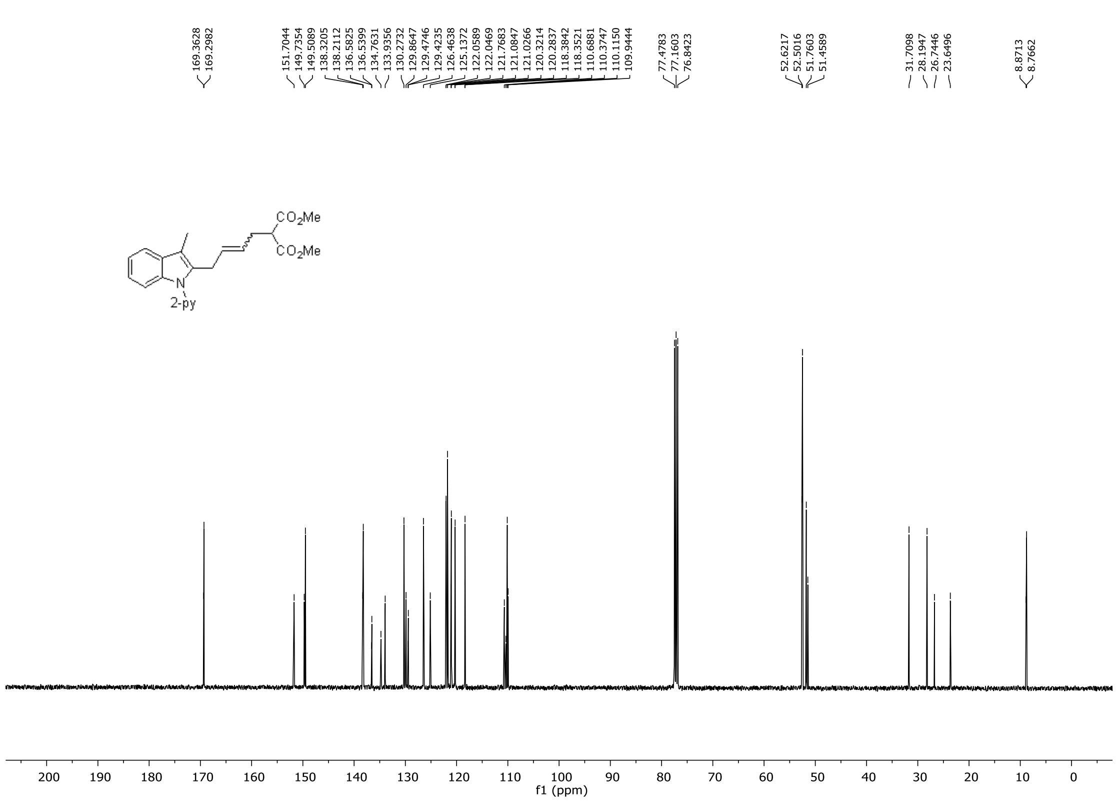

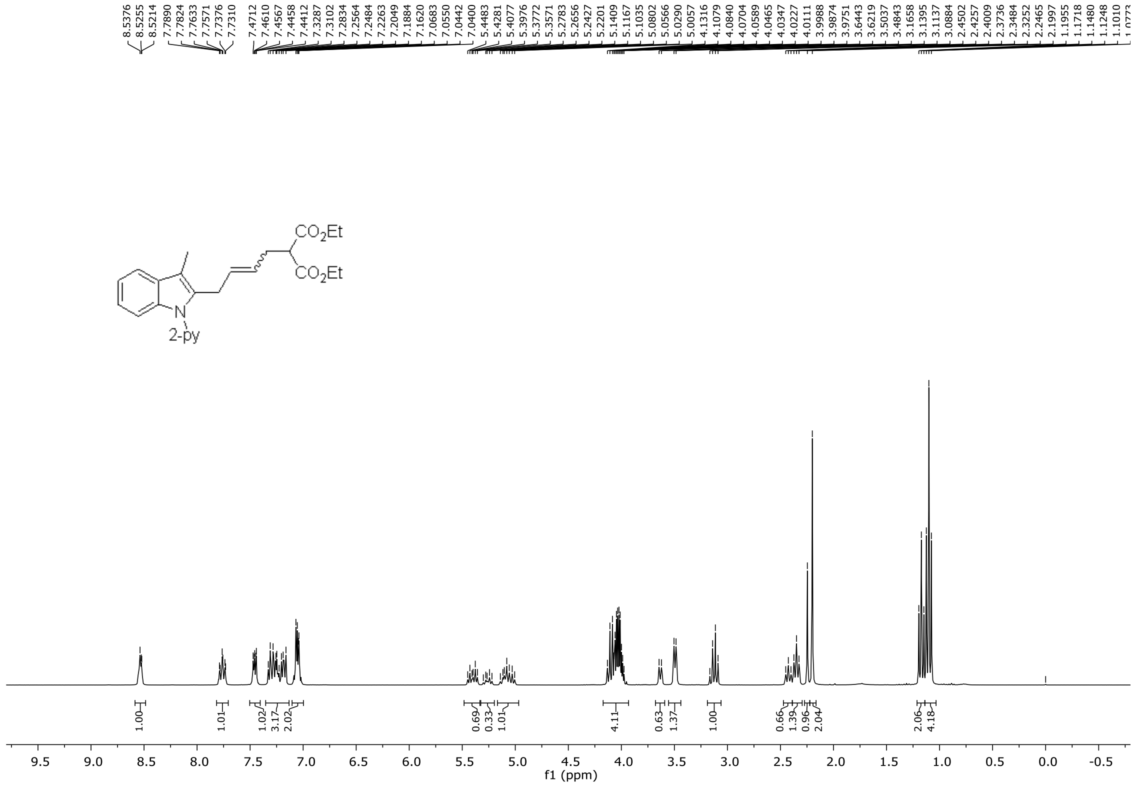
8.5376
8.5255
8.5214
7.7890
7.7824
7.7633
7.7571
7.7376
7.7310
7.4712
7.4610
7.4567
7.4458
7.4412
7.3287
7.3102
7.2834
7.2564
7.2484
7.2263
7.2049
7.1884
7.1620
7.0683
7.0550
7.0442
7.0400
5.4483
5.4281
5.4077
5.3976
5.3772
5.3571
5.2783
5.2656
5.2427
5.2201
5.1409
5.1167
5.1035
5.0802
5.0566
5.0290
5.0057
4.1316
4.1079
4.0840
4.0704
4.0589
4.0465
4.0347
4.0227
4.0111
3.9988
3.9874
3.9751
3.6443
3.6219
3.5037
3.4843
3.1658
3.1395
3.1137
3.0884
2.4502
2.4257
2.4009
2.3736
2.3484
2.3252
2.2465
2.1997
1.1955
1.1718
1.1480
1.1248
1.1010
CO2Et
CO2Et
N
2-py
1.00
1.01
1.02
3.17
2.02
0.69
0.33
1.01
4.11
0.63
1.37
1.00
0.66
1.39
0.96
2.04
2.06
4.18
9.5
9.0
8.5
8.0
7.5
7.0
6.5
6.0
5.5
5.0
4.5
4.0
3.5
3.0
2.5
2.0
1.5
1.0
0.5
0.0
-0.5
f1 (ppm)

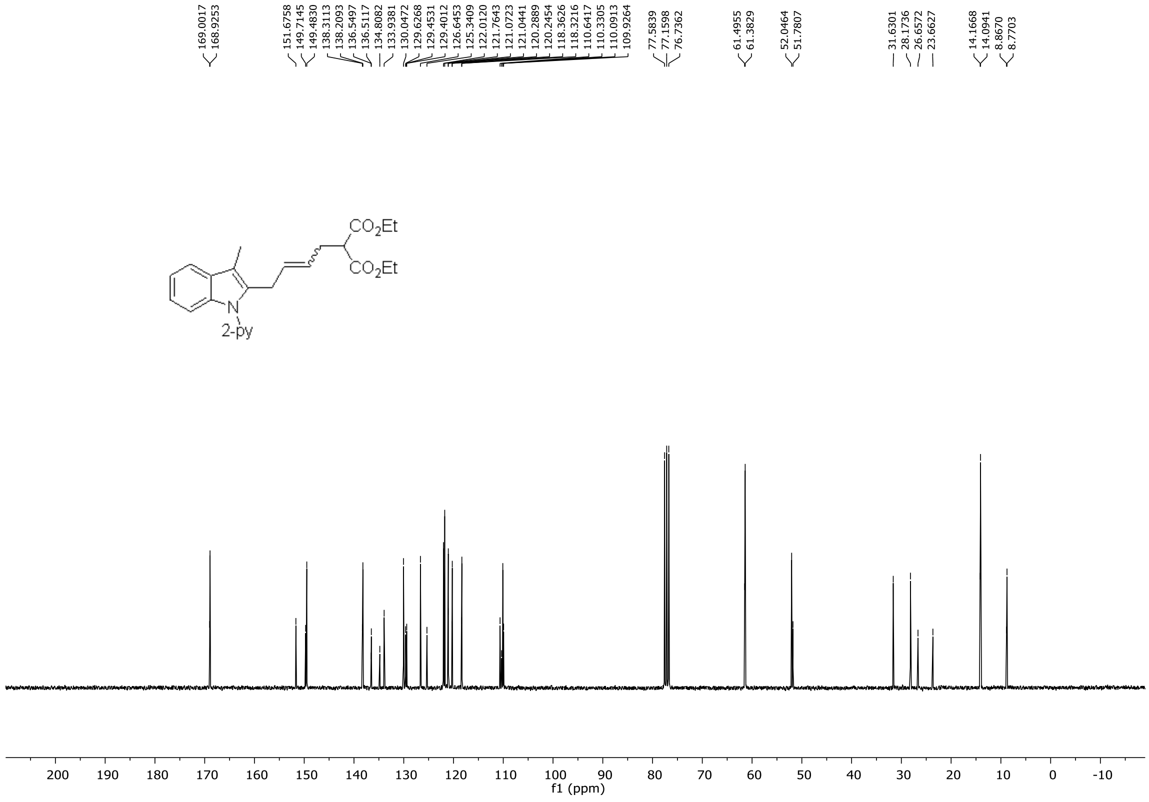
169.0017
168.9253
151.6758
149.7145
149.4830
138.3113
138.2093
136.5497
136.5117
134.8082
133.9381
130.0472
129.6268
129.4531
129.4012
126.6453
125.3409
122.0120
121.7643
121.0723
121.0441
120.2889
120.2454
118.3626
118.3216
110.6417
110.3305
110.0913
109.9264
77.5839
77.1598
76.7362
61.4955
61.3829
52.0464
51.7807
31.6301
28.1736
26.6572
23.6627
14.1668
14.0941
8.8670
8.7703
CO2Et
CO2Et
N
2-py
200
190
180
170
160
150
140
130
120
110
100
90
80
70
60
50
40
30
20
10
0
-10
f1 (ppm)

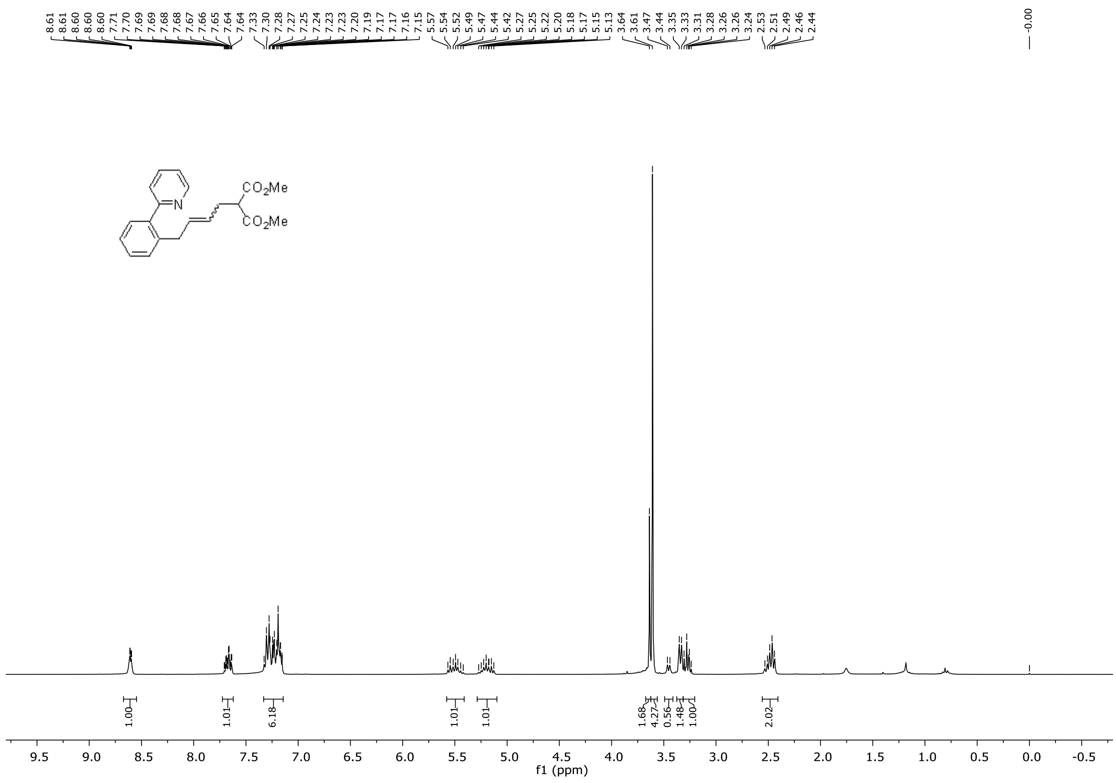

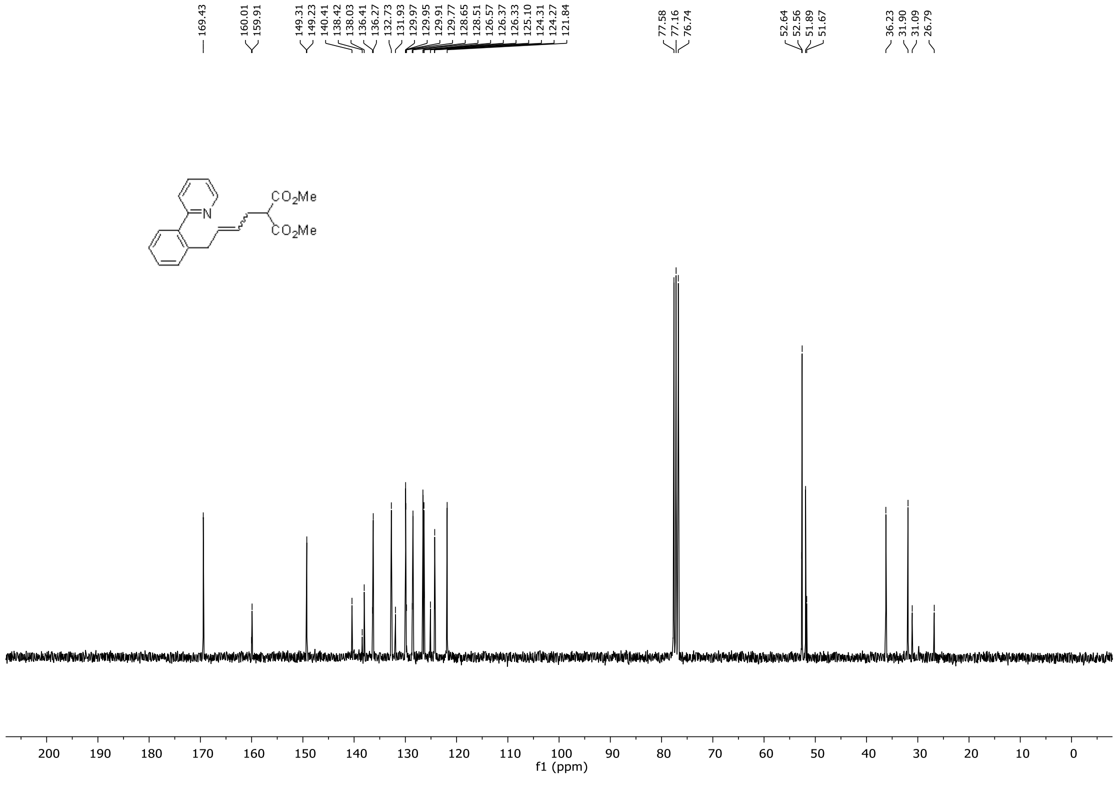

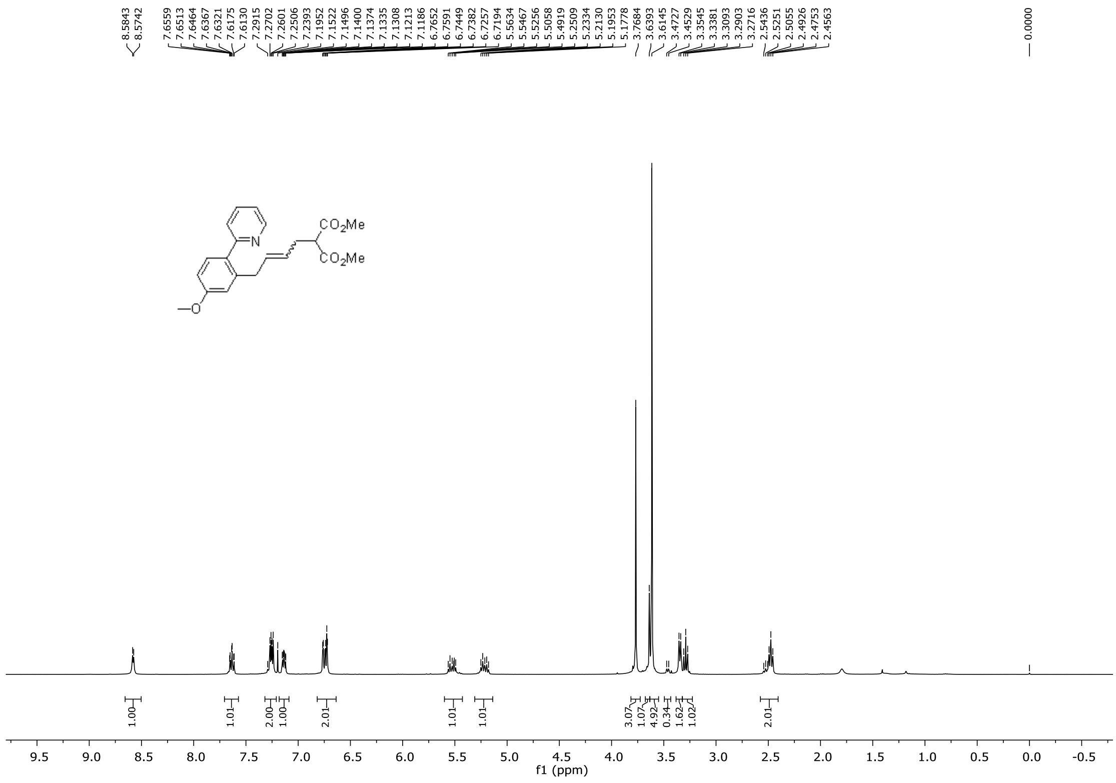

8.5843
8.5742
7.6559
7.6513
7.6464
7.6367
7.6321
7.6175
7.6130
7.2915
7.2702
7.2601
7.2506
7.2393
7.1952
7.1522
7.1496
7.1400
7.1374
7.1335
7.1308
7.1213
7.1186
6.7652
6.7591
6.7449
6.7382
6.7257
6.7194
5.5634
5.5467
5.5256
5.5058
5.4919
5.2509
5.2334
5.2130
5.1953
5.1778
3.7684
3.6393
3.6145
3.4727
3.4529
3.3545
3.3381
3.3093
3.2903
3.2716
2.5436
2.5251
2.5055
2.4926
2.4753
2.4563
0.0000
CO2Me
CO2Me
N
O
1.00
1.01
2.00
1.00
2.01
1.01
1.01
3.07
1.07
4.92
0.34
1.62
1.02
2.01
9.5 9.0 8.5 8.0 7.5 7.0 6.5 6.0 5.5 5.0 4.5 4.0 3.5 3.0 2.5 2.0 1.5 1.0 0.5 0.0 -0.5
f1 (ppm)

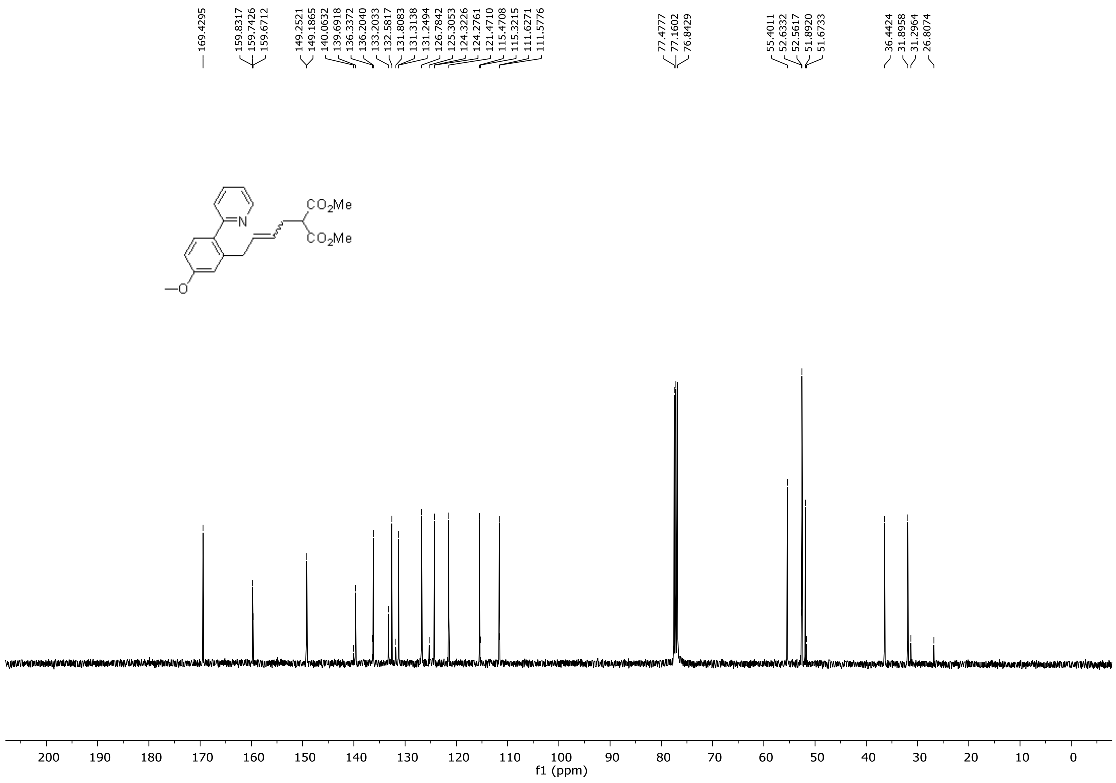

169.4295
159.8317
159.7426
159.6712
149.2521
149.1865
140.0632
139.6918
136.3372
136.2040
133.2033
132.5817
131.8083
131.3138
131.2494
126.7842
125.3053
124.3226
124.2761
121.4710
115.4708
115.3215
111.6271
111.5776
77.4777
77.1602
76.8429
55.4011
52.6332
52.5617
51.8920
51.6733
36.4424
31.8958
31.2964
26.8074
CO2Me
CO2Me
N
O
200 190 180 170 160 150 140 130 120 110 100 90 80 70 60 50 40 30 20 10 0
f1 (ppm)

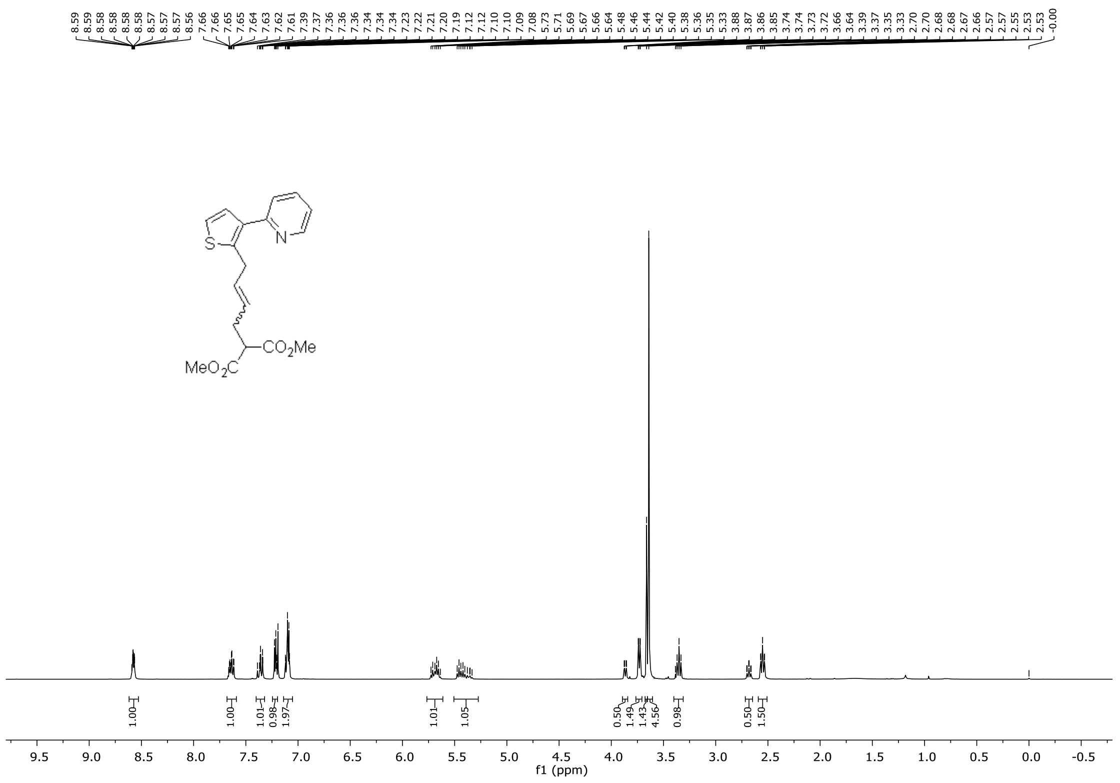
S
N
CO2Me
MeO2C
f1 (ppm)

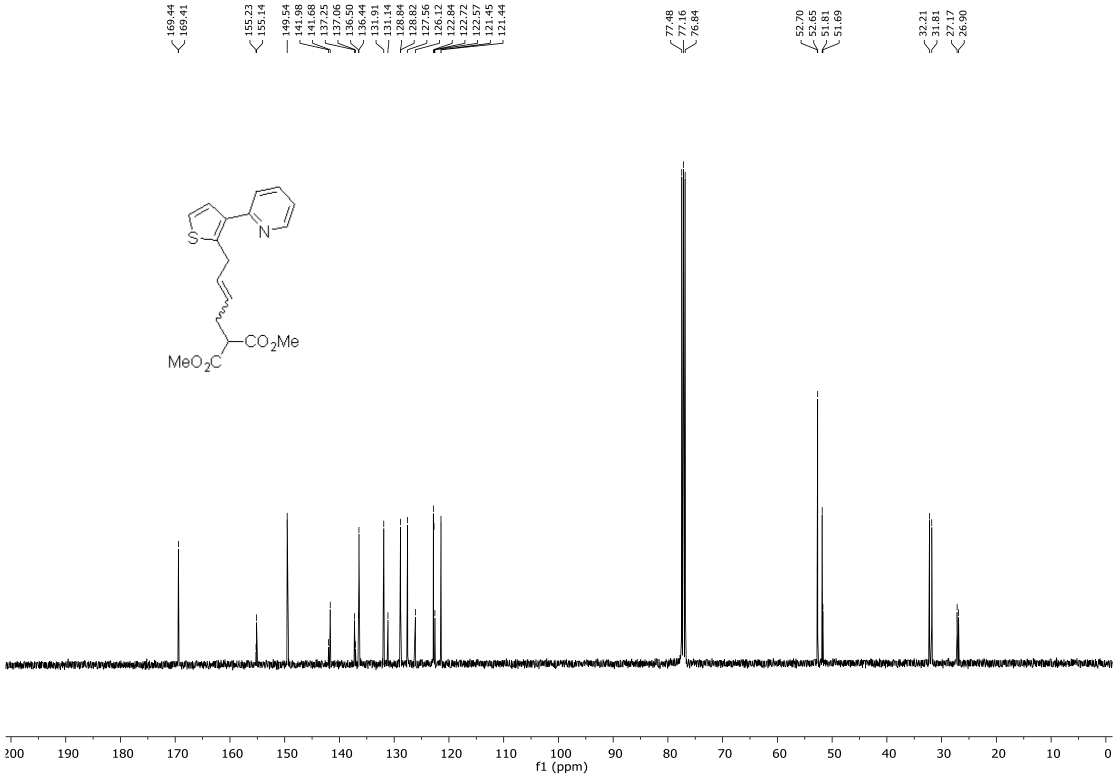
169.44
169.41
155.23
155.14
149.54
141.98
141.68
137.25
137.06
136.50
136.44
131.91
131.14
128.84
128.82
127.56
126.12
122.84
122.72
122.57
121.45
121.44
77.48
77.16
76.84
52.70
52.65
51.81
51.69
32.21
31.81
27.17
26.90
S
N
CO2Me
MeO2C
f1 (ppm)

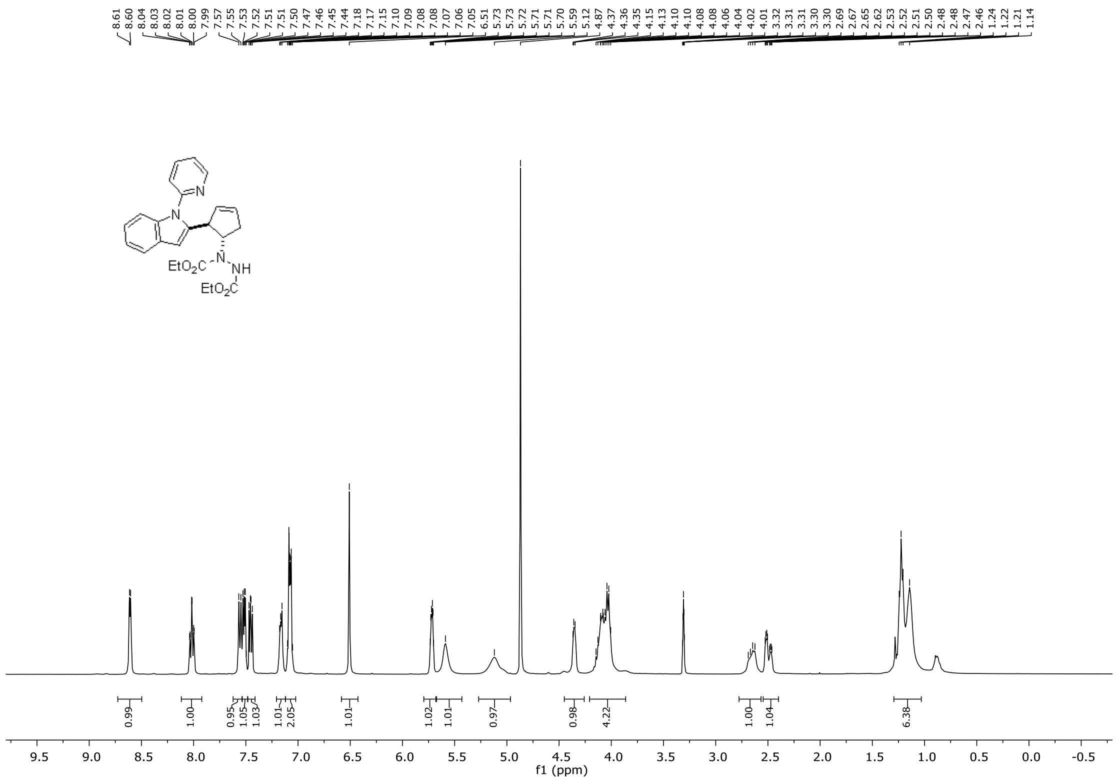

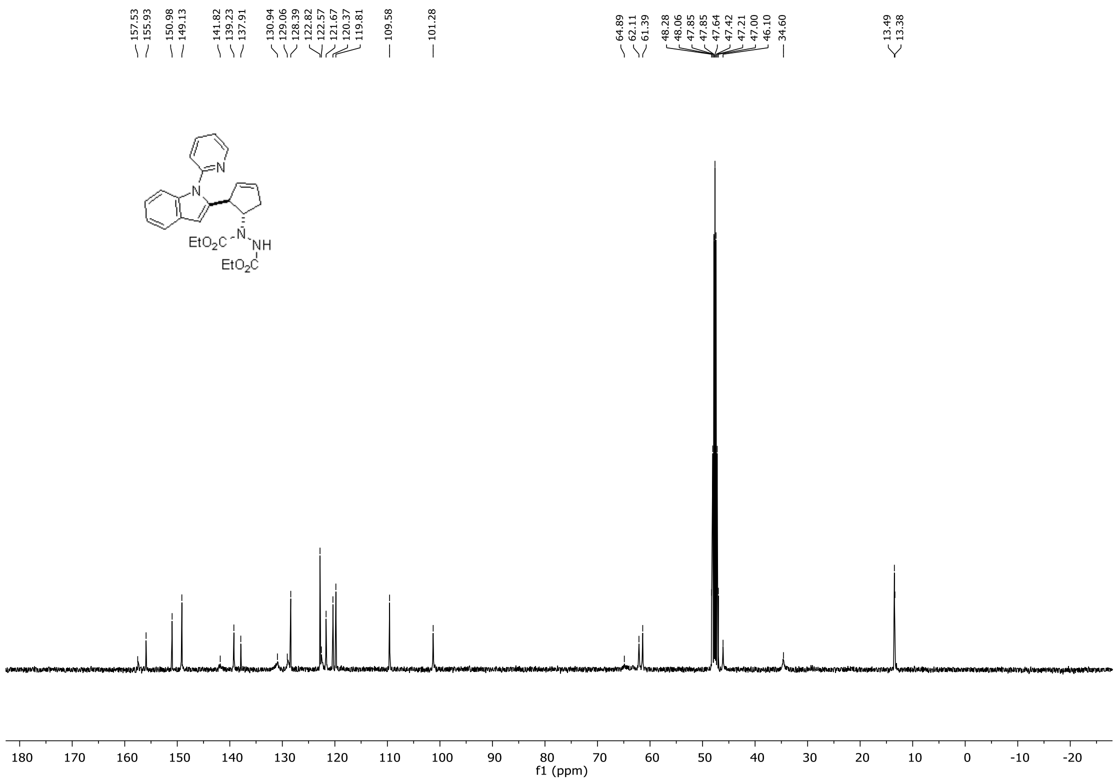

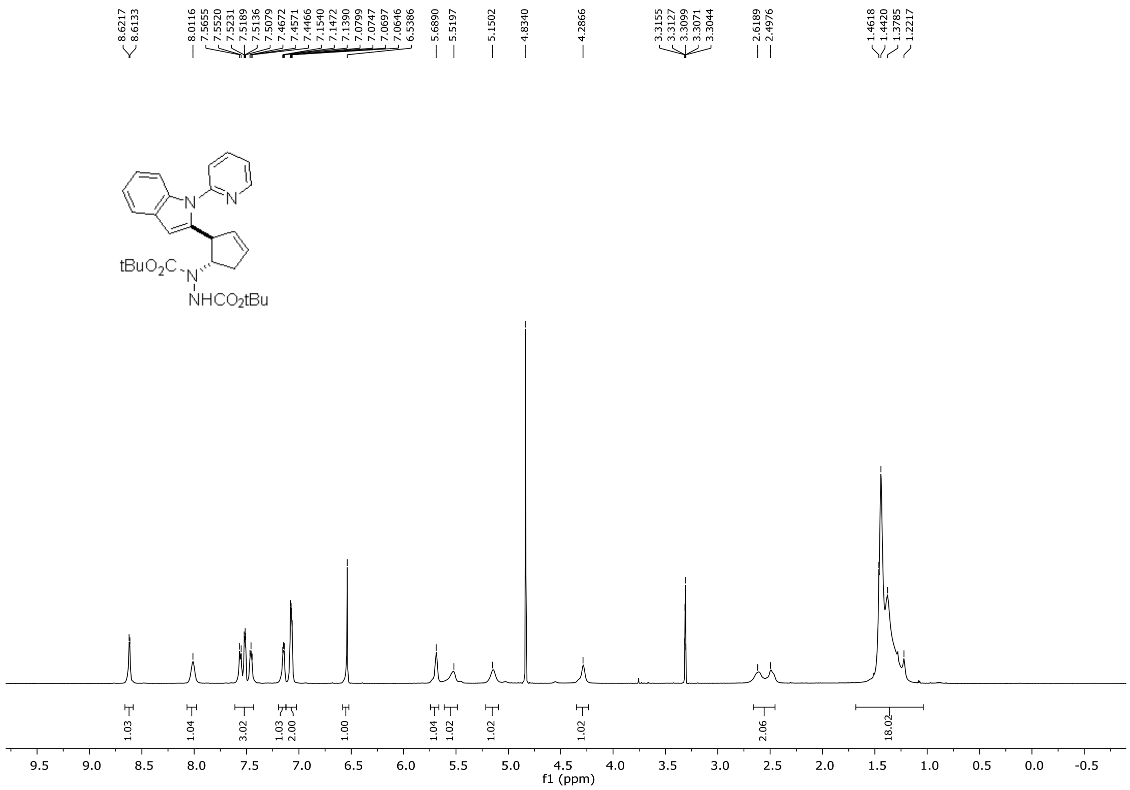
8.6217
8.6133
8.0116
7.5655
7.5520
7.5231
7.5189
7.5136
7.5079
7.4672
7.4571
7.4466
7.1540
7.1472
7.1390
7.0799
7.0747
7.0697
7.0646
6.5386
5.6890
5.5197
5.1502
4.8340
4.2866
3.3155
3.3127
3.3099
3.3071
3.3044
2.6189
2.4976
1.4618
1.4420
1.3785
1.2217
N
N
tBuO2C
N
NHCO2tBu
1.03
1.04
3.02
1.03
2.00
1.00
1.04
1.02
1.02
1.02
2.06
18.02
9.5
9.0
8.5
8.0
7.5
7.0
6.5
6.0
5.5
5.0
4.5
4.0
3.5
3.0
2.5
2.0
1.5
1.0
0.5
0.0
-0.5
f1 (ppm)

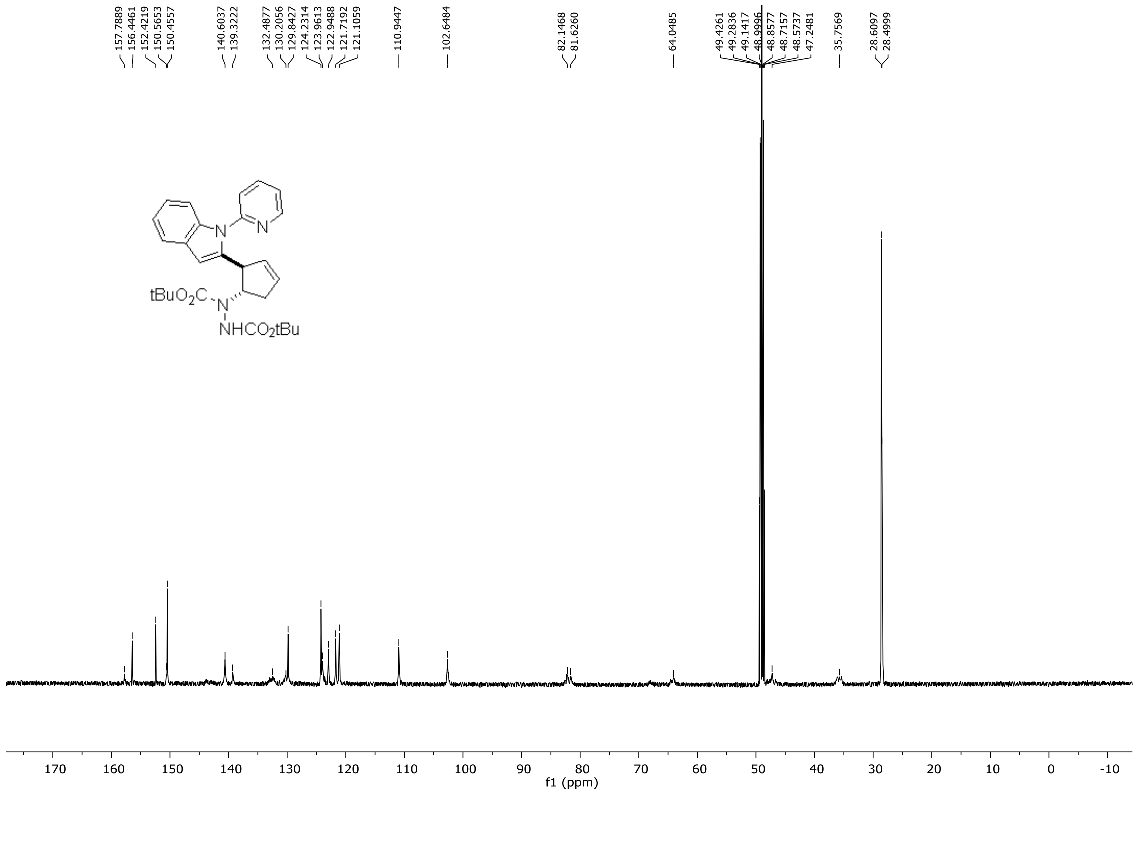
157.7889
156.4461
152.4219
150.5653
150.4557
140.6037
139.3222
132.4877
130.2056
129.8427
124.2314
123.9613
122.9488
121.7192
121.1059
110.9447
102.6484
82.1468
81.6260
64.0485
49.4261
49.2836
49.1417
48.9996
48.8577
48.7157
48.5737
47.2481
35.7569
28.6097
28.4999
N
N
tBuO2C
N
NHCO2tBu
170
160
150
140
130
120
110
100
90
80
70
60
50
40
30
20
10
0
-10
f1 (ppm)

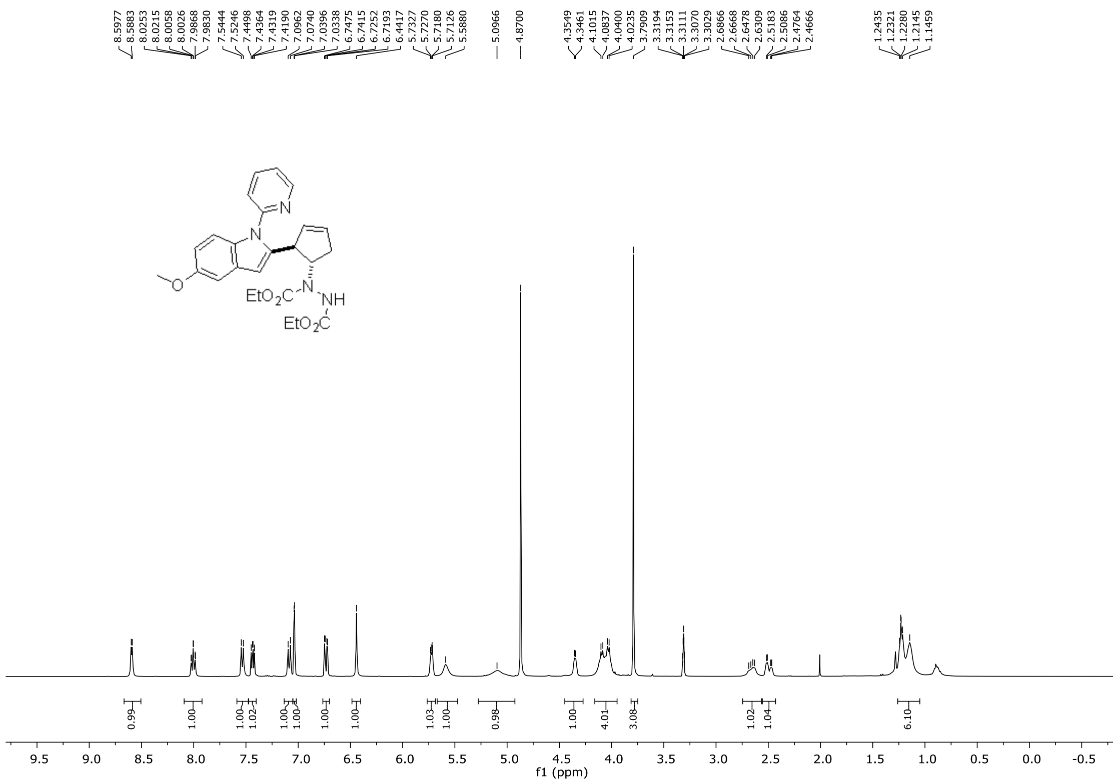
8.5977
8.5883
8.0253
8.0215
8.0058
8.0026
7.9868
7.9830
7.5444
7.5246
7.4498
7.4364
7.4319
7.4190
7.0962
7.0740
7.0396
7.0338
6.7475
6.7415
6.7252
6.7193
6.4417
5.7327
5.7270
5.7180
5.7126
5.5880
5.0966
4.8700
4.3549
4.3461
4.1015
4.0837
4.0400
4.0235
3.7909
3.3194
3.3153
3.3111
3.3070
3.3029
2.6866
2.6668
2.6478
2.6309
2.5183
2.5086
2.4764
2.4666
1.2435
1.2321
1.2280
1.2145
1.1459
N
N
O
EtO2C
N
NH
EtO2C
0.99
1.00
1.00
1.02
1.00
1.00
1.00
1.00
1.03
1.00
0.98
1.00
4.01
3.08
1.02
1.04
6.10
9.5
9.0
8.5
8.0
7.5
7.0
6.5
6.0
5.5
5.0
4.5
4.0
3.5
3.0
2.5
2.0
1.5
1.0
0.5
0.0
-0.5
f1 (ppm)

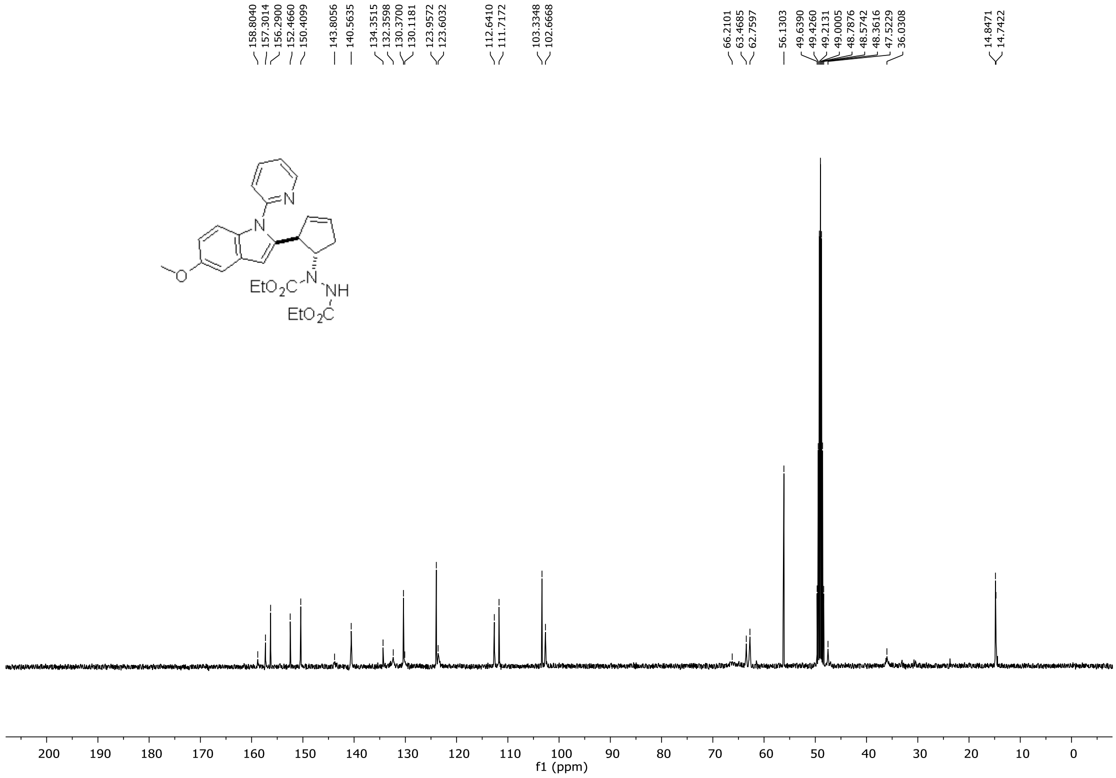
158.8040
157.3014
156.2900
152.4660
150.4099
143.8056
140.5635
134.3515
132.3598
130.3700
130.1181
123.9572
123.6032
112.6410
111.7172
103.3348
102.6668
66.2101
63.4685
62.7597
56.1303
49.6390
49.4260
49.2131
49.0005
48.7876
48.5742
48.3616
47.5229
36.0308
14.8471
14.7422
N
N
O
EtO2C
N
NH
EtO2C
200
190
180
170
160
150
140
130
120
110
100
90
80
70
60
50
40
30
20
10
0
f1 (ppm)

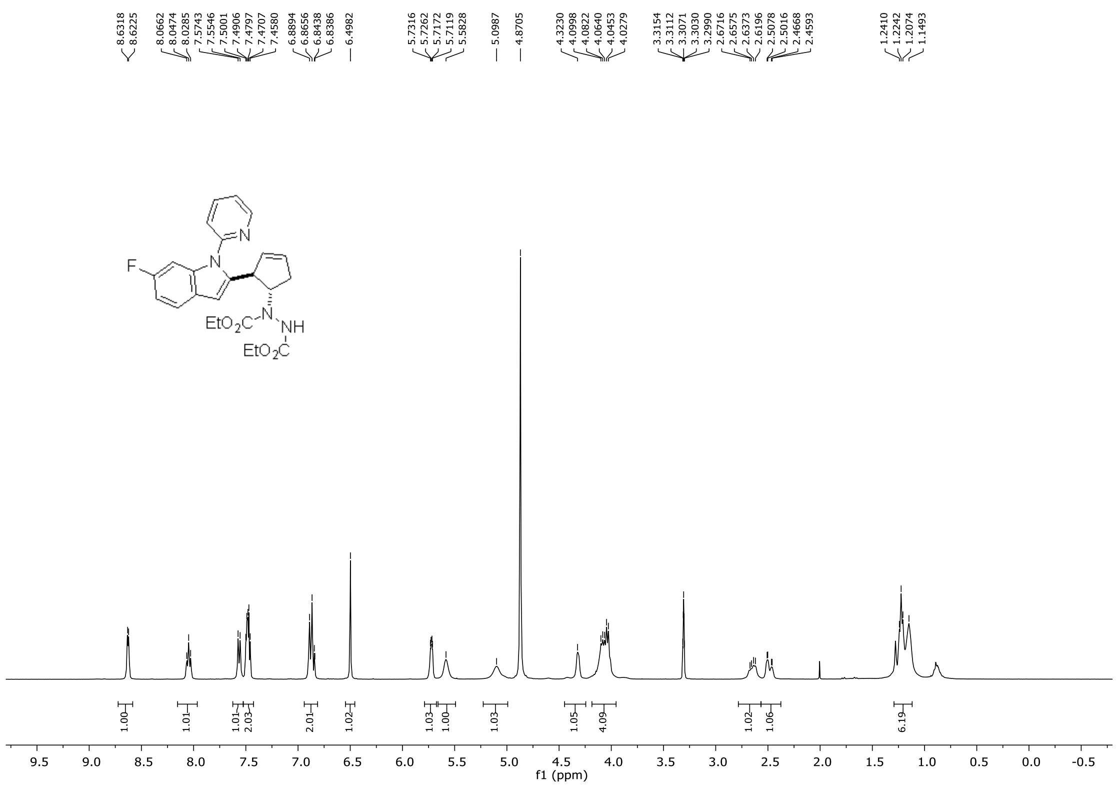

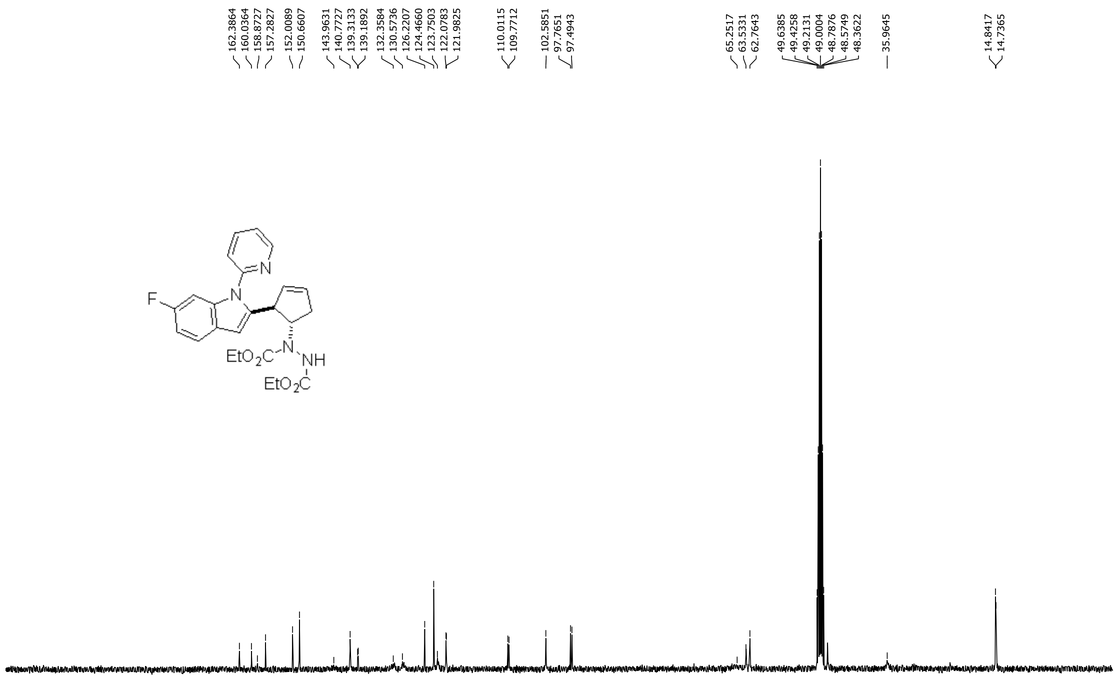

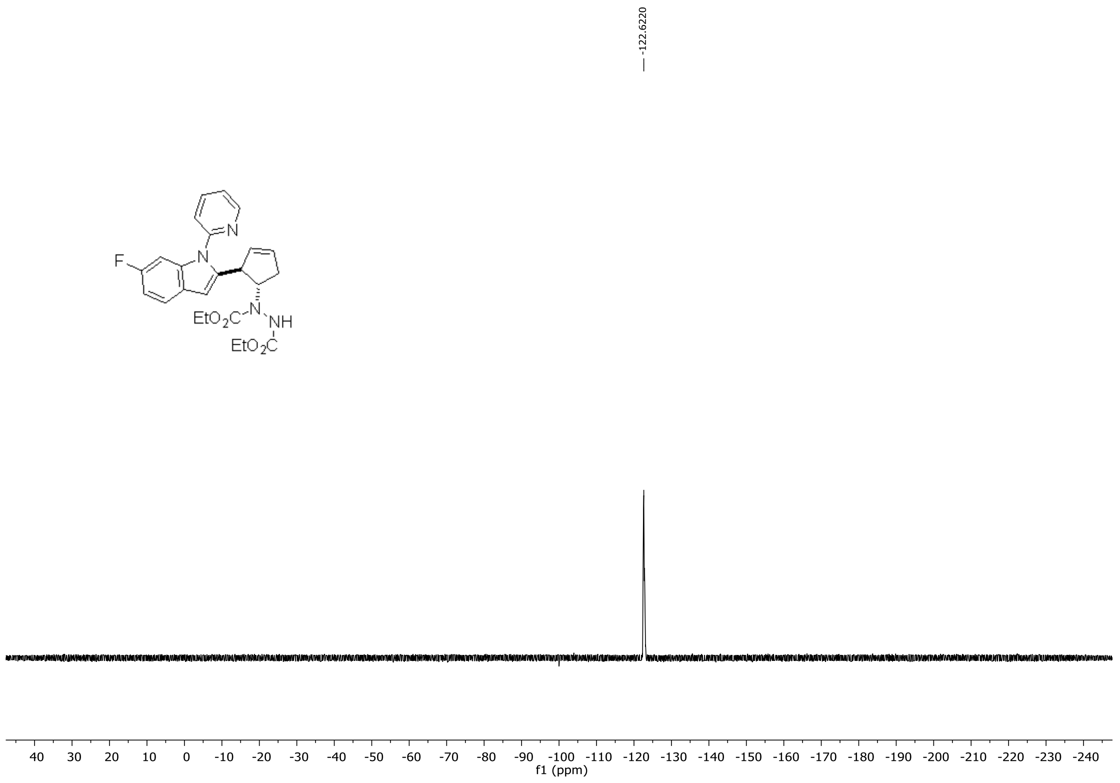
-122.6220
F
N
N
EtO2C
N
NH
EtO2C
40 30 20 10 0 -10 -20 -30 -40 -50 -60 -70 -80 -90 -100 -110 -120 -130 -140 -150 -160 -170 -180 -190 -200 -210 -220 -230 -240
f1 (ppm)

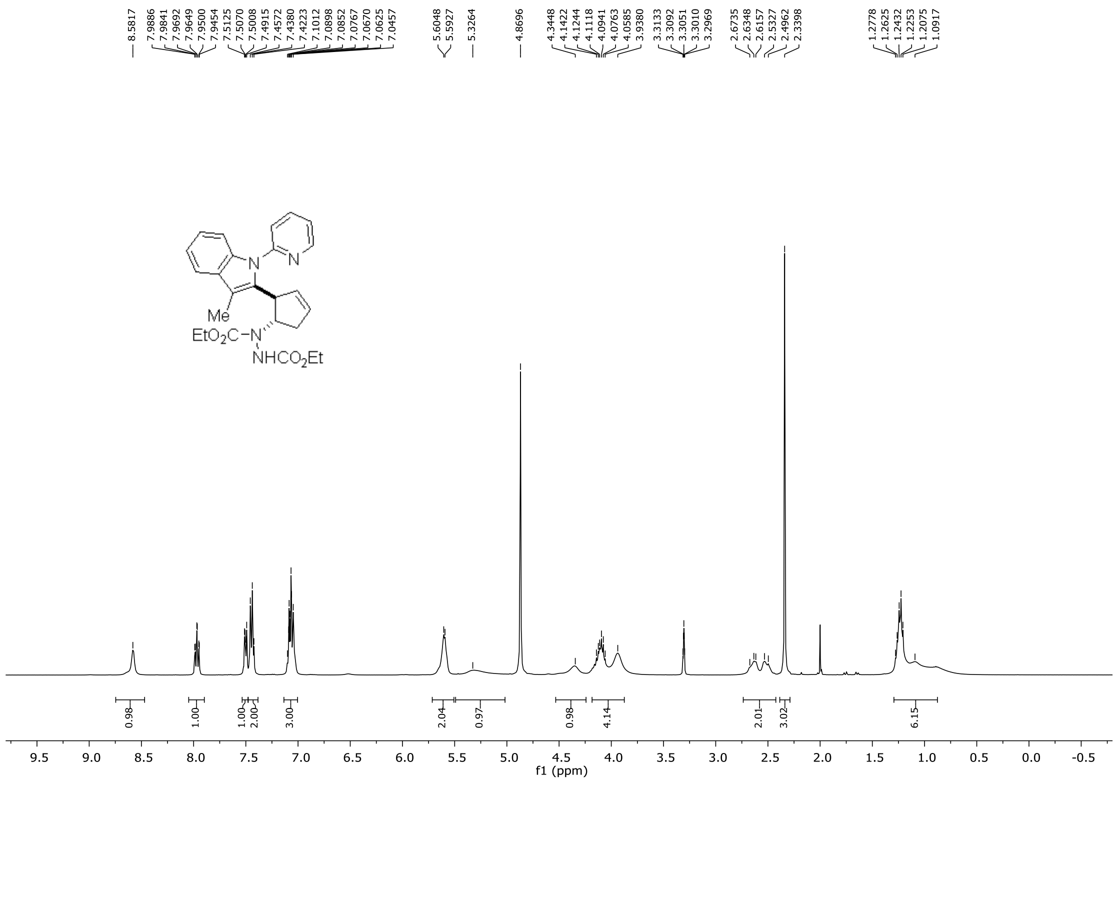
8.5817
7.9886
7.9841
7.9692
7.9649
7.9500
7.9454
7.5125
7.5070
7.5008
7.4915
7.4572
7.4380
7.4223
7.1012
7.0898
7.0852
7.0767
7.0670
7.0625
7.0457
5.6048
5.5927
5.3264
4.8696
4.3448
4.1422
4.1244
4.1118
4.0941
4.0763
4.0585
3.9380
3.3133
3.3092
3.3051
3.3010
3.2969
2.6735
2.6348
2.6157
2.5327
2.4962
2.3398
1.2778
1.2625
1.2432
1.2253
1.2075
1.0917
N
N
Me
EtO2C
N
NHCO2Et
0.98
1.00
1.00
2.00
3.00
2.04
0.97
0.98
4.14
2.01
3.02
6.15
9.5 9.0 8.5 8.0 7.5 7.0 6.5 6.0 5.5 5.0 4.5 4.0 3.5 3.0 2.5 2.0 1.5 1.0 0.5 0.0 -0.5
f1 (ppm)

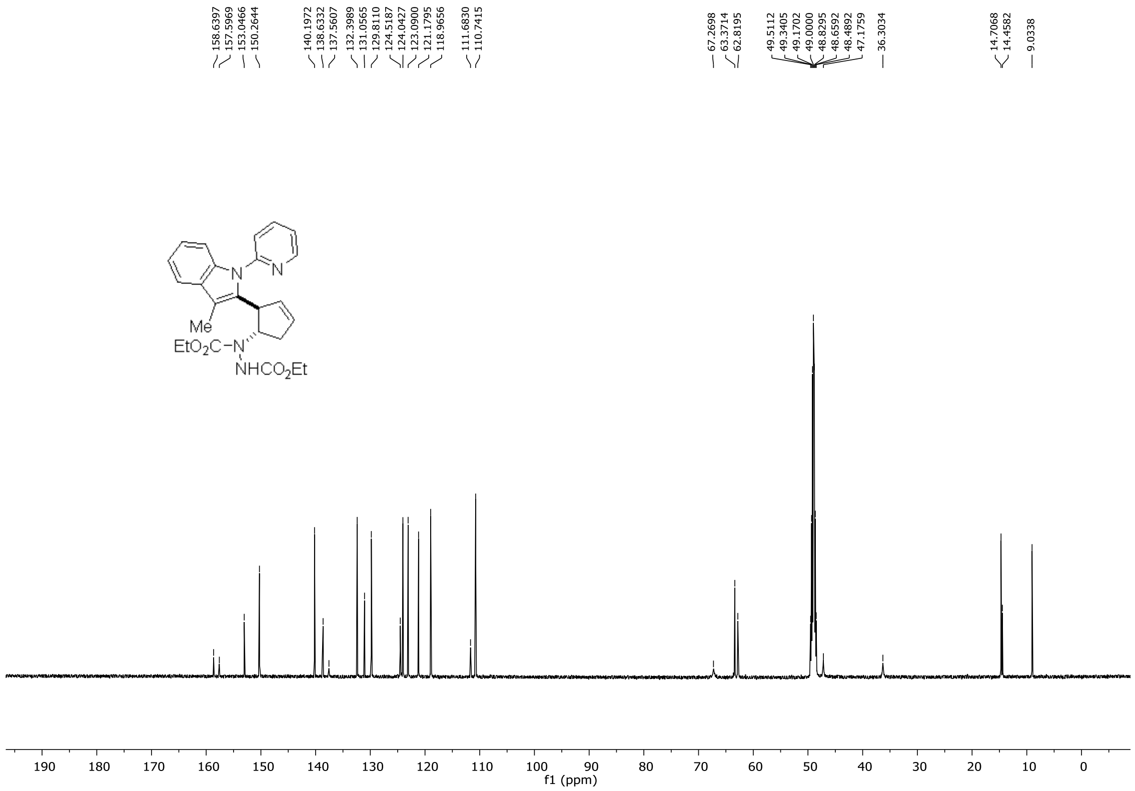

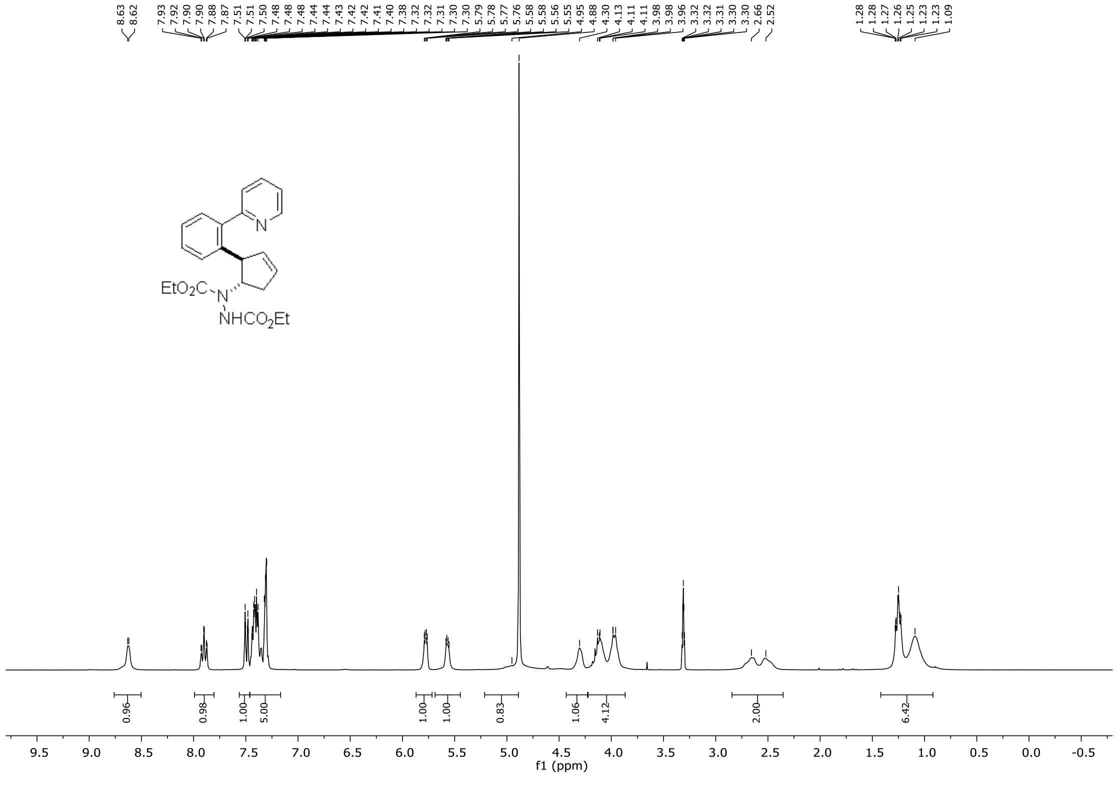

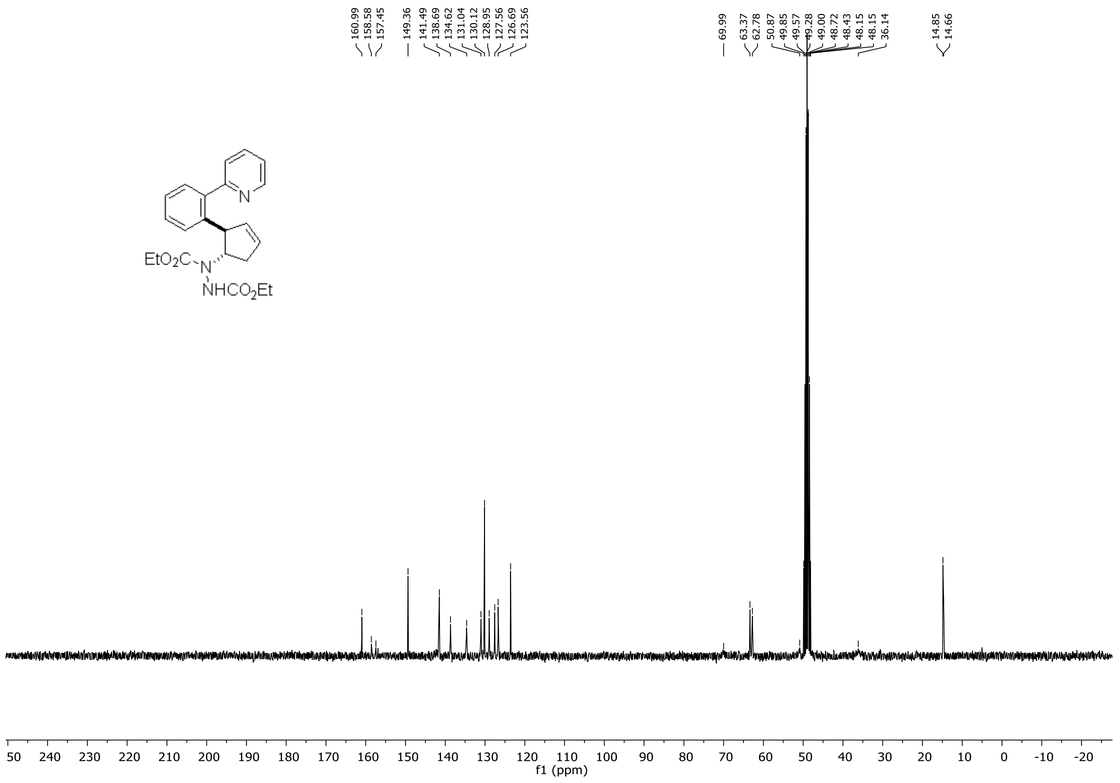
160.99
158.58
157.45
149.36
141.49
138.69
134.62
131.04
130.12
128.95
127.56
126.69
123.56
69.99
63.37
62.78
50.87
49.85
49.57
49.28
49.00
48.72
48.43
48.15
36.14
14.85
14.66
EtO2C
N
NHCO2Et
N
f1 (ppm)

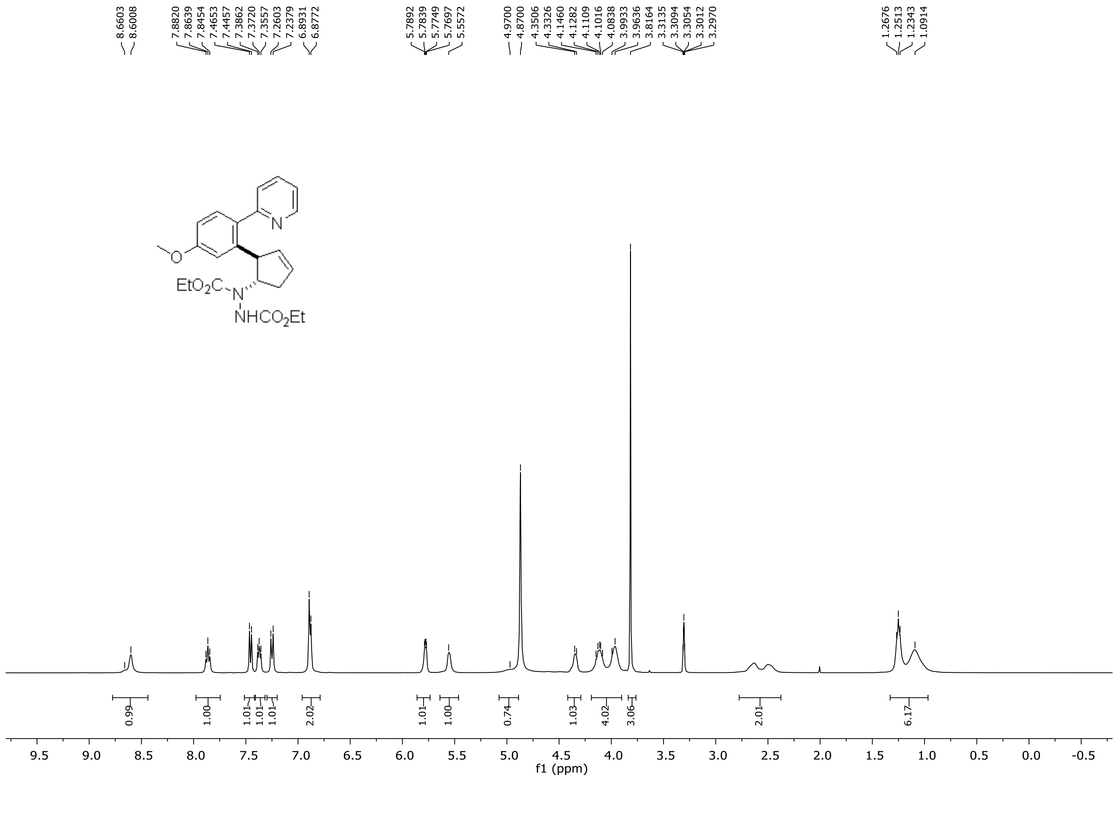
8.6603
8.6008
7.8820
7.8639
7.8454
7.4653
7.4457
7.3862
7.3720
7.3557
7.2603
7.2379
6.8931
6.8772
5.7892
5.7839
5.7749
5.7697
5.5572
4.9700
4.8700
4.3506
4.3326
4.1460
4.1282
4.1109
4.1016
4.0838
3.9933
3.9636
3.8164
3.3135
3.3094
3.3054
3.3012
3.2970
1.2676
1.2513
1.2343
1.0914
EtO2C
N
NHCO2Et
N
0.99
1.00
1.01
1.01
1.01
2.02
1.01
1.00
0.74
1.03
4.02
3.06
2.01
6.17
f1 (ppm)

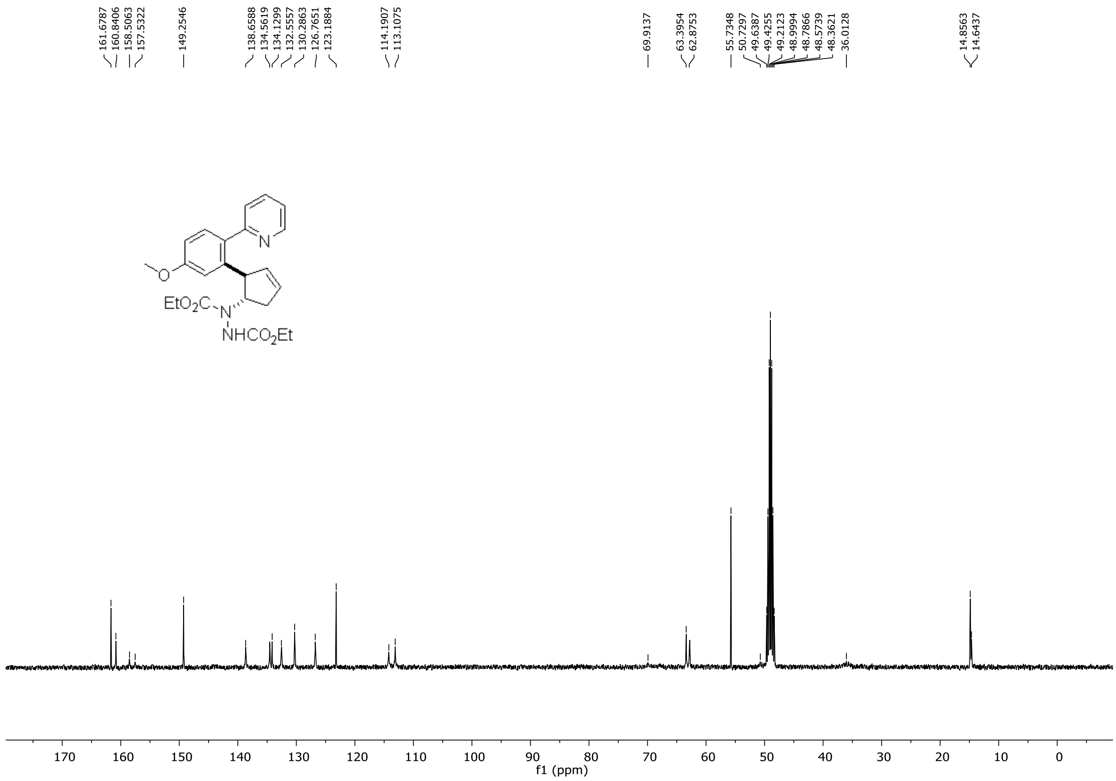
161.6787
160.8406
158.5063
157.5322
149.2546
138.6588
134.5619
134.1299
132.5557
130.2863
126.7651
123.1884
114.1907
113.1075
69.9137
63.3954
62.8753
55.7348
50.7297
49.6387
49.4255
49.2123
48.9994
48.7866
48.5739
48.3621
36.0128
14.8563
14.6437
EtO2C
N
NHCO2Et
N
f1 (ppm)

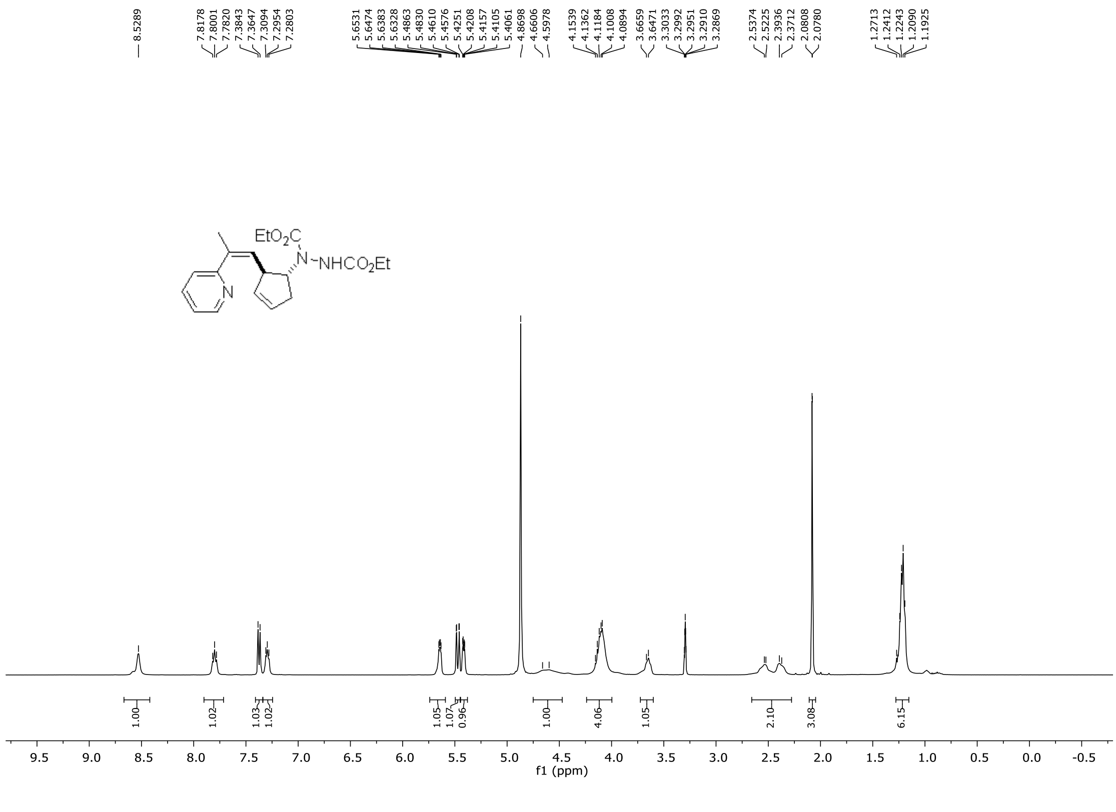
8.5289
7.8178
7.8001
7.7820
7.3843
7.3647
7.3094
7.2954
7.2803
5.6531
5.6474
5.6383
5.6328
5.4863
5.4830
5.4610
5.4576
5.4251
5.4208
5.4157
5.4105
5.4061
4.8698
4.6606
4.5978
4.1539
4.1362
4.1184
4.1008
4.0894
3.6659
3.6471
3.3033
3.2992
3.2951
3.2910
3.2869
2.5374
2.5225
2.3936
2.3712
2.0808
2.0780
1.2713
1.2412
1.2243
1.2090
1.1925
EtO2C
N
NHCO2Et
N
1.00
1.02
1.03
1.02
1.05
1.07
0.96
1.00
4.06
1.05
2.10
3.08
6.15
f1 (ppm)

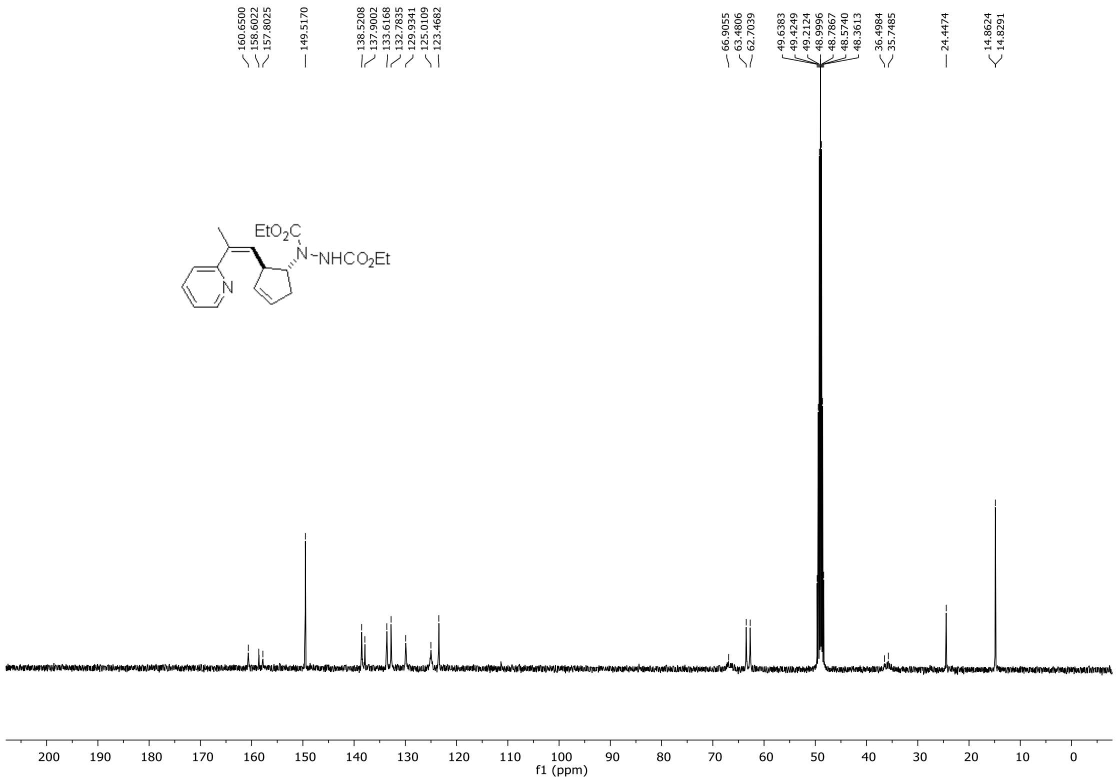
160.6500
158.6022
157.8025
149.5170
138.5208
137.9002
133.6168
132.7835
129.9341
125.0109
123.4682
66.9055
63.4806
62.7039
49.6383
49.4249
49.2124
48.9996
48.7867
48.5740
48.3613
36.4984
35.7485
24.4474
14.8624
14.8291
EtO2C
N
NHCO2Et
N
200 190 180 170 160 150 140 130 120 110 100 90 80 70 60 50 40 30 20 10 0
f1 (ppm)
